# Paleoenvironment and Organic Characterization of the Lower Cretaceous Lacustrine Source Rocks in the Erlian Basin: The Influence of Hydrothermal and Volcanic Activity on the Source Rock Quality

**DOI:** 10.1021/acsomega.2c03487

**Published:** 2023-01-03

**Authors:** Piao Wu, Hou Dujie, Lanzhu Cao, Ronghua Zheng, Xiuli Wei, Xiaoxiao Ma, Zhe Zhao, Jianwen Chen

**Affiliations:** † Qingdao Institute of Marine Geology, Qingdao 266237, China; ‡ Laboratory for Marine Mineral Resources, Laoshan Laboratory, Qingdao 266237, China; § School of Energy Resources, China University of Geosciences, Beijing 100083, China; ∥ Research Institute of Petroleum Exploration and Development, PetroChina Huabei Oilfield Company, Renqiu 062550, China

## Abstract

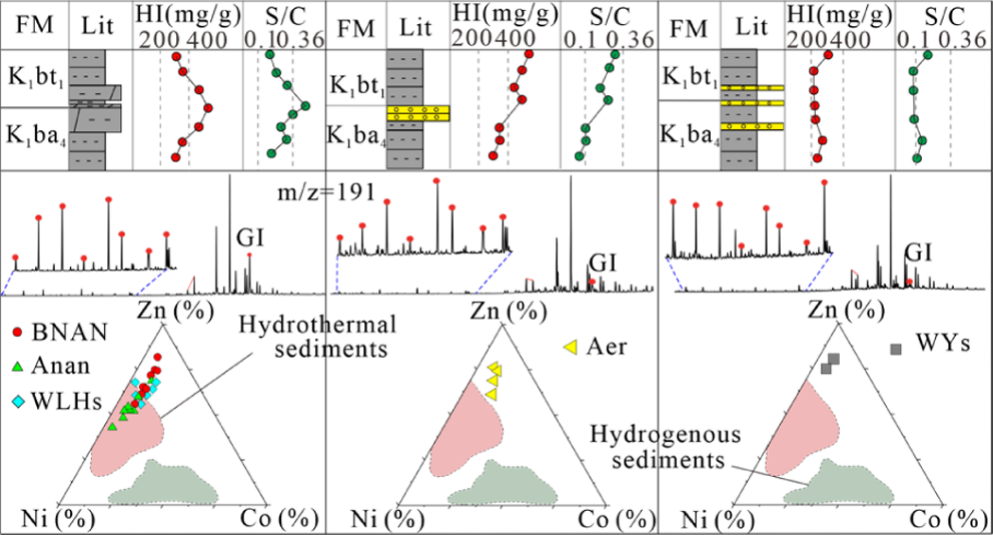

Lower Cretaceous lacustrine source rocks in the Erlian Basin are highly heterogeneous. It is important to assess and explain these heterogeneities for the reconstruction of paleoenvironments and the prediction of high-quality source rock distributions. In this study, well-logging, organic, and elemental geochemical data were comprehensively analyzed for the source rocks of Member 4 of the Aershan Formation (Fm) and Member 1 of the Tengger Fm in the southern Bayindulan (BNAN), southern Wulanhua (WLHs), Anan, Aer, and southern Wuliyasitai sags of the Erlian Basin. The variability in sedimentary environments, sources of organic matter of the source rocks in different sags, and the influence of hydrothermal and volcanic activity on the source rock quality in the Erlian Basin were assessed. The results reveal that the source rocks can be divided into four types of organic facies (A, B, BC, and C). Organic facies A–B present hydrogen indices (HIs) higher than 400 mg/g and are mainly composed of mudstone and thick (average thickness >50 m) dolomitic mudstone, with biomarkers characterized by a Pr/Ph ratio lower than 1.0, a gammacerane/C_30_ hopane (Gam/C_30_H) ratio higher than 0.2, and a C_19_ tricyclic terpane/C_23_ tricyclic terpane (C_19_/C_23_TT) ratio lower than 0.6. Organic facies BC–C are composed of mudstone with an HI < 400 mg/g, with biomarkers characterized by a Pr/Ph ratio higher than 0.8, a Gam/C_30_H ratio lower than 0.2, a C_19_/C_23_TT ratio higher than 0.6, and a sterane/hopane ratio lower than 0.4. Dolomitic mudstone belonging to organic facies A–B is mainly developed in the BNAN, WLHs, and Anan sag and is characterized by a fault-controlled distribution in the sag, a right-declined rare earth element pattern, and an enrichment in the elements of Ba, Cu, Zn, Fe, and Ni. The genesis of high HI dolomitic mudstone is associated with hydrothermal and volcanic activity because the hydrothermal fluid or hydrolysis of volcanic ash result in increasing input of reducing gas and soluble nutrient ions, thus promoting the formation of anoxic and saline Cretaceous lakes with high primary productivity.

## 1 Introduction

The symbiosis between volcanic rocks and source rocks is common in the rift petroliferous basins of eastern China,^1^ but views concerning the influence of volcanic activity on organic matter enrichment in source rocks vary greatly. Numerous studies have shown that volcanic ash produced by volcanic eruptions can release large amounts of Fe, N, P, Si, Mn, and other nutrients into the water column, which is favorable for the proliferation of algae,^2^ fungi,^3^ or other organisms^4,5^ and further promote primary productivity.^6^ Several studies have also shown that soluble gases (SO_2_, H_2_S, etc.) and metal elements (Ca^2+^, Mg^2+^, etc.) released by volcanic eruption increase water reducibility^7,8^ and salinity,^9,10^ which are conducive to the stratification of the water column and may further promote the preservation of organic matter.^11^ The gases and dust produced during intense volcanic eruptions also form sulfate aerosols and accelerate the cooling of the climate through an aerosol-cloud-climate feedback system,^12,13^ which would result in mass extinctions^14,15^ and thus facilitates the burial of organic matter.^11^ However, some scholars believe that the benefits of volcanic ash on organic matter enrichment are not clear^16,17^ and intense water turbulence or the dilution effect caused by volcanic eruption or homeochronous hydrothermal exhalation may even cease the enrichment of organic matter.^13,18^

**Figure 1:**
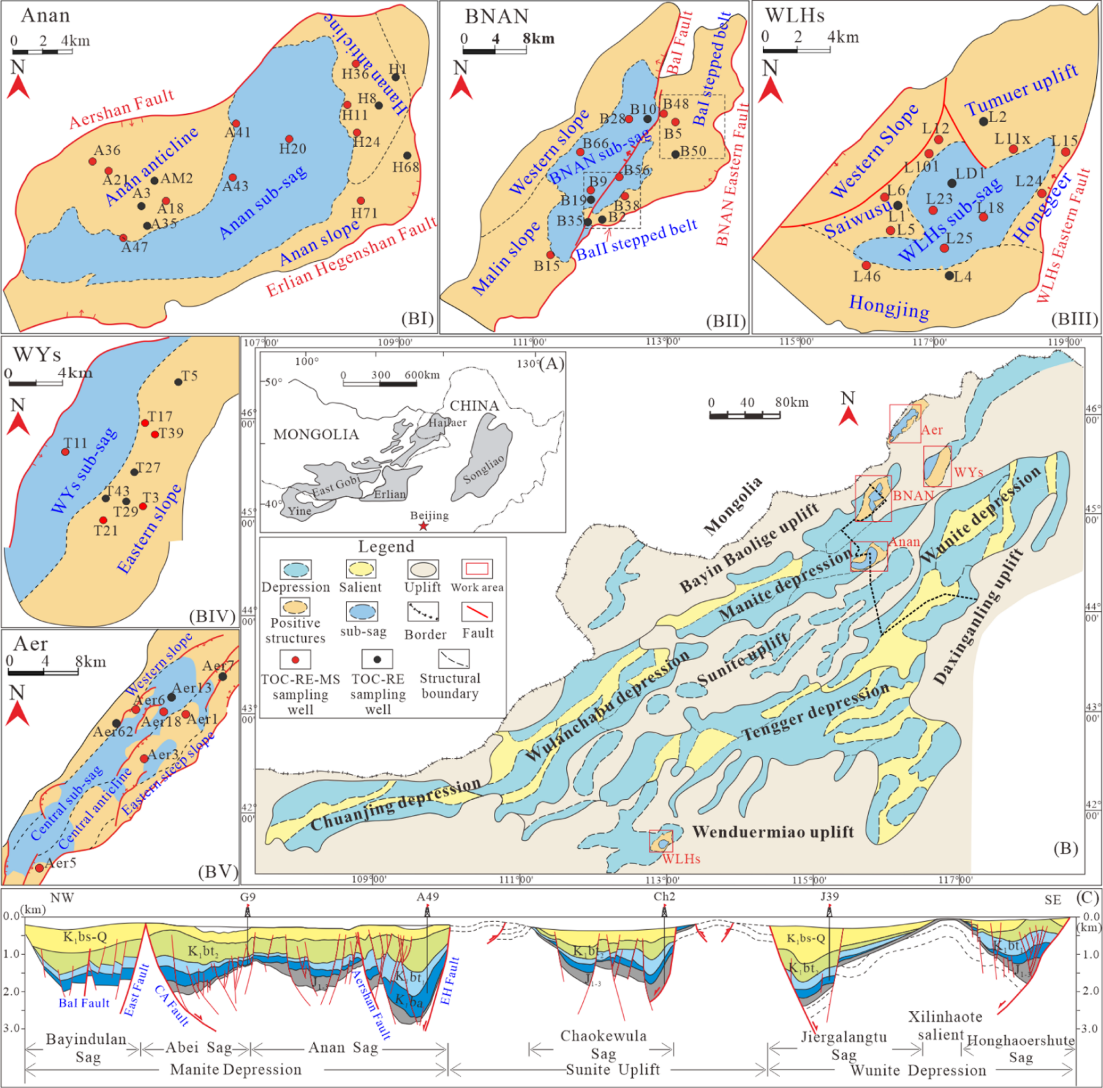
(A) Location of the Erlian Basin in Northeast China. (B) Geological map of the Erlian Basin, with locations of the Anan, southern Bayindulan (BNAN), southern Wulanhua (WLHs), southern Wuliyasitai (WYs), and the Aer sags. (BI–BV) Geological maps of the Anan, BNAN, WLHs, WYs, and Aer sags, with locations of the sampled wells. (C) Cross section showing the structure of the Erlian basin (EH Fault = Erlian Hegenshan fault; CA Fault = Chaganaobao-Arongqi Fault).

**Figure 2:**
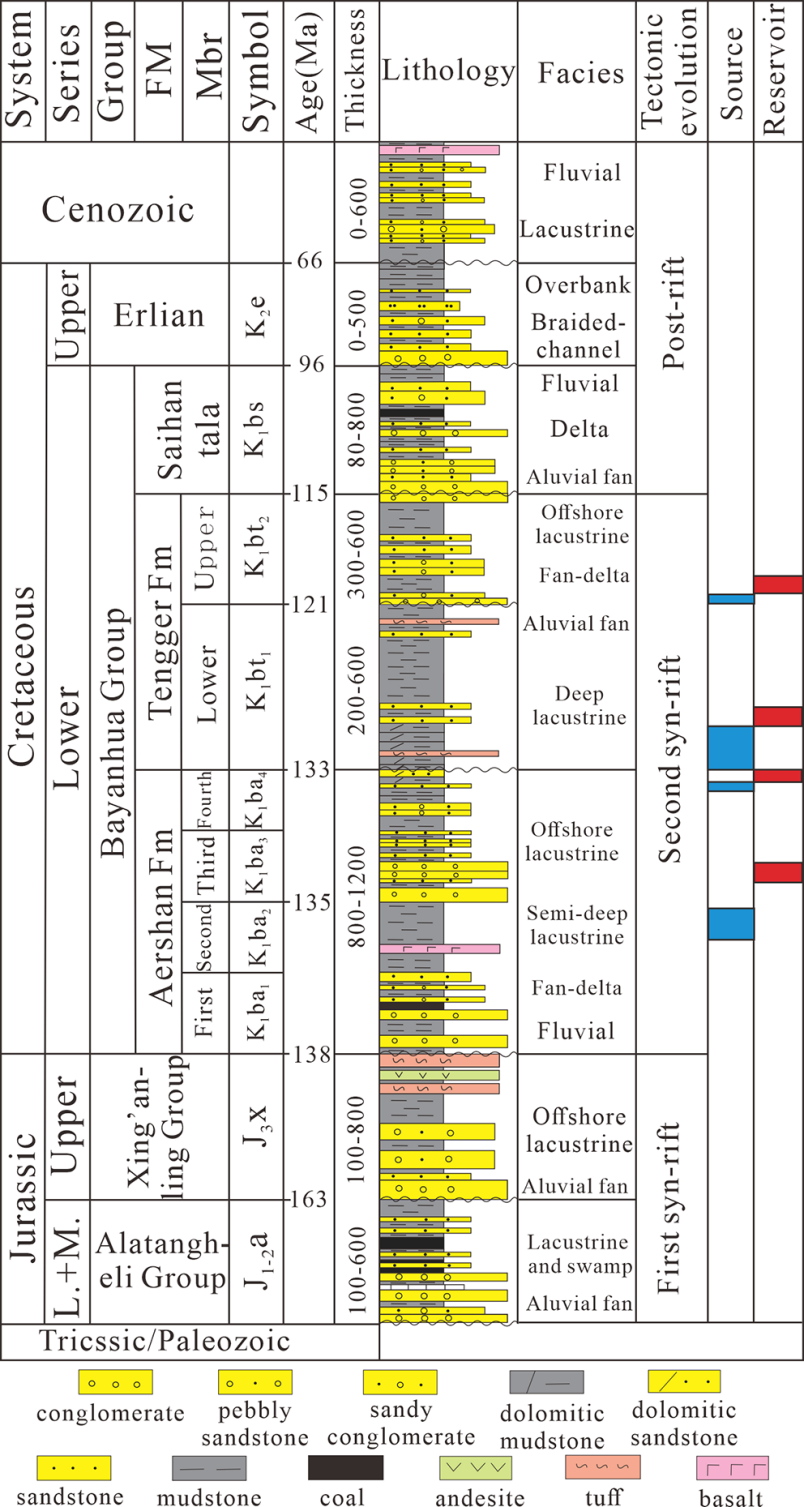
Stratigraphic section of the Erlian Basin, possible source rock, and major reservoir intervals are marked. Lithology, thickness, sedimentary facies, tectonic evolution, and depositional age are adapted with permission from Guo et al.^116,117^ Copyright 2018 Journal of Asian Earth Sciences, Copyright 2019 Marine and Petroleum Geology.

A notable tectonic magmatic event occurred in Northeast Asia in the late Mesozoic,^19^ forming a series of semi-graben-type rift basins with NE-NNE trend strikes, including the Songliao, Hailar, Erlian, and East Gobi Basins in Mongolia (Figure 1A). The Lower Cretaceous in the Erlian Basin contains multiple sets of volcanic strata (Figure 2). However, hydrothermal sediments have only been deposited in the Baiyinchagan sag of the Chuanjing Depression in the Erlian Basin. It is still uncertain whether the Lower Cretaceous strata in the other sags of the Erlian Basin were affected by hydrothermal or volcanic activity. The organic facies indicate a mappable subdivision of a stratigraphic unit with similar organic constituents.^20^ This concept is widely used to map the distributions of possible source rocks and associated petroleum. Some studies also use organic facies to assess the depositional environment of the organic matter because its lacustrine sediment composition provides important information on paleoclimate and hydrodynamic conditions over geological periods.^8,21,22^

The Erlian Basin is one of the most petroliferous basins in northeastern China, with abundant petroleum resources. The basin is characterized by multiple small sags, obvious segmentation among sags, and oil distribution controlled by sub-sags.^23^ With the improvement in the exploration of the positive structural belts, the sub-sag zone, which accounts for approximately 70% of the basin area, is considered to be the main distribution area of the remaining petroleum. Reconstruction of the paleoenvironment and genesis of high-quality source rocks are vital for assessing the hydrocarbon generation potential of source rocks in various sub-sags in the basin. Previous studies on source rocks mainly focused on a single sag, with the analysis of inorganic and bulk organic geochemical characteristics,^24−30^ the oil-source correlations,^28,31−34^ and the origin of lacustrine dolomites.^35−37^ Ding et al.^24,25,38,39^ believed that source rocks in Member 1 of the Tengger Formation (Fm) in the Erlian Basin were deposited in oxic to anoxic and fresh to brackish water conditions, with abundant terrestrial organic matter input and little aquatic organic matter input. The sources and depositional environments of the source rocks were mainly determined by differences in tectonic settings.^24^ However, Wei et al.^29,36,37^ and Luo et al.^28^ reported that the source rocks in Member 1 of the Tengger Fm and Member 4 of the Aershan Fm in the Saihan, Naoer, and Anan sags were deposited in dysoxic to anoxic and brackish to saline water environments, with biological sources mainly from bacteria and algae. The uncertainty regarding sedimentary environments and organic matter sources has significantly inhibited the prediction of source rock and the associated oil and gas distributions. Therefore, it is necessary to conduct a comparative analysis of the organic geochemistry of source rocks in multiple sags in the Erlian Basin. By integrating organic geochemical data with elemental geochemical data, our study aimed to assess the geochemical variability of the Lower Cretaceous lacustrine source rocks in the Erlian Basin and to reveal the environmental and ecological differences in different sags. In addition, the influence of the Late Mesozoic tectonic magmatic event on organic matter enrichment in the Erlian Basin can be interpreted. The results herein are significant for the reconstruction of the paleoenvironment in the Early Cretaceous and the optimization of exploration targets in the Erlian Basin.

## 2 Geological Setting

The Erlian Basin is located in northeastern China, Inner Mongolia, and covers an area of approximately 100,000 km^2^. The basin is an Early Cretaceous rift basin developed on a Hercynian folded basement^40^ and is not a unified catchment basin.^41^ It contains 65 sags and 21 salients in the five depressions of Manite, Wunit, Ulanqab, Chuanjing, Tengger, and the three uplifts of Bayinbaolige, Sunite, and Wenduermiao (Figure 1B).^42^ Each sag is relatively independent of the others for a long time but connected for a short time. The study area consists of the Aer sag, southern Wuliyasitai (WYs) sag in the Bayinbaolige uplift, southern Bayindulan (BNAN) sag, Anan sag in the Manite depression, and southern Wulanhua (WLHs) sag in the Wenduermiao uplift. In the cross section of the depositional centers, the BNAN, Anan, and WLHs sags had a graben structure (Figure 1C), whereas the WYs and Aer sags had a half-graben structure. The Anan sag could be divided into the Anan anticline, the Anan sub-sag, the Hanan anticline, and the Anan slope (Figure 1BI).^43^ The BNAN sag consists of the Western slope, the BaI stepped belt, the BaII stepped belt, the BNAN sub-sag, and the Malin slope (Figure 1BII).^44^ The WLHs sag is composed of the Hongjing uplift, the Honggeer uplift, the Saiwusu uplift, the Tumuer uplift, and the WLHs sub-sag (Figure 1BIII).^26^ The WYs sag includes the eastern slope and the WYs sub-sag (Figure 1BIV). The Aer sag comprises an eastern steep slope, a gentle western slope, a central sub-sag, and a central anticline (Figure 1BV).

The Erlian Basin has experienced three major evolutionary stages from the Jurassic to the present (Figure 2). The first syn-rift stage occurred in the Jurassic when the coal-bearing clastic rocks were deposited in the Alatanheli Group of the Lower Jurassic and the volcanic rocks formed in the Qinganling Group of the Upper Jurassic. With a thickness of 100–200 m, this set of strata only developed in some small sags and contains a range of fair–good quality source rocks.^45^ The second syn-rift stage occurred in the Early Cretaceous when the alluvial, fan deltas, and offshore-lacustrine sedimentary facies developed in the Aershan Fm, and the fan deltas, offshore-lacustrine, and semi-deep/deep lacustrine sedimentary facies developed in the Tengger Fm.^29^ This set of strata is distinguished by its considerable thickness (750–3400 m^24,28,40,46^) with abundant volcanic intercalation.^47^ The Aershan Fm can be divided into four members, with lithologic assemblages mainly composed of conglomerates, sandstone, mudstone, and some overflow facies volcanic rock in the third member.^48^ The Tengger Fm is mainly composed of clastic rocks and can be subdivided into Member 1 and Member 2. A set of special lithologic assemblages, including calcareous sandstone, pebbled sandstone, dolomitic/calcareous mudstone, and mudstone, developed in the lower part of Member 1 of the Tengger Fm.^48^ In general, strata deposited in the second syn-rift stage constitute a complete transgressive-regressive sedimentary cycle with a coarse-fine-coarse gradation in grain sizes, in which the maximum flood surface occurs in Member 1 of the Tengger Fm.^48^ The post-rift stage started from 112 Ma to the present, during which the Erlian Basin was deposited by coal-bearing clastic rocks in fluvial swamp facies in the Saihantala Fm in the Lower Cretaceous and the variegated clastic rocks in the alluvial fan and fluvial facies in the Erlian Fm in the Upper Cretaceous. It is generally believed that Member 4 of the Aershan Fm and Member 1 of the Tengger Fm are the main source rocks and reservoir layers (Figure 2).^49,50^

## 3 Samples and Methods

### 3.1 Samples

Two sample sets from Member 1 of the Tengger Fm and Member 4 of the Aershan Fm in the five sags were analyzed in this study. The first sample set consisted of 367 source rock samples (Table S1) and was only used for bulk geochemistry analyses, including total organic carbon (TOC) measurements and Rock–Eval (RE) pyrolysis. To ensure that the sampling depth covered the depth range of Member 1 in the Tengger Fm and Member 4 in the Aershan Fm, these samples were continuously collected from one or two typical wells in different structural zones of each sag. For example, of the 135 samples of the WLHs sag, 15 samples were collected from the L24X well in the Honggeer uplift, 13 samples were collected from the L4 well in the Hongjing uplift, 24 samples were collected from the L12X and L1 wells in the Saiwusu uplift, 48 samples were collected from the LD1 well in the WLHs sub-sag, and 35 samples were collected from the L15X and L2X wells in the Tumuer uplift.

The second set of samples consisted of 66 dolomitic mudstone, mudstone, and seven oil sand/oil samples (Table 1). These samples were collected from multiple wells in different structural zones with different sedimentary facies. The dolomitic mudstone samples were mainly collected from the special lithologic assemblages, whereas the mudstone samples were mainly collected from sections outside the special lithologic assemblages. All samples were subjected to Rock–Eval pyrolysis and aliphatic hydrocarbon gas chromatography–mass spectrometry (GC–MS) analyses. The seven oil sand/oil samples and 28 source rock samples were further subjected to aromatic hydrocarbon GC–MS analyses to investigate the environmental and ecological differences in the different sags (Table 2). Thirteen source rock samples in the second sample set were used for trace element and rare earth element (REE) analyses (Tables 3 and 4). In addition, trace and major elemental data of 10 samples in the WLHs and BNAN sags were provided by the PetroChina Huabei Oilfield Company. Trace elemental data of nine samples in the Anan sag were obtained from ref (37).

**Table 1:** Rock-Eval Pyrolysis Data and Gas Chromatogram Parameters of Aliphatic Fractions in the Source Rocks from Member 4 of the Aershan Fm and Member 1 of the Tengger Fm in the BNAN, WLHs, Anan, Aer, and WYs Sags in the Erlian Basin^a^

sag/zone	no.	well	strata	lithology	depth (m)	TOC (%)	*S*_2_ (mg/g)	*T*_max_ (°C)	HI (mg/g)	main peak	Pr/Ph	Pr/*n*C_17_	Ph/*n*C_18_	*n*C_21_^–^/*n*C_22_^+^	OEP
WYs	1	T3	K_1_bt_1_	MD	1399.05	1.95	7.86	439	403	*n*C_21_	0.55	0.72	1.22	1.15	1.54
WYs	2	T21	K_1_ba_4_	Oil sand	1756.2–1781					*n*C_23_	0.70	0.22	0.30	0.77	1.11
WYs	3	T17	K_1_ba_4_	MD	2360	2.70	5.62	444	208	*n*C_17_	0.87	0.17	0.22	1.10	1.02
WYs	4	T11	K_1_bt_1_	MD	1591.6	2.18	2.44	439	112	*n*C_21_	1.07	0.30	0.27	1.12	1.13
WYs	5	T17	K_1_bt_1_	MD	2065	1.99	3.43	445	172	*n*C_21_	1.38	0.25	0.17	1.33	1.17
WYs	6	T21	K_1_ba_4_	MD	2264.16	2.89	8.03	441	278	*n*C_17_	1.30	0.26	0.22	2.11	1.03
WYs	7	T39	K_1_bt_1_	MD	2045–2050	2.88	8.81	439	298	*n*C_21_	1.39	0.30	0.21	1.80	1.50
WYs	8	T39	K_1_bt_1_	MD	1963.34	4.36	16.51	439	355	*n*C_19_	1.87	0.35	0.18	2.00	1.20
WYs	9	T39	K_1_bt_1_	MD	1704–1708	2.54	5.13	439	197	*n*C_21_	2.40	0.63	0.27	1.20	1.73
WYs	10	T17	K_1_bt_1_	MD	1905.56	3.12	3.34	439	107	*n*C_19_	2.54	0.47	0.18	1.58	1.16
BaI stepped belt	11	B48	K_1_ba_4_	DMD	1184.8	3.00	19.50	434	650	*n*C_21_	0.32	0.68	1.84	1.07	1.18
BaI stepped belt	12	B5	K_1_ba_4_	DMD	1201.57	2.23	17.51	435	785	*n*C_17_	0.20	0.48	4.41	1.28	1.41
BaI stepped belt	13	B5	K_1_ba_4_	DMD	1011.11	2.45	13.55	432	553	*n*C_23_	0.24	0.76	2.29	1.54	1.15
BaII stepped belt	14	B38	K_1_ba_4_	MD	1377.72	2.78	22.92	433	824	*n*C_17_	0.48	0.54	1.42	1.88	1.23
BaII stepped belt	15	B38	K_1_ba_4_	oil sand	1376.2					*n*C_23_	0.32	1.11	3.05	1.10	1.27
Malin slope	16	B15	K_1_ba_4_	DMD	1409.5	1.90	8.52	434	441	*n*C_17_	0.28	0.65	3.19	1.57	1.38
BNAN sub-sag	17	B9	K_1_ba_4_	MD	1682.36	0.90	4.05	440	435	*n*C_23_	0.31	0.47	1.67	0.97	1.28
BNAN sub-sag	18	B28	K_1_ba_4_	DMD	1451.19	4.00	33.20	436	830	*n*C_21_	0.35	0.52	1.79	1.22	1.25
BNAN sub-sag	19	B56	K_1_ba_4_	MD	1829.5	2.78	15.26	445	549	*n*C_21_	0.41	0.41	0.93	0.72	1.14
BNAN sub-sag	20	B66	K_1_ba_4_	DMD	1570–1580	2.90	14.70	444	507	*n*C_21_	0.70	0.46	0.64	1.13	1.15
BNAN sub-sag	21	B66	K_1_ba_4_	MD	1890–1900	1.12	3.09	444	276	*n*C_21_	0.75	0.49	0.61	0.94	1.17
Hanan anticline	22	H36	K_1_bt_1_	MD	1369.38	0.94	1.11	437	118	*n*C_21_	1.39	0.47	0.37	1.28	1.23
Hanan anticline	23	H24	K_1_bt_1_	MD	1709–1714.4	0.55	1.27	446	231	*n*C_17_	1.14	0.31	0.32	1.21	1.08
Hanan anticline	24	H11	K_1_bt_1_	MD	2018.6–2023.45	1.89	11.94	445	632	*n*C_19_	0.96	0.28	0.31	2.14	1.07
Anan anticline	25	A18	K_1_ba_4_	MD	1891.5	1.60	4.80	442	300	*n*C_17_	0.80	0.30	0.51	2.20	1.03
Anan anticline	26	A36	K_1_bt_1_	DMD	1279.8–1286.2	4.87	43.27	446	889	*n*C_17_	0.54	1.08	2.61	1.78	1.19
Anan sub-sag	27	A47	K_1_ba_4_	DMD	2014.2	4.12	35.72	449	867	*n*C_17_	1.01	0.41	0.43	1.80	1.09
Anan sub-sag	28	A47	K_1_bt_1_	DMD	2034.2	3.85	30.16	444	783	*n*C_17_	1.15	0.51	0.50	1.65	1.09
Anan sub-sag	29	A47	K_1_ba_4_	DMD	2125.4	2.49	16.22	443	651	*n*C_21_	0.69	0.30	0.45	1.27	1.08
Anan sub-sag	30	A47	K_1_ba_4_	DMD	2130.1	3.03	23.15	446	764	*n*C_17_	0.81	0.22	0.29	1.86	1.06
Anan sub-sag	31	A43	K_1_bt_1_	DMD	2034.1	2.12	9.83	444	458	*n*C_19_	0.72	0.28	0.39	1.32	1.02
Anan sub-sag	32	A43	K_1_bt_1_	DMD	2058.25	1.70	6.34	449	372	*n*C_19_	0.67	0.20	0.31	1.46	1.07
Anan sub-sag	33	A43	K_1_bt_1_	DMD	2062.2	1.94	7.30	448	367	*n*C_19_	0.78	0.26	0.33	1.32	1.03
Anan sub-sag	34	A43	K_1_bt_1_	DMD	2071.56	1.13	3.75	445	326	*n*C_17_	1.15	0.15	0.14	1.21	1.01
Anan sub-sag	35	A41	K_1_bt_1_	DMD	2024.1–2028.5	0.93	3.08	454	331	*n*C_17_	0.86	0.21	0.27	2.48	1.08
Anan sub-sag	36	A41	K_1_bt_1_	DMD	2025.3	1.1	3.05	446	277	*n*C_17_	0.89	0.25	0.32	1.33	1.01
Anan sub-sag	37	H20	K_1_bt_1_	DMD	2195.3	1.53	4.41	447	288	*n*C_19_	0.92	0.24	0.25	1.07	1.03
Anan sub-sag	38	H20	K_1_bt_1_	DMD	2199.27	1.70	6.56	446	386	*n*C_21_	0.78	0.22	0.23	0.87	1.03
Anan slope	39	H71	K_1_ba_4_	MD	1789.5–1798	3.51	27.56	450	785	*n*C_17_	0.77	0.38	0.59	2.07	1.10
Anan anticline	40	A33	K_1_bt_1_	oil sand	728.5					*n*C_17_	0.31	0.33	0.44	1.6	1.3
Tumuer uplift	41	L15X	K_1_bt_1_	MD	1328–1338	2.48	8.85	438	357	*n*C_27_	0.40	1.21	3.58	0.59	3.32
Tumuer uplift	42	L15X	K_1_bt_1_	MD	1420–1430	2.38	9.89	430	416	*n*C_25_	0.23	0.84	5.17	0.58	3.61
Tumuer uplift	43	L15X	K_1_ba_4_	DMD	1705–1715	2.12	14.10	440	665	*n*C_23_	0.38	1.24	3.87	0.81	1.45
Tumuer uplift	44	L15X	K_1_ba_4_	DMD	1860–1870	2.62	15.01	444	573	*n*C_23_	0.67	0.45	0.66	0.85	1.24
Tumuer uplift	45	L15X	K_1_ba_4_	MD	1905–1915	2.17	11.37	442	524	*n*C_23_	0.45	0.54	1.20	0.95	1.31
Tumuer uplift	46	L11X	K_1_bt_1_	oil sand	1172–1199.8					*n*C_23_	0.47	0.51	0.99	0.52	1.13
Honggeer uplift	47	L24X	K_1_bt_1_	DMD	1103–1108	1.48	6.56	432	443	*n*C_23_	0.25	0.62	3.29	0.91	2.03
Honggeer uplift	48	L24X	K_1_bt_1_	DMD	1370–1380	1.38	5.88	436	426	*n*C_23_	0.30	0.54	2.18	1.07	1.95
Honggeer uplift	49	L24X	K_1_ba_4_	DMD	1774–1784	1.72	8.52	443	495	*n*C_23_	0.68	0.39	0.61	1.28	1.28
Honggeer uplift	50	L24X	K_1_ba_4_	MD	1915–1925	2.23	12.96	447	581	*n*C_21_	0.80	0.41	0.51	1.11	1.12
WLHs sub-sag	51	L18X	K_1_bt_1_	MD	1710–1720	1.68	4.02	441	239	*n*C_23_	0.67	0.51	0.78	1.10	1.56
WLHs sub-sag	52	L23X	K_1_bt_1_	MD	1738–1748	0.71	1.27	441	178	*n*C_23_	0.76	0.46	0.59	0.82	1.61
WLHs sub-sag	53	L23X	K_1_ba_4_	DMD	1925–1935	2.11	11.52	444	548	*n*C_23_	0.59	0.57	0.99	0.95	1.26
WLHs sub-sag	54	L23X	K_1_ba_4_	DMD	2040–2050	0.72	2.54	452	353	*n*C_23_	1.07	0.14	0.12	0.90	1.10
WLHs sub-sag	55	L25x	K_1_ba_4_	DMD	2335	2.33	4.61	451	198	*n*C_23_	0.80	0.11	0.13	0.83	1.07
Saiwusu uplift	56	L12X	K_1_ba_4_	MD	1890–1900	2.26	9.68	443	428	*n*C_23_	0.77	0.18	0.21		1.10
Saiwusu uplift	57	L6x	K_1_bt_1_	MD	1960–1970	1.45	4.70	445	323	*n*C_23_	0.97	0.19	0.17	0.83	1.11
Hongjing uplift	58	L46	K_1_ba_4_	DMD	1900–1910	2.43	11.03	447	453	*n*C_23_	0.88	0.28	0.31	1.08	1.18
Saiwusu uplift	59	L101X	K_1_bt_1_	oil	996–1237					*n*C_23_	0.77	0.20	0.24	0.79	1.12
Saiwusu uplift	60	L5	K_1_bt_1_	oil	1308–1764.2					*n*C_23_	0.84	0.43	0.48	0.63	1.17
Aer	61	Aer5	K_1_bt_1_	MD	1912.39	3.26	24.55	442	753	*n*C_17_	0.77	0.21	0.30	2.15	1.09
Aer	62	Aer1	K_1_bt_1_	MD	1467.4	1.87	11.54	435	617	*n*C_17_	0.50	0.25	0.65	2.33	1.53
Aer	63	Aer6	K_1_bt_1_	MD	1652.1	1.80	7.89	439	438	*n*C_17_	0.82	0.31	0.41	2.36	1.16
Aer	64	Aer1	K_1_bt_1_	MD	1798	0.83	3.11	445	375	*n*C_23_	0.54	0.30	0.57	1.06	1.12
Aer	65	Aer18	K_1_bt_1_	MD	1630–1640	1.17	5.44	430	465	*n*C_21_	0.82	0.49	0.55	0.92	1.13
Aer	66	Aer18	K_1_bt_1_	MD	1805–1815	2.30	12.32	433	536	*n*C_21_	0.74	0.50	0.63	0.69	1.10
Aer	67	Aer18	K_1_bt_1_	MD	1965–1975	0.80	2.51	440	315	*n*C_21_	0.94	0.40	0.37	0.61	1.13
Aer	68	Aer18	K_1_ba_4_	MD	2096–2106	0.63	0.96	445	152	*n*C_21_	1.09	0.39	0.32	0.61	1.10
Aer	69	Aer18	K_1_ba_4_	MD	2305–2315	0.51	0.66	430	130	*n*C_19_	1.02	0.32	0.31	0.83	1.05
Aer	70	Aer18	K_1_ba_4_	MD	2474–2484	0.73	1.60	435	220	*n*C_21_	0.81	0.36	0.41	0.76	1.09
Aer	71	Aer18	K_1_ba_4_	MD	2597–2607	0.52	0.62	435	120	*n*C_21_	1.17	0.32	0.24	0.78	1.05
Aer	72	Aer3	K_1_bt_1_	oil	1783.4–1911.2					*n*C_23_	0.62	0.32	0.49	0.52	1.12
Aer	73	Aer52	K_1_bt_1_	oil	1245.4–1825.4					*n*C_21_	0.63	0.54	0.74	0.64	1.22

a MD = mudstone, DMD = dolomitic mudstone.

**Table 2:** Mass Chromatogram Parameters of Aliphatic and Aromatic Fractions in the Source Rocks from Member 4 of the Aershan Fm and Member 1 of the Tengger Fm in the BNAN, WLHs, Anan, Aer, and WYs Sags in the Erlian Basin^a^

sag/zone	no.	well	strata	lithology	depth (m)	*A*	*B*	*C*	*D*	*E*	*F*	*G*	*H*	*I*	*J*	*K*	*L*	*M*
WYs	1	T3	K_1_bt_1_	MD	1399.05	0.05	0.23	0.23	0.28	0.26	0.01	0.34		nd				
WYs	2	T21	K_1_ba_4_	oil sand	1756.2–1781	0.06	0.55	0.52	0.41	0.18	0.04	0.45	0.68	nd	0.09	0.11	nd	0.68
WYs	3	T17	K_1_ba_4_	MD	2360	0.17	0.39	0.48	0.64	0.49	0.25	0.49	0.39	nd	0.06	0.05	nd	0.86
WYs	4	T11	K_1_bt_1_	MD	1591.6	0.34	0.41	0.5	0.29	0.25	0.07	0.78	0.63	nd	0.35	0.03	nd	0.57
WYs	5	T17	K_1_bt_1_	MD	2065	0.03	0.53	0.44	0.38	0.21	0.03	1.41	1.36	nd	0.21	0.03	nd	0.69
BaI stepped belt	11	B48	K_1_ba_4_	DMD	1184.8	0.25	0.25	0.34	0.45	0.54	0.08	0.09		0.34	5.39	0.10	0.82	0.15
BaI stepped belt	12	B5	K_1_ba_4_	DMD	1201.57	0.21	0.16	0.11	0.42	0.91	0.03	0.14			10	0.05	0.76	0.09
BaI stepped belt	13	B5	K_1_ba_4_	DMD	1011.11	0.28	0.21	0.26	0.46	0.65	0.05	0.06		0.35	7.12	0.08	0.83	0.14
BaII stepped belt	14	B38	K_1_ba_4_	MD	1377.72	0.43	0.23	0.13	0.57	1.56	0.12	0.13		0.32	1.91	0.08	0.78	0.24
BaII stepped belt	15	B38	K_1_ba_4_	oil sand	1376.2	0.32	0.24	0.24	0.42	0.42	0.04	0.07		0.39	4.29	0.09	0.89	0.11
Malin slope	16	B15	K_1_ba_4_	DMD	1409.5	0.02	0.22	0.06	0.21	0.37	0.01	0.20			7.19	0.05	0.76	0.14
BNAN sub-sag	17	B9	K_1_ba_4_	MD	1682.36	0.21	0.31	0.36	0.41	0.21	0.02	0.10		0.38	2.13	0.06	0.65	0.11
BNAN sub-sag	18	B28	K_1_ba_4_	DMD	1451.19	0.75	0.19	0.31	0.54	1.70	0.08	0.08		1.00	2.03	0.11	0.74	0.18
BNAN sub-sag	19	B56	K_1_ba_4_	MD	1829.5	0.41	0.35	0.46	0.35	0.18	0.04	0.13	0.59	0.70	0.67	0.04	1.00	0.17
BNAN sub-sag	20	B66	K_1_ba_4_	DMD	1570–1580	0.06	0.3	0.39	0.33	0.21	0.02	0.29	0.69	0.18				
BNAN sub-sag	21	B66	K_1_ba_4_	MD	1890–1900	0.08	0.24	0.21	0.29	0.41	0.02	0.67	0.68	0.12				
Hanan anticline	22	H36	K_1_bt_1_	MD	1369.38	0.07	0.26	0.22	0.28	0.11	0.11	0.45	0.80		1.10	0.03	0.76	0.12
Hanan anticline	23	H24	K_1_bt_1_	MD	1709–1714.4	0.07	0.48	0.37	0.38	0.07	0.09	0.45						
Hanan anticline	24	H11	K_1_bt_1_	MD	2018.6–2023.45	0.94	0.48	0.38	0.29	0.20	0.16	0.20						
Anan anticline	25	A18	K_1_ba_4_	MD	1891.5	0.23	0.28	0.43	0.50	0.66	0.31	0.56	0.32	0.01	0.06	0.03	nd	0.71
Anan anticline	26	A36	K_1_bt_1_	DMD	1279.8–1286.2	0.66	0.21	0.31	0.37	0.71	0.12	0.04						
Anan sub-sag	27	A47	K_1_ba_4_	DMD	2014.2	0.32	0.45	0.42	0.32	0.08	0.12	0.12	0.33	0.04	0.03	0.09	nd	0.64
Anan sub-sag	28	A47	K_1_bt_1_	DMD	2034.2	0.16	0.43	0.39	0.36	0.07	0.13	0.12	0.35	0.03	0.03	0.10	nd	0.52
Anan sub-sag	29	A47	K_1_ba_4_	DMD	2125.4	0.2	0.42	0.44	0.41	0.17	0.07	0.13	0.53	0.16	0.02	0.03	nd	0.39
Anan sub-sag	30	A47	K_1_ba_4_	DMD	2130.1	0.35	0.39	0.43	0.44	0.26	0.11	0.16	0.45	0.06	0.01	0.03	nd	0.51
Anan sub-sag	31	A43	K_1_bt_1_	DMD	2034.1	0.41	0.47	0.43	0.40	0.48	0.20	0.10		0.02				
Anan sub-sag	32	A43	K_1_bt_1_	DMD	2058.25	0.56	0.53	0.41	0.47	0.6	0.35	0.13		0.04				
Anan sub-sag	33	A43	K_1_bt_1_	DMD	2062.2	0.92	0.47	0.37	0.36	0.64	0.35	0.14		0.05				
Anan sub-sag	34	A43	K_1_bt_1_	DMD	2071.56	0.17	0.5	0.5	0.47	0.27	0.19	0.30						
Anan sub-sag	35	A41	K_1_bt_1_	DMD	2024.1–2028.5	0.58	0.49	0.39	0.40	0.50	0.51	0.20						
Anan sub-sag	36	A41	K_1_bt_1_	DMD	2025.3	0.2	0.542	0.46	0.52	0.34	0.23	0.45	0.38	0.02	0.02	0.03	1.0	0.80
Anan sub-sag	37	H20	K_1_bt_1_	DMD	2195.3	0.22	0.49	0.44	0.41	0.86	0.95	0.10	0.15	0.03				
Anan sub-sag	38	H20	K_1_bt_1_	DMD	2199.27	0.28	0.49	0.45	0.41	1.24	1.59	0.09	0.12	0.02				
Anan slope	39	H71	K_1_ba_4_	MD	1789.5–1798	0.33	0.35	0.45	0.39	0.21	0.13	0.14						
Anan anticline	40	A33	K_1_bt_1_	oil sand	728.5	0.2	0.33	0.30	0.39	0.32	0.13	0.10	0.80	0.01	0.23	0.03	0.87	0.36
Tumuer uplift	41	L15X	K_1_bt_1_	MD	1328–1338	0.38	0.25	0.1	0.33	0.51	0.01	0.84	0.56	0.37	4.05	0.05	0.88	0.11
Tumuer uplift	42	L15X	K_1_bt_1_	MD	1420–1430	0.53	0.25	0.09	0.39	0.80	0.01	0.89	0.58	0.40				
Tumuer uplift	43	L15X	K_1_ba_4_	DMD	1705–1715	0.32	0.27	0.3	0.60	1.64	0.02	0.89	1.08	0.48				
Tumuer uplift	44	L15X	K_1_ba_4_	DMD	1860–1870	0.08	0.22	0.23	0.26	0.33	0.02	0.43	0.93	0.13	2.39	0.05	0.82	0.30
Tumuer uplift	45	L15X	K_1_ba_4_	MD	1905–1915	0.24	0.24	0.21	0.42	1.08	0.03	0.65	0.80	0.27				
Tumuer uplift	46	L11X	K_1_bt_1_	oil sand	1172–1199.8	0.27	0.28	0.31	0.43	0.50	0.02	0.48	0.66	0.24	4.0	0.03	0.89	0.27
Honggeer uplift	47	L24X	K_1_bt_1_	DMD	1103–1108	0.39	0.23	0.14	0.48	1.25	0.01	0.91	1.02	0.40				
Honggeer uplift	48	L24X	K_1_bt_1_	DMD	1370–1380	0.35	0.22	0.13	0.42	1.17	0.01	0.61	0.85	0.27				
Honggeer uplift	49	L24X	K_1_ba_4_	DMD	1774–1784	0.1	0.2	0.18	0.28	0.42	0.02	0.51	0.77	0.16	1.48	0.04	0.89	0.40
Honggeer uplift	50	L24X	K_1_ba_4_	MD	1915–1925	0.06	0.21	0.23	0.21	0.27	0.02	0.47	0.71	0.12				
WLHs sub-sag	51	L18X	K_1_bt_1_	MD	1710–1720	0.27	0.27	0.07	0.22	0.52	0.03	0.57	0.58	0.18				
WLHs sub-sag	52	L23X	K_1_bt_1_	MD	1738–1748	0.24	0.21	0.11	0.22	0.41	0.03	0.72	0.39	0.06				
WLHs sub-sag	53	L23X	K_1_ba_4_	DMD	1925–1935	0.47	0.41	0.33	0.30	0.34	0.04	0.49	0.36	0.15	1.31	0.02	0.86	0.32
WLHs sub-sag	54	L23X	K_1_ba_4_	DMD	2040–2050	0.12	0.41	0.4	0.29	0.12	0.06	0.70	0.73	0.04				
WLHs sub-sag	55	L25x	K_1_ba_4_	DMD	2335	0.28	0.5	0.52	0.18	0.16	0.06	1.49	1.65	0.06	0.047	0.03	nd	0.80
Saiwusu uplift	56	L12X	K_1_ba_4_	MD	1890–1900	0.08	0.44	0.46	0.32	0.15	0.03	0.52	0.77		0.28	0.05	1.00	0.33
Saiwusu uplift	57	L6x	K_1_bt_1_	MD	1960–1970	0.06	0.43	0.51	0.33	0.17	0.03	0.63	0.75	0.06	0.16	0.03	1.00	0.65
Hongjing uplift	58	L46	K_1_ba_4_	DMD	1900–1910	0.06	0.35	0.35	0.19	0.16	0.03	0.69	0.72	0.05				
Saiwusu uplift	59	L101X	K_1_bt_1_	oil	996–1237	0.07	0.42	0.53	0.34	0.18	0.04	0.44	0.70	0.03	0.20	0.05	1.00	0.40
Saiwusu uplift	60	L5	K_1_bt_1_	oil	1308–1764.2	0.06	0.29	0.4	0.29	0.24	0.02	0.34	0.92	0.06	0.58	0.02	0.81	0.30
Aer	61	Aer5	K_1_bt_1_	MD	1912.39	0.08	0.48	0.45	0.32	0.21	0.12	0.22						
Aer	62	Aer1	K_1_bt_1_	MD	1467.4	0.21	0.22	0.2	0.43	0.80	0.04	0.21	0.50	0.23	0.06	0.01	1.00	0.59
Aer	63	Aer6	K_1_bt_1_	MD	1652.1	0.05	0.36	0.47	0.30	0.62	0.04	0.30						
Aer	64	Aer1	K_1_bt_1_	MD	1798	0.05	0.37	0.38	0.44	0.24	0.04	0.12						
Aer	65	Aer18	K_1_bt_1_	MD	1630–1640	0.07	0.25	0.38	0.42	0.28	0.02	0.49	1.27	0.11				
Aer	66	Aer18	K_1_bt_1_	MD	1805–1815	0.12	0.38	0.5	0.30	0.22	0.03	0.40	1.05	0.19				
Aer	67	Aer18	K_1_bt_1_	MD	1965–1975	0.05	0.42	0.51	0.37	0.24	0.02	0.82	1.73	0.07				
Aer	68	Aer18	K_1_ba_4_	MD	2096–2106	0.06	0.43	0.48	0.38	0.26	0.03	0.72	1.45					
Aer	69	Aer18	K_1_ba_4_	MD	2305–2315	0.07	0.3	0.38	0.50	0.35	0.03	1.28	1.33					
Aer	70	Aer18	K_1_ba_4_	MD	2474–2484	0.11	0.28	0.37	0.41	0.35	0.03	0.78	1.31					
Aer	71	Aer18	K_1_ba_4_	MD	2597–2607	0.07	0.29	0.37	0.51	0.33	0.04	1.04	0.92					
Aer	72	Aer3	K_1_bt_1_	oil	1783.4–1911.2	0.12	0.47	0.46	0.27	0.16	0.04	0.32	0.55	0.18	0.25	0.02	1.00	0.82
Aer	73	Aer52	K_1_bt_1_	oil	1245.4–1825.4	0.07	0.36	0.41	0.26	0.18	0.03	0.43	0.84	0.18	0.53	0.03	1.00	0.54

a *A*: Gam/C_30_H = gammacerane/C_30_ 17α-hopane; *B*: C_29_ββ/(αα + ββ) = C_29_ (αββ20R + αββ20S) steranes/C_29_ (ααα20S + αββ20R + αββ20S + ααα20R) steranes; *C*: C_29_αα20S/(20S + 20R) = C_29_ααα20S steranes/C_29_ααα (20S + 20R) steranes; *D*: C_28_/C_29_ST = C_28_(ααα20S + αββ20R + αββ20S + ααα20R) steranes/C_29_(ααα20S + αββ20R + αββ20S + ααα20R) steranes; *E*: S/H = ∑C_27_–C_29_ (ααα20S + αββ20R + αββ20S + ααα20R) steranes/∑C_29_–C_35_ 17α-hopane; *F*: C_23_TT/C_30_H = C_23_ tricyclic terpane/C_30_ 17α-hopane; *G*: C_19_/C_23_TT = C_19_ tricyclic terpane/C_23_ tricyclic terpane; *H*: C_24_Tet/C_23_TT = C_24_ tetracyclic terpane/C_23_ tricyclic terpane; *I*: β-carotane/*n*C_max_ = β-carotane/predominant *n*-alkane in total ion chromatogram; *J*: Ret/9-MP = retene/9-methylphenanthrene; *K*: DBT/P = dibenzothiophene/phenanthrene; *L*: MTTC ratio = 5,7,8-trimethyl-MTTC (α-MTTC)/total MTTCs; *M*: C_20_/(C_20_ + C_27_)TAS = C_20_TAS/(C_20_TAS + C_27_, 20R TAS); nd = not detected.

**Table 3:** Trace, Major Elemental Data, and Sr/Ba Ratio in the Source Rocks from Member 4 of the Aershan Fm and Member 1 of the Tengger Fm in the BNAN, WLHs, Anan, Aer, and WYs Sags in the Erlian Basin

sag/zone	no.	well	strata	depth (m)	lithology	Sr (ppm)	Ba (ppm)	V (ppm)	Cr (ppm)	Co (ppm)	Ni (ppm)	Cu (ppm)	Zn (ppm)	Mn (ppm)	Fe (%)	Sr/Ba	Ni/Co	V/(V + Ni)
WYs	1	T11	K_1_bt_1_	1594.49	MD	198	542	121	47.4	13.5	23.2	35.1	154	414	1.35	0.37	1.72	0.84
WYs	2	T3	K_1_bt_1_	1401.25	MD	183	451	120	63.6	23.2	64.2	39.4	261	524	1.2	0.41	2.77	0.65
BNAN sub-sag	3	B9	K_1_ba_4_	1683.36	MD	299	534	64.7	30.8	8.71	17.6	17.1	80.2	712	4.11	0.56	2.02	0.79
Malin slope	4	B15	K_1_ba_4_	1407.3	DMD	172	564	71.7	61.8	13.7	56.6	27.6	89.5	631	2.83	0.30	4.13	0.56
BaI stepped belt	5	B1	K_1_ba_4_	1212.83	DMD	365	398	70.6	37.5	9.36	14.5	17.4	70.6	803	5.41	0.92	1.55	0.83
BaII stepped belt	6	B2	K_1_bt_1_	978.28	MD	221	424	25.6	12.8	4.09	10.7	9.01	26.8	512	4.26	0.52	2.62	0.71
BaI stepped belt	7	B5	K_1_ba_4_	880	MD	373	371	76.8	48.3	11.1	38.1	25	93	905	3.55	1.01	3.43	0.67
BaI stepped belt	8	B5	K_1_ba_4_	942.7	MD	646	330	53.5	19.1	5.34	17.4	17.3	36.3	1123	4.81	1.96	3.26	0.75
BaI stepped belt	9	B5	K_1_ba_4_	1010	DMD	141	448	69.9	40.7	12	28.6	27.1	99.2	1242	6.66	0.31	2.38	0.71
BaI stepped belt	10	B5	K_1_ba_4_	1205.7	DMD	284	385	77.3	48.9	7.03	14.2	32.7	100	1315	5.02	0.74	2.02	0.84
Hanan anticline	11	H36	K_1_bt_1_	1362.9	MD	160	508	46.1	44.7	9.85	30.4	13.4	65.6	71.5	1.78	0.31	3.09	0.6
Anan sub-sag	12	H20	K_1_bt_1_	1752.96	MD	152	428	137	89.8	9.27	27	32.7	67.1	86.2	1.88	0.36	2.91	0.84
Anan sub-sag	13	H20	K_1_bt_1_	1754.41	MD	158	477	116	83.1	22.3	90.3	34.2	168	209.7	2.15	0.33	4.05	0.56
Anan anticline	14	A23	K_1_bt_1_		DMD	438	356	103	60.4	16.2	62.9	21.8	86.0	703	3.45	1.23	3.88	0.62
Anan anticline	15	A3	K_1_bt_1_		DMD	484	438	119	69.2	18.7	57.8	39.7	129.8	751	3.37	1.11	3.09	0.67
Anan anticline	16	H23-1	K_1_bt_1_		DMD	450	405	51.6	32.1	8.2	115	25.3	94.6	636	4.07	1.11	13.9	0.31
Anan anticline	17	H84	K_1_bt_1_		DMD	302	240	52.1	32.4	8.3	50.5	14.8	68.9	823	4.12	1.26	6.07	0.51
Anan sub-sag	18	A43-1	K_1_bt_1_		DMD	459	472	98.6	58.0	15.5	30.8	28.2	101.7	942	3.14	0.97	1.98	0.76
Hanan anticline	19	H16	K_1_bt_1_		MD	451	463	82.8	49.3	13.1	67.7	23.3	89.7	218	0.85	0.97	5.17	0.55
Hanan anticline	20	H23-2	K_1_bt_1_		MD	396	404	72.8	43.8	11.5	78.5	21.2	84.6	429	1.91	0.98	6.81	0.48
Hanan anticline	21	H46	K_1_bt_1_		DMD	377	301	86.7	51.4	13.7	44.1	26.6	97.8	752	2.35	1.25	3.22	0.66
Anan sub-sag	22	A43-2	K_1_bt_1_		DMD	711	432	46.8	29.5	7.5	64.4	18.7	78.5	918	2.72	1.65	8.58	0.42
WLHs sub-sag	23	LD1	K_1_bt_1_	1609.37	MD	195	544	163	90	25.5	38	35	135	265	3.52	0.36	1.49	0.81
WLHs sub-sag	24	LD1	K_1_bt_1_	1690	MD	172	381	150	114	23.5	41	38	116	470	4.74	0.45	1.74	0.79
WLHs sub-sag	25	LD1	K_1_bt_1_	1762.32	MD	207	119	181	133	28.3	138	46	258	424	5.39	1.74	4.88	0.57
WLHs sub-sag	26	LD1	K_1_bt_1_	1883.38	DMD	281	750	173	119	27.1	77	50	132	358	4.85	0.37	2.84	0.69
WLHs sub-sag	27	LD1	K_1_ba_4_	2035	MD	243	384	189	126	29.5	68	50	152	1434	5.61	0.63	2.30	0.74
WLHs sub-sag	28	LD1	K_1_ba_4_	2063.55	MD	219	253	42	69	6.8	71	57	165	756	7.37	0.87	10.5	0.37
WLHs sub-sag	29	LD1	K_1_ba_4_	2125	MD	177	322	159	125	16.2	63	51	134	931	5.35	0.55	3.88	0.72
WLHs sub-sag	30	LD1	K_1_ba_4_	2226	MD	182	411	165	112	18.7	56	44	168	1125	4.85	0.44	2.99	0.75
WLHs sub-sag	31	LD1	K_1_ba_4_	2350	DMD	168	261	173	119	8.2	64	53	139	1511	5.5	0.64	7.8	0.73
Aer	32	Aer3	K_1_bt_1_	1798.61	MD	212	497	75.8	39.1	10.1	24.1	30.3	113	86	0.8	0.43	2.39	0.76
Aer	33	Aer1	K_1_bt_1_	1468.2	MD	139	412	37.9	19.2	19.7	37.5	12	96	86.5	0.6	0.34	1.9	0.5
Aer	34	Aer5	K_1_bt_1_	1911.39	MD	182	540	52.2	28.3	10.3	18.7	17.3	89.9	51.44444	1.1	0.34	1.82	0.74
Aer	35	Aer6	K_1_bt_1_	1650.6	MD	107	467	64.5	39.5	10.5	21.4	25.9	75.3	82.57143	0.9	0.23	2.04	0.75

The thickness of the dolomitic mudstone was determined based on the lithological data of 108 wells in the five sags of the Erlian Basin, which were provided by PetroChina Huabei Oilfield Company.

### 3.2 TOC Measurement and Rock–Eval Pyrolysis

The samples were crushed to 100 mesh for TOC measurements in a LECO CS-744 analyzer. The crushed samples were first pretreated with 5% hydrochloric acid and then burned in a high-temperature oxygen flow to generate oxides. The TOC and total sulfur contents were calculated using the generated carbon dioxide and sulfur dioxide with standard deviations within 0.1%. Rock–Eval pyrolysis was performed using an OGE-II oil and gas evaluation workstation. The crushed samples were initially heated to 300 °C in an inert atmosphere, held at the temperature of 300 °C for 3 min to generate free hydrocarbon (*S*_1_), and then heated to 600 °C at a rate of 25 °C/min to generate the pyrolysis hydrocarbon (*S*_2_). The hydrogen index (HI) was calculated by normalizing *S*_2_ to the TOC. *T*_max_ was defined as the temperature corresponding to the maximum generation rate of hydrocarbons in the kerogen cracking process. The relative double difference of *S*_2_ was less than 10%, and the deviation in *T*_max_ was less than 2 °C.

### 3.3 Gas Chromatography and GC-Mass Spectrometry

The selected core samples were rinsed with deionized water to remove the surface contaminants and then powdered to 100 mesh for GC and GC-MS analysis. The powders were extracted using chloroform in a Soxhlet apparatus for 48 h, and the extractable maltene was separated by column chromatography on silica gel into aliphatic hydrocarbons (elution with *n*-hexane), aromatic hydrocarbons (elution with *n*-hexane/dichloromethane,1:2, v/v), and polars following the procedures of the industry standard (SY/T 5119-2008).

GC analysis of the aliphatic fractions was performed using a Model HP6890N chromatography instrument equipped with an HP-5MS fused silica capillary column (60 m × 0.25 mm × 0.25 mm). The oven temperature was initially held at 50 °C for 1 min, increased to 120 °C at 20 °C/min, increased from 120 to 310 °C at 3 °C/min, and held at 310 °C for 15 min. Helium was used as the carrier gas at a linear rate of 1 mL/min. The distributions of isoprenoids and *n*-alkanes were identified in the aliphatic fraction gas chromatograms, and the relevant ratios were calculated based on peak area integrations.

GC-MS analysis of the aliphatic and aromatic fractions was performed using a Model HP6890N GC/5975IMSD instrument equipped with an HP-5MS fused silica capillary column (30 m × 0.25 mm × 0.25 mm). The GC oven temperature was initially held at 50 °C for 1 min, programmed to 120 °C at 20 °C/min, then to 310 °C at 3 °C/min, and held at 310 °C for 15 min. Helium was used as the carrier gas, and the samples were routinely analyzed in full scan mode (*m/z* 50–580). Terpenes and steranes were identified using *m*/*z* = 191 and *m*/*z* = 217 mass spectrometers of the aliphatic fractions. Typical alkylnaphthalene (*m*/*z* = 128, 170, 184, 198), alkylphenanthrene (*m*/*z* = 178, 192, 234), dibenzothiophene (DBT) (*m*/*z* = 184, 198, 212), methyltrimethyltridecyl chromans (*m*/*z* = 121, 135, 149), and triarylsterane (*m*/*z* = 231) were identified by MS of the aromatic fractions.

### 3.4 Element Analysis

The samples used for elemental analysis were crushed to 100 mesh. Approximately 50 mg of the powder was first digested with hydrofluoric acid (30% HF) and itric acid (68% HNO_3_) at 190 °C for 24 h in Teflon cups. After the samples were dissolved, the solution was cooled to room temperature and combined with 0.5 mL of perchloric acid (HClO_4_) to oxidize the organic matter. The solution was then dried on a hot plate at 150 °C. After cooling, 2 mL of HNO_3_ was added to the residue and dried again at 150 °C for 12 h. Finally, the resulting solution was transferred into a 50 mL volumetric flask and diluted with deionized water to 50 mL for the content measurement of the major, trace, and REEs. Major element analyses were conducted with the ICPS-1000IV using inductively coupled plasma-atomic emission spectrometry (ICP-AES) with an analytical precision of ±1% for sample concentrations >1.0% and ±10% for sample concentrations <1.0%. The contents of trace and REEs were measured using the Thermo X Series II using inductively coupled plasma-mass spectrometry, with relative standard deviations <5% for trace elements and <10% for REEs.

## 4 Results

### 4.1 Dolomitic Mudstone in the Source Rocks

**Figure 3:**
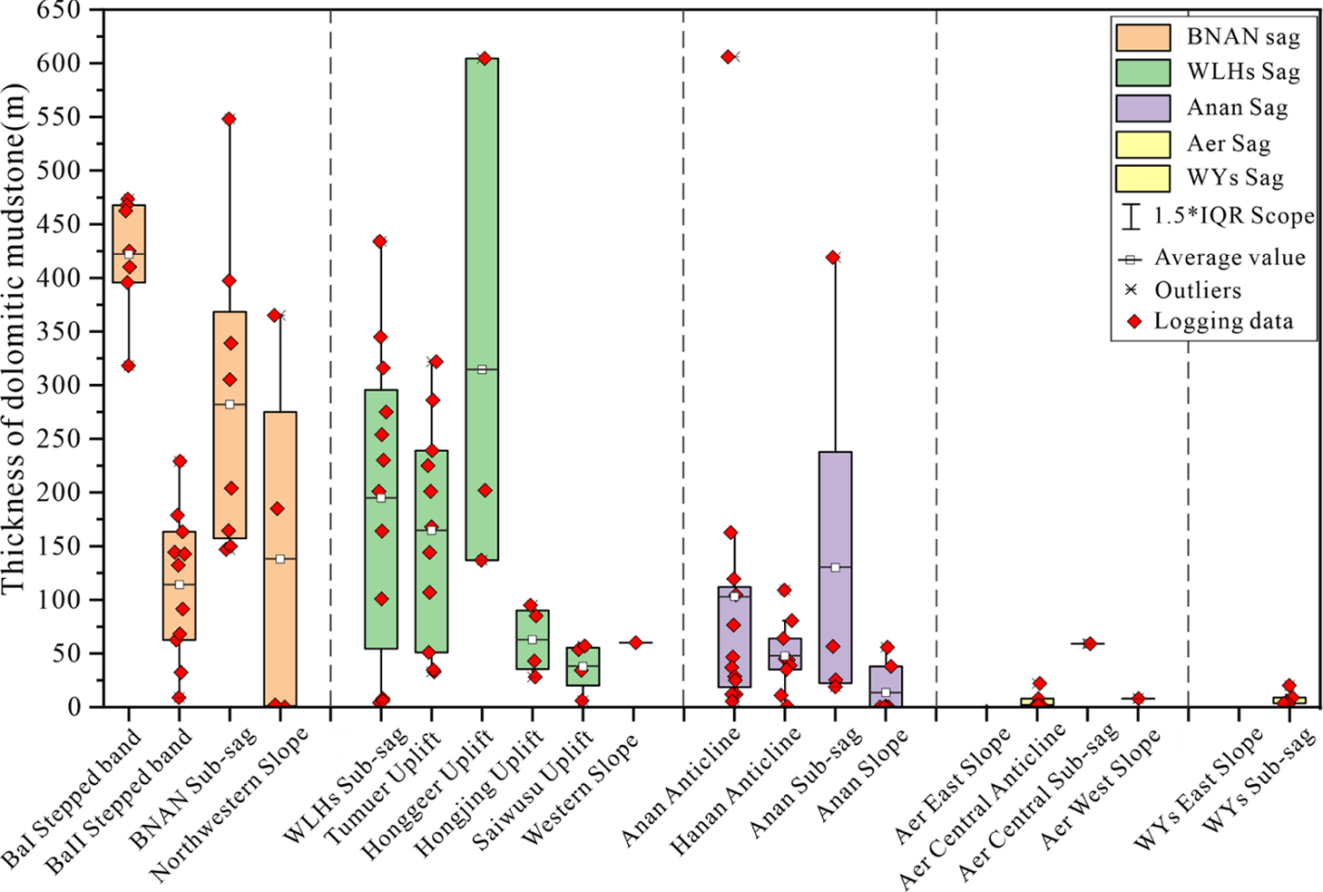
Thickness boxplot of the dolomitic mudstone in Member 4 of the Aershan Fm and Member 1 of the Tengger Fm in different structural zones of the BNAN, WLHs, Anan, Aer, and WYs sags in the Erlian Basin.

The thickness of the dolomitic mudstone in Member 1 of the Tengger Fm and Member 4 of the Aershan Fm in different sags varied greatly in the Erlian Basin (Figure 3). The BNAN sag had the thickest dolomitic mudstone (average of 233 m), followed by the WLHs sag (average of 172.9 m) and the Anan sag (average 74 m), whereas the Aer and WYs sags seldom contained dolomitic mudstone in Member 1 of the Tengger Fm and Member 4 of the Aershan Fm. In the BNAN sag, the BaI stepped belt has the thickest dolomitic mudstone (average 422 m), followed by the BNAN sub-sag (average 282 m), with the BaII stepped belt and western slope showing the thinnest dolomitic mudstone (average 120.4 m). In the WLHs sag, the Honggeer uplift had the thickest dolomitic mudstone (average 314 m), followed by the Tumuer uplift and WLHs sub-sag (average 180 m), while the Hongjing uplift, Saiwusu uplift, and WLHs western slope have the thinnest dolomitic mudstone (average 50 m). In the Anan sag, the average thickness of dolomitic mudstone followed the order of the Anan sub-sag (average 130 m) > Anan anticline (average 83.9 m) > Hanan anticline (average 47.6 m) > Anan slope (average 31 m).

### 4.2 Bulk Organic Geochemical Characteristics

**Figure 4:**
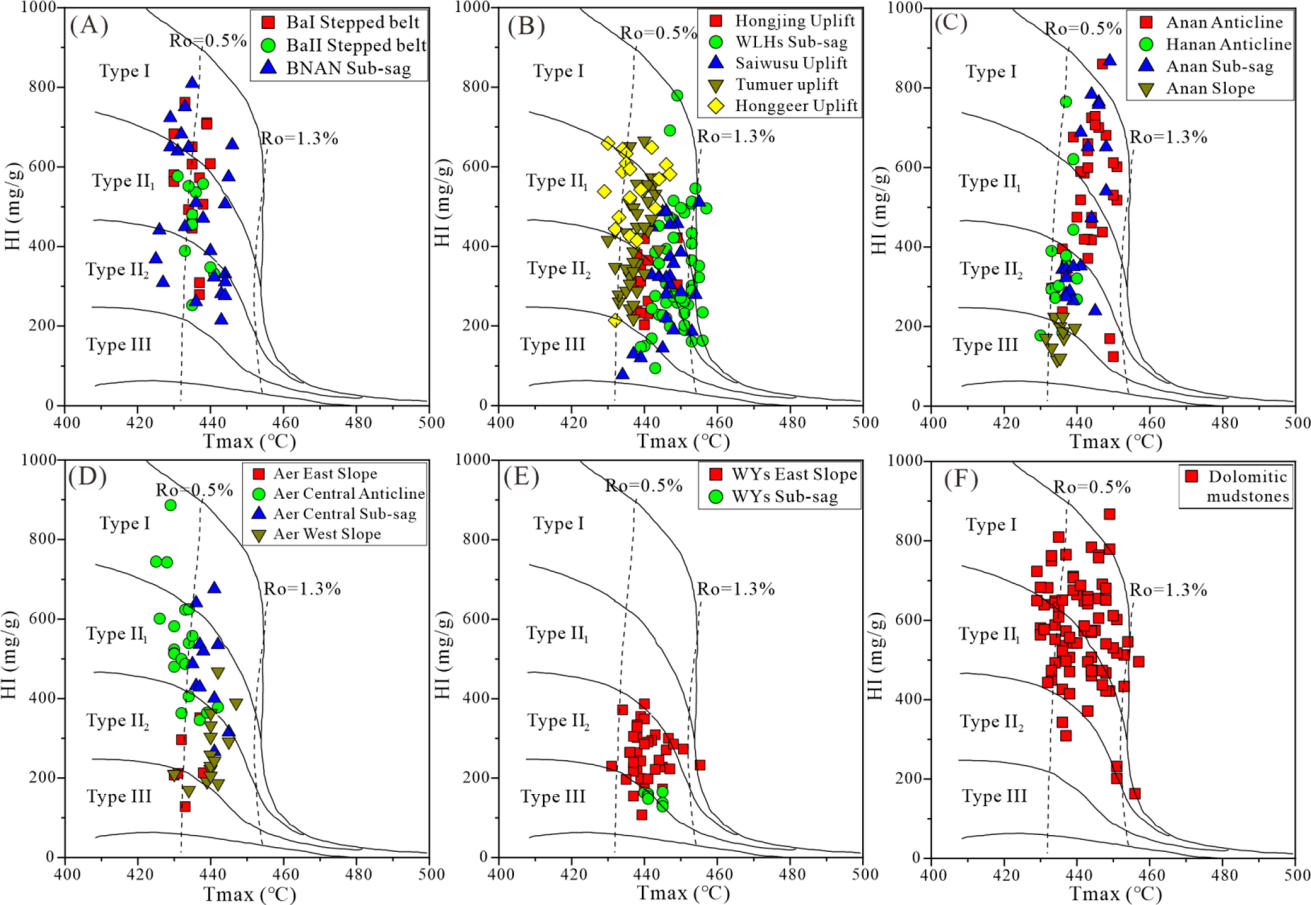
Plot of the HI vs *T*_max_ outlining kerogen types of source rocks (A–E) and dolomitic mudstone (F) in Member 4 of the Aershan Fm and Member 1 of the Tengger Fm in different structural zones of the BNAN (A), WLHs (B), Anan (C), Aer (D), and WYs (E) sags in the Erlian Basin.

The HI and *T*_max_ values of the source rocks in Member 1 of the Tengger Fm and Member 4 of the Aershan Fm in the five sags are listed in Table S1 and shown in Figure 4. The HI of the source rock samples ranged between 214 and 809 mg/g (average 504 mg/g) in the BNAN sag, 142 and 779 mg/g (average 379 mg/g) in the WLHs sag, 116 and 867 mg/g (average 432 mg/g) in the Anan sag, and 128 and 886 mg/g (average 420 mg/g) in the Aer sag, suggesting the I–II_2_ kerogen type of source rocks. In contrast, the HI of the source rock samples in the WYs sag ranged between 107 and 387 mg/g (average 243 mg/g), indicating the II_2_–III kerogen type of source rocks (Figure 4E). Samples in the five sags had *T*_max_ values ranging from 425 to 457 °C, which are suggestive of the low-mature to mature thermal evolutionary stage of the source rocks. There were samples with HI > 650 mg/g, suggesting the I kerogen type in the BaI-stepped belt, BNAN sub-sag (Figure 4A), WLHs sub-sag, Tumuer and Honggeer uplifts (Figure 4B), Anan and Hanan anticlines, Anan sub-sag (Figure 4C), and Aer central anticline/sub-sag (Figure 4D). However, most mudstone samples had HI < 400 mg/g in the Anan slope (Figure 4C), the Aer western and eastern slopes (Figure 4D), the Hongjing and Saiwusu uplifts (Figure 4B), and the WYs sag (Figure 4E). Overall, the dolomitic mudstone mostly had HI > 400 mg/g (average 568 mg/g), which is indicative of the I–II_1_ kerogen type (Figure 4F).

**Figure 5:**
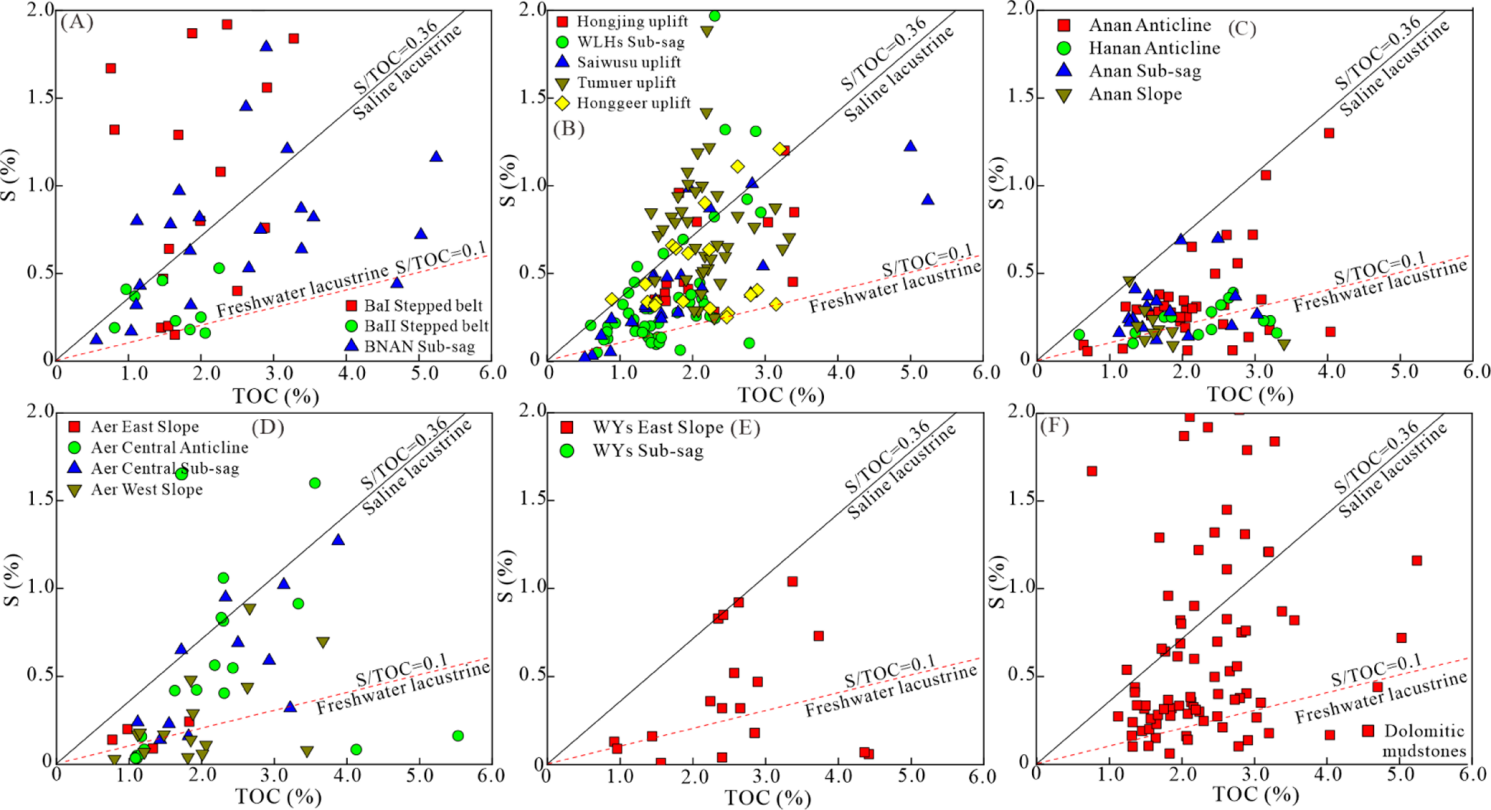
Plot of TOC vs sulfur (S) content reflecting the depositional water salinity conditions of the source rocks (A–E) and dolomitic mudstone (F) in Member 4 of the Aershan Fm and Member 1 of the Tengger Fm in different structural zones of the BNAN (A), WLHs (B), Anan (C), Aer (D), and WYs (E) sags in the Erlian Basin.

The TOC, sulfur content, and S/C ratio of the source rock samples in Member 1 of the Tengger Fm and Member 4 of the Aershan Fm in the five sags are shown in Table S1 and Figure 5. The TOC ranged between 0.56 and 5.24% (average 2.21%) in the BNAN sag, 0.52 and 5.24% (average 1.92%) in the WLHs sag, 0.58 and 4.12% (average 2.1%) in the Anan sag, 0.77 and 5.53% (average 2.07%) in the Aer sag, and 0.92 and 5.11% (average 2.65%) in the WYs sag. The sulfur content in the source rock samples ranged from 0.12 to 3.43% (average 0.84%) in the BNAN sag, 0.02 to 1.98% (average 0.52%) in the WLHs sag, 0.06 to 1.3% (average 0.3%) in the Anan sag, 0.03 to 1.69% (average 0.42%) in the Aer sag, and 0.01 to 1.04% (average 0.41%) in the WYs sag. The S/C ratio of the samples ranged from 0.08 to 2.2 (average 0.43) in the BNAN sag (Figure 5A), 0.03 to 0.94 (average 0.27) in the WLHs sag (Figure 5B), 0.02 to 0.36 (average 0.15) in the Anan sag (Figure 5C), 0.02 to 0.46 (average 0.17) in the Aer sag (Figure 5D), and 0.01 to 0.35 (average 0.16) in the WYs sag (Figure 5E). An average S/C ratio >0.36 was found in the samples of the BaI stepped belt, the BNAN sub-sag, the Tumuer uplift, and the Honggeer uplift (Figure 5A,B). Average S/C ratios between 0.1 and 0.36 were found for samples in the BaII stepped belt, the Anan anticline, the Aer sub-sag, the Saiwusu and Hongjing uplifts, and the WLHs sub-sag (Figure 5A–D). In contrast, an S/C ratio <0.1 was found for samples in the Anan slope, the Aer eastern slope, and the WYs sub-sag (Figure 5C–E). The dolomitic mudstone samples had S/C ratios in the range of 0.03–2.2 (average 0.31) (Figure 5F).

### 4.3 Biomarker Distribution

#### 4.3.1 *n*-Alkanes and Isoprenoids

**Figure 6:**
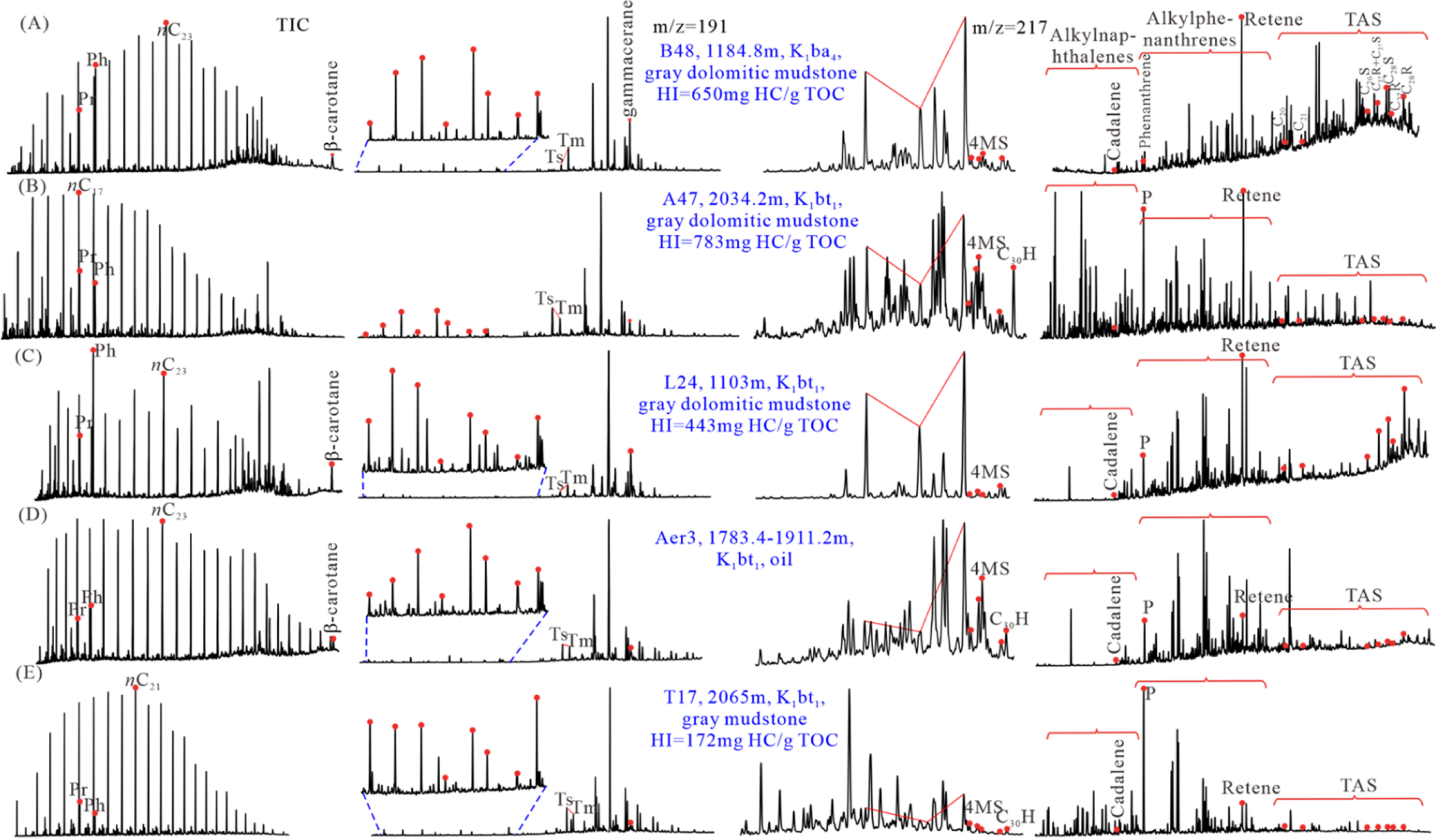
Representative total ion chromatogram of aliphatic, aromatic fractions, and mass chromatograms (*m*/*z* = 191, 217) of aliphatic fractions in the source rocks with different HIs in Member 4 of the Aershan Fm and Member 1 of the Tengger Fm in the BNAN, WLHs, Anan, Aer, and WYs sags in the Erlian Basin.

The parameters of *n*-alkanes, isoprenoids, and the representative total ion chromatogram of the aliphatic fractions in the 64 source rock samples and seven oil sand/oil samples are shown in Table 1 and Figure 6, respectively. The medium-chain (*n*C_21_–*n*C_25_) and long-chain (*n*C_27_–*n*C_31_) *n*-alkanes peaking at *n*C_23_, *n*C_25_, and *n*C_27_ accounted for the main fraction in the total ion chromatogram of the source rock samples in the WLHs sag. The short- and medium-chain *n*-alkanes peaking at *n*C_17_, *n*C_19_, *n*C_21_, and *n*C_23_ constituted the main fraction in the total ion chromatogram of the source rocks in the BNAN, Aer, and WYs sags. In contrast, source rock samples in the Anan sag mostly displayed a short-chain *n*-alkane-dominated total ion chromatogram with the main carbons *n*C_17_ and *n*C_19_ (Table 1). Abundant 17α(H),21β(H)-C_30_ hopanes (C_30_H) were detected in the total ion chromatogram of the samples in the BNAN, WLHs, and Anan sags. Of the five sags, the source rocks in the BNAN sag had the lowest pristane/phytane ratios (Pr/Ph) (0.2–0.75, average 0.4), the highest phytane/*n*C_18_ ratios (Ph/*n*C_18_) (0.61–4.41, average 1.99), and the highest β-carotane/*n*C_max_ ratios (C_max_ was the predominant *n*-alkane in the total ion chromatogram) (0.12–1.0, average 0.43), followed by the WLHs sag (0.23 < Pr/Ph < 1.07, 0.12 < Ph/*n*C_18_ < 5.17, 0.03 < average β-carotane/*n*C_max_ < 0.48). Source rocks in the Anan sag and the Aer sag had Pr/Ph ranging from 0.5 to 1.39 (average 0.85), Ph/*n*C_18_ ranging from 0.14 to 2.61 (average 0.49), and β-carotane/*n*C_max_ ranging from 0.01 to 0.23 (average 0.09). In contrast, source rocks in the WYs sag had the highest Pr/Ph (0.55–2.49, average 1.4), the lowest Ph/*n*C_18_ (0.17–1.22, average 0.32), and no β-carotane was detected.

#### 4.3.2 Steranes and Terpanes

The parameters of steranes and terpanes and the representative mass chromatograms of the aliphatic fractions in the 64 source rock samples and seven oil sand/oil samples are shown in Table 2 and Figure 6, respectively. The sterane/hopane (S/H) ratios of source rock samples ranged from 0.18 to 1.8 (average 0.65) in the BNAN sag, 0.12 to 1.64 (average 0.52) in the WLHs sag, 0.07 to 1.24 (average 0.42) in the Anan sag, 0.16 to 0.8 (average 0.33) in the Aer sag, and 0.18 to 0.49 (average 0.28) in the WYs sag. In the WLHs sag, the S/H ratio in the source rocks of the Tumuer and Honggeer uplifts (0.27–1.64, average 0.80) was higher than that in the WLHs sub-sag (0.12–0.52, average 0.31) and much higher than that in the Saiwusu and Hongjing uplifts (0.15–0.24, average 0.18). Abundant 4-methylsteranes were detected in samples of the Aer and Anan sags, whereas no 4-methylsteranes or only trace 4-methylsteranes were detected in samples of the BNAN, WLHs, and WYs sags.

Prokaryote-derived hopanes are the most abundant biomarker group among terpane molecular components. C_30_H was detected as the major peak even in the mass chromatogram of sterane (*m*/*z* 217) in the source rocks of Anan (Figure 6B). Except for samples in the Anan sag that had C_23_ tricyclic terpane/C_30_ hopane (C_23_TT/C_30_H) in the range of 0.07–1.59 (average 0.32), most samples in the BNAN, WLHs, Aer, and WYs sags had C_23_TT/C_30_H < 0.1. The ratios of gammacerane/C_30_ hopane (Gam/C_30_H) were higher in the BNAN, WLHs, and Anan sags (0.02–0.94, average 0.34) than in the Aer and WYs sags (0.03–0.34, average 0.11). The ratios of C_19_ tricyclic terpane/C_23_ tricyclic terpane (C_19_/C_23_TT) were higher in the source rock extracts of the WYs, WLHs, and Aer sags (0.12–1.74, average 0.68) than those of the Anan and BNAN sags (0.04–0.67, average 0.19).

#### 4.3.3 Aromatic Hydrocarbons

As shown in Figure 6, obvious difference was observed in the total ion chromatogram of the aromatic hydrocarbons extracted from the source rock or oil samples in the five sags. Abundant polycyclic aromatic hydrocarbons such as triarylsteranes (TASs) were detected in the source rocks of the BNAN and WLHs sags, and abundant alkylphenanthrenes were detected in the source rocks of the Aer sag, whereas moderate or abundant alkylnaphthalenes were detected in the source rocks of the Anan and WYs sags. All samples were found to contain a high abundance of retene and a low abundance of cadalene.

Mono-, di-, and trimethylated 2-(4,8,12-trimethyltridecyl) chromans (MTTCs) compounds were identified in the *m*/*z* 121, 135, and 149 mass chromatograms of the aromatic hydrocarbon fractions. In the BNAN sag, abundant 8-methyl-MTTC (δ-MTTC) is detected in the source rock extracts and the MTTC-ratio ranges from 0.65 to 0.89 (Table 2). In contrast, δ-MTTC was not detected in the source rock extracts of the Anan and WLHs sags and the MTTC ratio ranges from 0.76 to 1. Only 5,7,8-trimethyl-MTTC (α-MTTC) was detected in the aromatic fractions of source rocks in the Saiwusu and Hongjing uplifts, the Aer sag, and the WYs sags, and the MTTC ratio was approximately 1.0.

### 4.4 Elemental Geochemical Characteristics

The concentrations of the trace and major elements are listed in Table 3. The Sr/Ba ratio ranged from 0.3 to 1.96 (average 0.79) in the BNAN sag, 0.36 to 1.74 (average 0.67) in the WLHs sag, 0.31 to 1.65 (average 0.96) in the Anan sag, 0.23 to 0.43 (average 0.34) in the Aer sag, 0.37 to 0.41 (average 0.39) in the WYs sag. In source rock samples of the five sags, the concentrations of Co ranged from 4.09 to 23.2 ppm (average 12.8 ppm), which were similar to the average of the upper continental crust (Co average 10 ppm). However, the concentrations of Zn and Ni in the samples of the five sags and Fe in the samples of the BNAN, WLHs, and Anan sags ranged from 26.8 to 261 ppm (average 108.6 ppm), 10.7 to 138 ppm (average 48.1 ppm), 0.85 to 7.37% (average 4.0%), respectively, which are clearly higher than those of the upper continental crust (Zn average 71 ppm, Ni average 20 ppm, and Fe average 3.5%).^51^

The concentrations of REEs are listed in Table 4. In the BNAN, Anan, Aer, and WYs sags, the ∑LREE/∑HREE ratios were in the range of 5.15–10.27. The total concentration of REEs (∑REE) in the source rock samples ranged from 86.39 to 166.46 ppm (average 134.13 ppm) in the BNAN sag, 174.03 to 220.62 ppm (average 192.75 ppm) in the Anan sag, 178.26 to 304.64 ppm (average 254.24 ppm) in the Aer sag, and 239.7 to 288.58 ppm (average 264.14 ppm) in the WYs sag. A clear negative Eu anomaly (δEu = 0.39–0.6) was detected in all the samples in the five sags. Samples in the Anan and BNAN sags mainly had weak negative Ce anomalies (δCe = 0.86–0.96), whereas samples in the Aer and WYs sags simultaneously had a weak negative Ce anomaly (δCe = 0.89–0.95) and positive Ce anomaly (δCe = 1.24–1.29).

**Table 4:** REE Data and Related Parameters in the Source Rocks from Member 4 of the Aershan Fm and Member 1 of the Tengger Fm in the BNAN, Anan, Aer, and WYs Sags in the Erlian Basin^a^

no.	well	strata	depth (m)	lithology	La (ppm)	Ce (ppm)	Pr (ppm)	Nd (ppm)	Sm (ppm)	Eu (ppm)	Gd (ppm)	Tb (ppm)	Dy (ppm)	Ho (ppm)	Er (ppm)	Tm (ppm)	Yb (ppm)	Lu (ppm)	∑REE (ppm)	∑LREE/∑HREE	δEu	δCe
1	T11	K_1_bt_1_	1594.49	MD	52	135	12.6	50	9.9	1.73	7.87	1.45	6.99	1.3	3.92	0.673	4.46	0.69	288.58	9.55	0.60	1.29
2	T3	K_1_bt_1_	1401.25	MD	49.2	94.1	12.1	46.3	9.4	1.72	7.74	1.46	7.21	1.27	3.79	0.647	4.14	0.62	239.70	7.92	0.62	0.95
3	B9	K_1_ba_4_	1683.36	MD	36.7	61.4	7.78	31	6.17	1.15	7.09	1.1	5.27	0.974	3.08	0.505	3.71	0.53	166.46	6.48	0.53	0.89
4	B15	K_1_ba_4_	1407.3	DMD	31.4	54.2	7.69	29	5.79	0.917	4.64	0.897	5	0.942	2.86	0.483	3.21	0.48	147.51	6.97	0.54	0.86
5	B1	K_1_ba_4_	1212.83	DMD	28.9	52.7	6.26	23.4	4.98	0.913	5.49	0.939	4.85	0.898	2.72	0.453	3.22	0.47	136.19	6.15	0.53	0.96
6	B2	K_1_bt_1_	978.28	MD	18.6	30.3	3.99	15.6	3.2	0.646	3.93	0.678	3.42	0.675	1.93	0.375	2.67	0.38	86.40	5.15	0.56	0.86
11	H36	K_1_bt_1_	1362.9	MD	37.5	64.2	8.31	33.5	6.65	1.29	7.42	1.16	5.28	0.972	3.06	0.495	3.67	0.53	174.03	6.71	0.56	0.89
12	H20	K_1_bt_1_	1752.96	MD	40.5	71.6	8.83	32.5	6.11	1.07	5.37	1.08	6.08	1.21	3.73	0.653	4.23	0.64	183.61	6.98	0.57	0.93
13	H20	K_1_bt_1_	1754.41	MD	45.5	87.9	11	41.9	8.02	1.32	6.49	1.27	6.59	1.27	3.83	0.651	4.25	0.63	220.62	7.83	0.56	0.96
32	Aer3	K_1_bt_1_	1798.61	MD	56.1	139	13.5	51.3	11.2	1.74	8.93	1.73	8.69	1.58	4.48	0.775	4.83	0.78	304.64	8.58	0.53	1.24
33	Aer1	K_1_bt_1_	1468.2	MD	39.3	73	9.34	33.9	6.06	0.841	4.78	0.82	3.85	0.702	2.24	0.378	2.64	0.41	178.26	10.27	0.48	0.93
34	Aer5	K_1_bt_1_	1911.39	MD	56.4	94.2	12	46.4	9.24	1.25	10.2	1.63	8.09	1.52	4.82	0.793	5.58	0.81	252.94	6.56	0.39	0.89
35	Aer6	K_1_bt_1_	1650.6	MD	64.6	107	13.4	50.4	9.72	1.48	10.8	1.7	8.09	1.52	4.89	0.813	5.86	0.84	281.11	7.14	0.44	0.89

a ∑REE: total concentration of REE; ∑LREE/∑HREE: ratio of light REE to heavy REE; δEu=(Eu)_N_/SQRT(Sm*Gd)_N_, δCe=(Ce)_N_/SQRT(La*Pr)_N_, N means the normalized ratio of chondrites, and the chondrites data are reprinted with permission from Sun and McDonough^115^ Copyright 1989 Geological Society, London, Special Publications.

## 5 Discussion

### 5.1 Paleoenvironment of Source Rocks in Different Sags or Structural Zones

#### 5.1.1 Thermal Maturity

**Figure 7:**
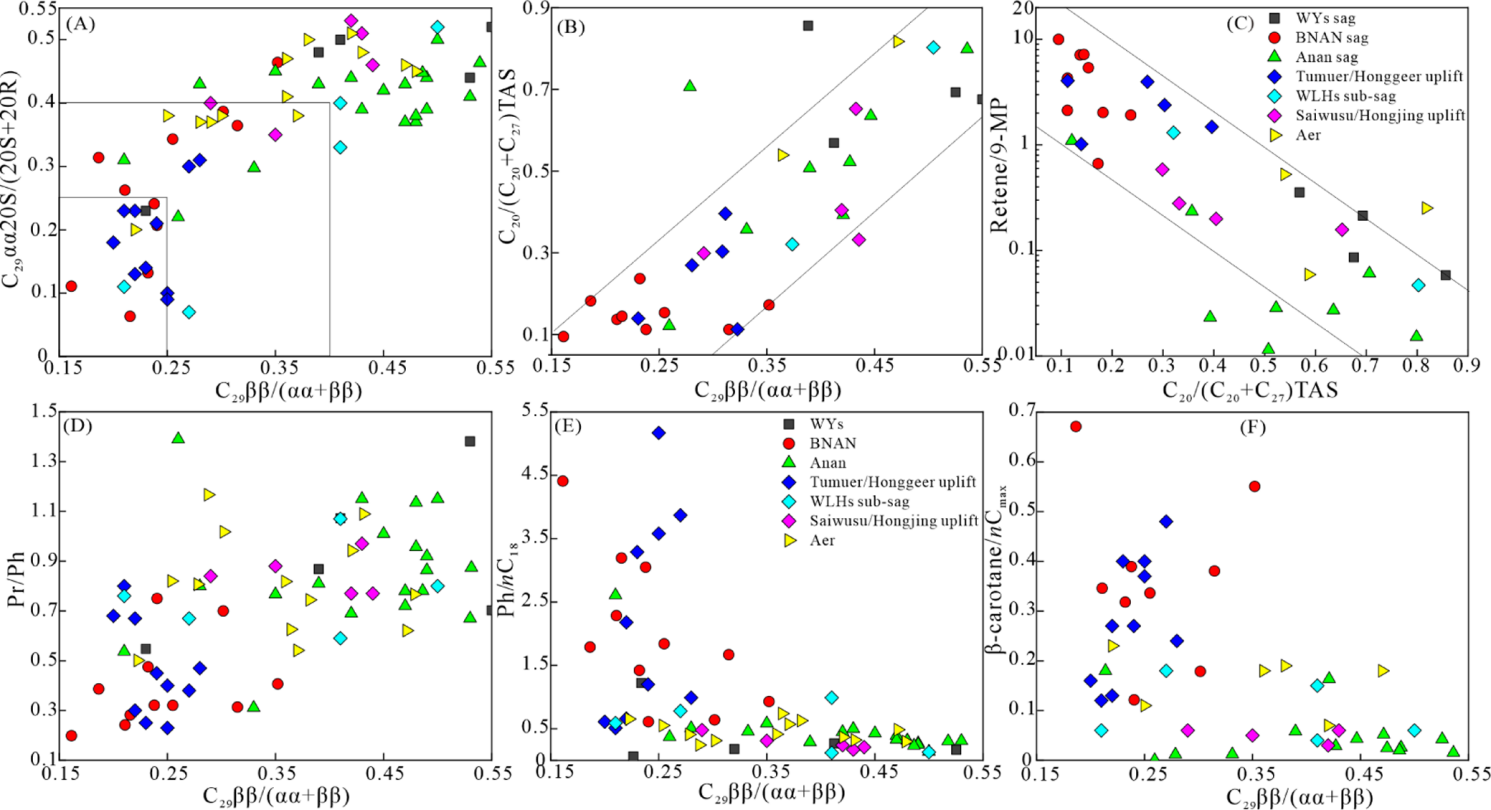
Scatter plots of (A) C_29_αα 20S/(20S + 20R) vs C_29_ββ/(ββ + αα), (B) C_20_/(C_20_ + C_27_) TAS vs C_29_ββ/(ββ + αα), (C) retene/9-MP vs C_29_ββ/(ββ + αα), (D) Pr/Ph vs C_29_ββ/(ββ + αα), (E) Ph/*n*C_18_ vs C_29_ββ/(ββ + αα), and (F) β-carotane/*n*C_max_ vs C_29_ββ/(ββ + αα), reflecting thermal maturity and its influence on the geochemical parameters of the source rocks of Member 4 of the Aershan Fm and Member 1 of the Tengger Fm in the BNAN, WLHs, Anan, Aer, and WYs sags in the Erlian Basin. The abbreviations for biomarker parameters are explained in Table 2.

The C_29_ββ/(ββ + αα) and C_29_αα20S/(20S + 20R) sterane ratios are commonly used maturity parameters in the immature to mature stage (*R*_o_ < 0.9%) because of the isomerization reaction of sterane. Triaryl steranes tend to crack from long-chain homologues to short-chain homologues as the thermal maturity increases from the immature stage to the wet gas window stage (*R*_o_ < 2.0%).^52^ There are obvious positive correlations between the C_29_ββ/(ββ + αα) and C_29_αα20S/(20S + 20R) sterane ratios (Figure 7A), C_29_ββ/(ββ + αα) and C_20_/(C_20_ + C_27_) TAS ratios (Figure 7B), suggesting the effectiveness of the three ratios in depicting the thermal maturity of the source rocks in the five sags. The C_29_ββ/(ββ + αα) ratio ranged from 0.16 to 0.55, without reaching the equilibrium endpoint values of 0.67–0.71, which indicates that the thermal maturity of the selected samples was not beyond the bottom threshold of the oil window (*R*_o_ < 1.0%).

The widely used higher plant input indicator of retene/9-methylphenanthrene (retene/9-MP)^53,54^ ranged from 0.05 to 10.0 but a low abundance of cadalene was detected in the samples of the BNAN sag and the WLHs sag (Figure 6). Besides that, the retene/9-MP ratio showed an exponential decrease with the C_20_/(C_20_ + C_27_) TAS ratio in the five sags (Figure 7C). These phenomena suggested that retene/9-MP served as a maturity indication^55^ rather than a higher plant input indicator in the Erlian Basin. C_29_ββ/(ββ + αα) was weakly positively correlated with Pr/Ph (Figure 7D) and only co-occurred with high ratios of Ph/*n*C_18_ and β-carotane/*n*C_max_ in the range of 0.15–0.4 (Figure 7E,F), suggesting the influence of thermal maturity on the parameters including Pr/Ph, Ph/*n*C_18_, and β-carotane/*n*C_max_. However, some immature and low-maturity samples in the Anan sag and Aer sag had higher Pr/Ph ratios and lower Ph/*n*C_18_ and β-carotane/*n*C_max_ ratios than the samples with similar thermal maturity in the BNAN and WLHs sags (Figure 7D–F). This indicated that the Pr/Ph, Ph/*n*C_18_, and β-carotane/*n*C_max_ ratios were not solely controlled by thermal maturity in the five sags. As the samples were in the immature to early mature stage, thermal maturity may not have radically affected the ratios of Pr/Ph, Ph/*n*C_18_, and β-carotane/*n*C_max_ to the extent that the original paleoenvironmental information is obscured.

#### 5.1.2 Redox Conditions

**Figure 8:**
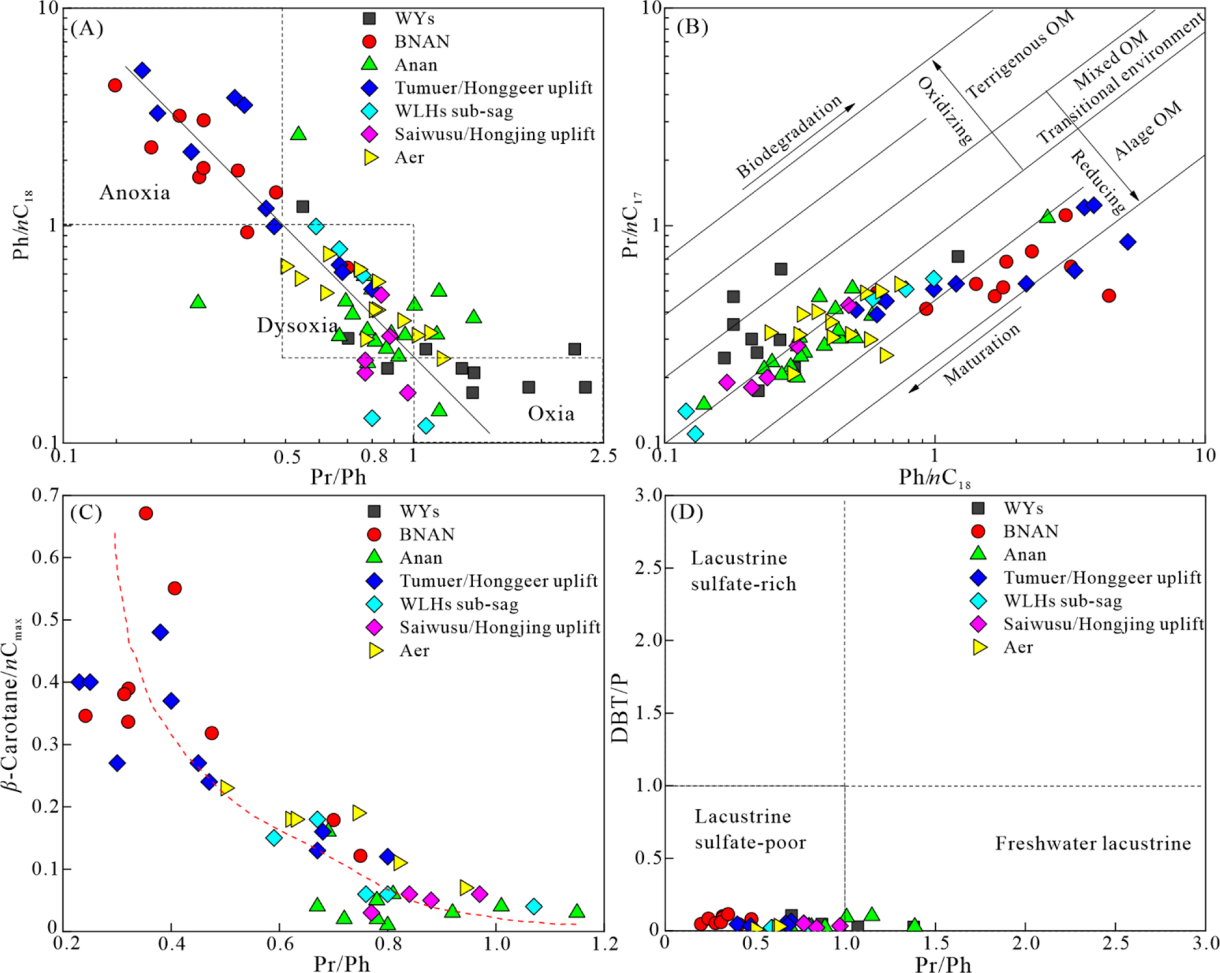
Scatter plots of (A) Ph/*n*C_18_ vs Pr/Ph, (B) Ph/*n*C_18_ vs Pr/*n*C_17_, (C) β-carotane/*n*C_max_ vs Pr/Ph, and (D) DBT/P vs Pr/Ph, reflecting the redox conditions in the source rocks of Member 4 of the Aershan Fm and Member 1 of the Tengger Fm in the BNAN, WLHs, Anan, Aer, and WYs sags in the Erlian Basin. Abbreviations for biomarker parameters are explained in Table 2.

The pristane/phytane ratio can be used to determine the redox conditions of the sediments during deposition in the context of both pristine and phytane originating from phytol in the side chain of most chlorophylls.^56^ During early diagenesis, a low Pr/Ph ratio (<1) is indicative of anoxic or dysoxic conditions,^56,57^ whereas Pr/Ph ratios >1 suggest oxic conditions.^56^ Due to the influence of specific sources of organic matter, such as halophilic archaea,^58^ extremely low Pr/Ph ratios (<0.5) are often characteristic of strictly anoxic and hypersaline conditions.^59^ A higher ratio of phytane/*n*C_18_ with a lower pristane/*n*C_17_ suggests a more reducing depositional condition, which otherwise implies more oxic conditions.^60^ β-Carotane is interpreted as a specific indicator of anoxic lacustrine or highly restricted marine environments due to its sensitivity to oxygen and low potential for preservation in sediments.^61,62^ There are obvious negative correlations between Pr/Ph and parameters including Ph/*n*C_18_ and β-carotane/*n*C_max_ in the source rocks of the BNAN, WLHs, Anan, and Aer sags (Figure 8A,C), indicating that these three parameters are effective indicators of redox conditions. The very low Pr/Ph (<0.5) ratios and the extremely high Ph/*n*C_18_ (>2.0) ratios, together with the poor positive correlation between Ph/*n*C_18_ and Pr/*n*C_17_ ratios for samples of the BNAN sag and the Tumuer/Honggeer uplifts (Figure 8B), suggest an additional source for phytane, such as ether lipids from the methanogenic archaea.^63,64^ It is reasonable to conclude that samples of the BNAN sag and the Tumuer/Honggeer uplifts were mainly deposited under anoxic conditions as the methanogenic archaea only thrive in certain restricted anoxic or hypersaline environments.^65^ The low to moderate Pr/Ph ratios (0.23–1.07) and wide range of Ph/*n*C_18_ ratios (0.12–5.17) and β-carotane/*n*C_max_ (0.03–0.48) for samples in the WLHs sag (Figure 8A,C) suggest a transition from anoxic conditions to dysoxic conditions. In contrast, the moderate to high Pr/Ph ratios (0.5–1.39), lower Ph/*n*C_18_ ratios (0.14–2.61), and lower β-carotane/*n*C_max_ ratios (0.01–0.23) for samples in the Anan and Aer sags suggest a transition from dysoxic to oxic conditions.

The DBT/P ratio reflects the availability of reactive sulfur for interactions with organic matter in the depositional environment.^65^ The plot of DBT/P versus Pr/Ph provides a novel, convenient, and powerful method applicable over a wide maturity range for determining the depositional environments and lithologies of the source rock.^65^ Samples in the BNAN and WLHs sags are plotted in the lacustrine sulfate-poor zone, whereas samples in the Anan, Aer, and WYs sags are plotted in the lacustrine sulfate-poor zone and freshwater lacustrine zone (Figure 8D). The results indicated that low amounts of reactive sulfur were present in the source rocks of the five sags. Source rocks in the BNAN and WLHs sags were deposited under anoxic conditions, whereas source rocks in the Anan, Aer, and WYs sags were deposited under transitional conditions ranging from anoxic to oxic conditions. It is worth noting that source rocks from the BNAN and WLHs sags displayed moderate sulfur contents (0.5–2%)^65^ (Figure 5A,B), which contrast with the low amounts of reactive sulfur. The detection of fine- to coarse-sized pyrite crystals in the matrix of the dolomitic rocks^29,66^ explains this paradox between the low DBT/P ratio and moderate sulfur content since sulfur is mainly present as sulfides rather than thiophenes in the dolomitic rocks of the BNAN and WLHs sags.

Redox-sensitive trace elements (e.g., Mo, U, V, Cr, and Mn) are widely used to decipher and reconstruct paleoenvironmental conditions.^67^ Previous studies^61,62^ have proposed that a V/(V + Ni) ratio <0.46 and Ni/Co ratio <5 point to oxic conditions, 0.46 < V/(V + Ni) ratio <0.6 and 5 < Ni/Co ratio <7 point to dysoxic conditions, and a 0.6 < V/(V + Ni) ratio and 7 < Ni/Co ratio point to anoxic conditions. As shown in Table 3, samples of the five sags display V/(V + Ni) ratios ranging from 0.31 to 0.84 with a mean of 0.66, suggesting predominantly dysoxic or anoxic conditions. In contrast, the Ni/Co ratios lie between 1.5 and 13.9 with a mean of 3.86 in samples of the five sags, suggesting predominantly oxic conditions. This contradiction may indicate that the redox-sensitive trace elements in the five sags are affected by hydrothermal ions, as reported in the hydrothermal deposits of Yin’e Basin.^14^ The Ce anomaly can serve as a redox proxy as a reducing environment generally leads to the depletion of Ce, whereas oxidizing conditions could contribute to the enrichment of Ce.^68^ The positive Ce anomaly coupled with the high Pr/Ph ratios (>1.0), low Ph/*n*C_18_ ratios (<0.3), and the absence of β-carotane in the total ion chromatogram plot for most samples in the WYs sag suggest a predominately oxic condition.

#### 5.1.3 Water Salinity

Gammacerane is thought to be formed by the reduction of tetrahymanol in bacterivorous ciliates that typically live in the chemocline or thermocline of stratified water columns.^69,70^ The high gammacerane index has been widely used to indicate evaporite or hypersaline environments^64,71^ as well as water column stratification.^72^ The extended tricyclic terpane ratio (ETR) is indicative of the salinity and alkalinity of sedimentary water^64^ because the fossil lipids of prokaryotes in saline and alkaline lakes are rich in precursors of extended tricyclic terpanes.^73^ The composition and distribution of MTTCs and the MTTC ratio in sediments can be used to reflect the paleosalinity of the depositional water.^74,75^ Empirically, the MTTCs of sediments from semi-saline to freshwater environments (i.e., <30‰) are characterized by a dominance of α-MTTC and a lack of δ-MTTC, with an MTTC ratio >0.7. The MTTCs of sediments from the mesosaline environments (40–120‰) were characterized by a prominent α-MTTC relative to δ-MTTC, with 0.4 < MTTC ratio < 0.7. The MTTCs of sediments from hypersaline environments (i.e., >120‰) are characterized by a prominent δ-MTTC relative to α-MTTC, with MTTC ratio < 0.4.^76,77^ Triaryl steranes tend to be abundant in the immature or low-mature organic matter of saline environments.^78^

**Figure 9:**
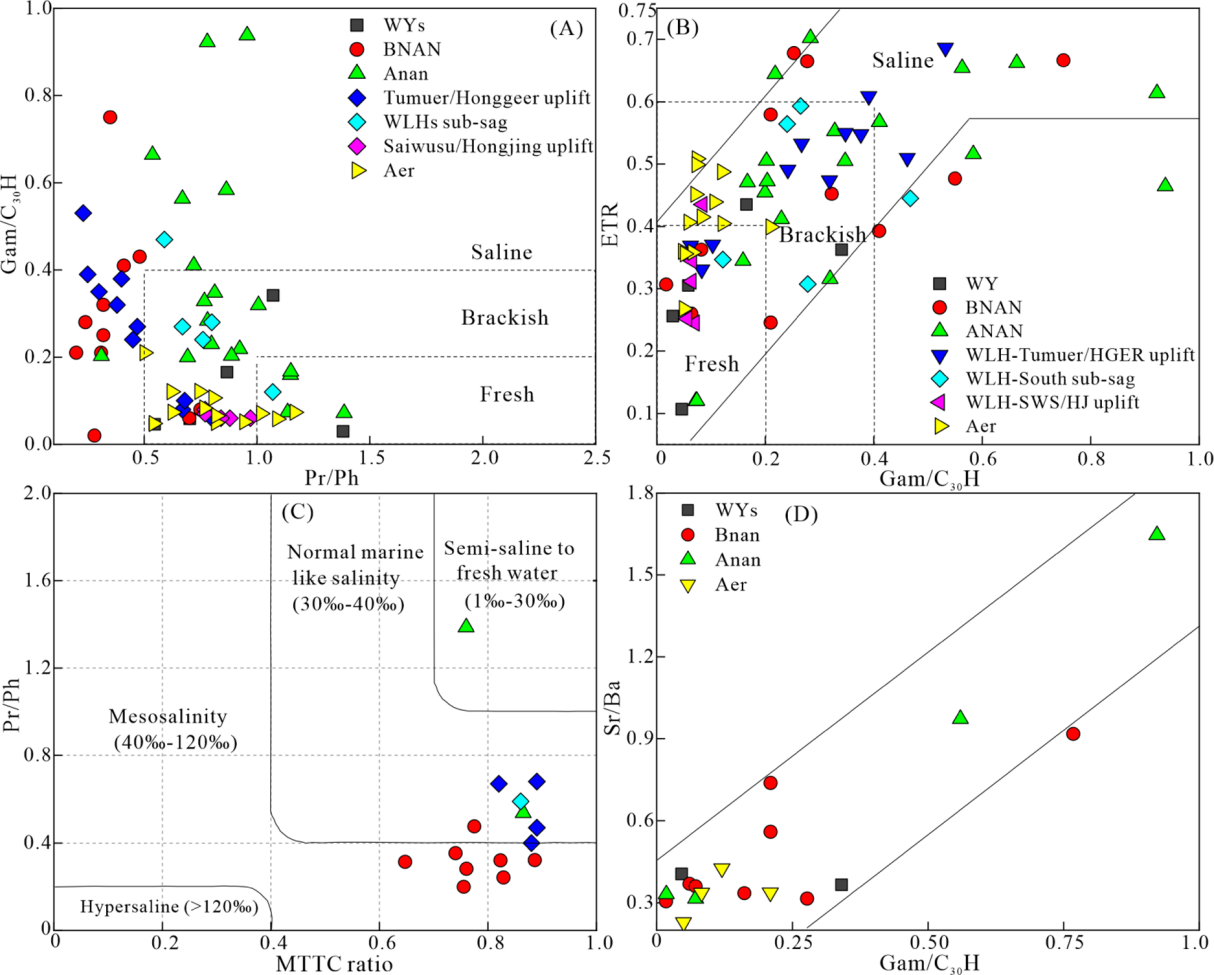
Scatter plots of (A) Pr/Ph vs Gam/C_30_H, (B) ETR vs Gam/C_30_H, (C) Pr/Ph vs MTTC ratio, and (D) Sr/Ba vs Gam/C_30_H, reflecting the water salinity conditions of the source rocks in Member 4 of the Aershan Fm and Member 1 of the Tengger Fm in the BNAN, WLHs, Anan, Aer and WYs sags in the Erlian Basin. Abbreviations for biomarker parameters are explained in Table 2.

The Gam/C_30_H ratio decreased with an increasing Pr/Ph ratio (Figure 9A), indicating that the salinity in the water body is directly controlled by redox conditions. A close positive correlation between Gam/C_30_H and ETR was found (Figure 9B), suggesting that Gam/C_30_H and ETR were effective indicators of water salinity in the five sags. In the crossplot of Pr/Ph versus MTTC ratio (Figure 9C), samples of the BNAN sag and WLHs sag are plotted in the areas of mesosalinity and normal marine salinity, whereas samples of the Anan sag are plotted in areas of normal marine salinity and brackish to fresh water. The low MTTC ratio (<0.7), high Gam/C_30_H ratio (>0.4), and ETR (>0.6), together with the detection of abundant triaryl steranes in samples of the BNAN sag and the Tumuer/Honggeer uplifts, suggest a predominant mesosaline condition. The moderate MTTC ratio (0.7–1), wide range of Gam/C_30_H ratios (0.07–0.94), ETR (0.12–0.7) in the Anan sag, and the WLHs sub-sag suggest transition conditions from freshwater to saline water. The high MTTC ratio and low to moderate Gam/C_30_H ratio (<0.4), ETR (<0.6), and the absence of triaryl steranes in samples of the Saiwusu/Hongjing uplift, Aer sag, and WYs sag suggest a predominate brackish to fresh condition.

The S/C ratios can be used to distinguish freshwater (or slightly brackish) from saline phases of ancient lakes because much lower concentrations of dissolved sulfate have been found in freshwater than in saline water.^79^ Sediments in saline or brackish lakes are characterized by S/C ratios >0.1, whereas freshwater sediments are characterized by S/C ratios <0.1.^79,80^ The Sr concentrations and Sr/Ba ratios in sediments are sensitive indicators for the discrimination of paleosalinity. Generally, Sr/Ba ratios of <0.6, 0.6–1.0, and >1.0 are indicative of freshwater, brackish water, and saline water, respectively.^8,81^

The Sr/Ba ratio showed a general positive increase with Gam/C_30_H (Figure 9D), suggesting the potential of the Sr/Ba ratio as an indicator of salinity. Although Sr/Ba ratio <0.6 and Gam/C_30_H < 0.2 were detected in the BNAN, WLHs, and Anan sag samples, most dolomitic mudstone samples in these sags had an S/C ratio >0.1 (Figure 5F) and Gam/C_30_H > 0.2 (Table 2). This result supports previous observations that dolomites are formed in concentrated alkaline lakes but are absent in freshwater lakes.^62,63^ In the BaI-stepped belt, BNAN sub-sag, Tumuer/Honggeer uplift, WLHs sub-sag, Anan anticline, and Anan sub-sag, the detection of the S/C ratio ≥ 0.36, average thickness of dolomitic mudstone >100 m, coupled with Gam/C_30_H > 0.4, and ETR > 0.6 suggest a predominate mesosaline or saline condition during the deposition of source rocks in Member 1 of the Tengger Fm and Member 4 of the Aershan Fm. In the BaII stepped belt, Saiwusu/Hongjing uplift, Hanan anticline, Aer central anticline/sub-sag, Aer western slope, and WYs sub-sag, the detection of an S/C ratio of 0.1 < S/C ratio < 0.36 and dolomite thickness ratio of 0 m < average thickness of dolomitic mudstone <100 m, coupled with the 0.2 < Gam/C_30_H < 0.4 and 0.4 < ETR < 0.6 suggest a predominate brackish condition during the deposition of source rocks in Member 1 of the Tengger Fm and Member 4 of the Aershan Fm. In the Anan slope, Aer eastern slope, and WYs eastern slope, the detection of S/C ratio <0.1, Gam/C_30_H < 0.2, and ETR < 0.4 suggests a predominant freshwater condition during the deposition of source rocks in Member 1 of the Tengger Fm and Member 4 of the Aershan Fm.

### 5.2 Sources of Organic Matter in Different Sags

Sterane parameters have the potential to reflect the presence of primary producers in lacustrine systems.^82^ C_28_ regular steranes in Cenozoic sediments are thought to be associated with specific phytoplankton types, such as diatoms^64,83^ that contain chlorophyll-c,^84^ whereas C_28_ regular steranes in pre-Cenozoic strata are ascribed to the elevated abundance of prasionphyte green algae.^85,86^ In addition, higher plants are also precursors of C_28_ steranes.^87^ C_29_ regular steranes originate from terrestrial organic matter^87^ and freshwater microalgae.^88^ The 4-methylsteranes are believed to be associated with dinoflagellates that are commonly found in freshwater environments.^89^ The relative abundance of hopanes is suggested to indicate the contribution of bacterial biomass to the organic matter,^90^ and the S/H ratio can effectively reflect the input of eukaryotic (mainly algae and higher plants) versus prokaryotic (bacteria) organisms.^82^ Tricyclic terpane hydrocarbons have been identified as major biomarkers in *tasmanite* algae, which are a type of green algae with close biological affinities to the present-day marine organism *Pachysphaera pelagica*.^91^ Some researchers have also concluded that bacteria^92^ and land plants^93^ are likely precursors of tricyclic terpane. Tetracyclic terpanes seem to indicate terrigenous organic matter input in lacustrine source rocks and oils.^94^ High ratios of C_19_/C_23_TT and C_24_Tet/C_23_TT are widely used to indicate important contributions from terrestrial organic matter, whereas high ratios of C_23_TT/C_30_H often indicate important contributions from specific microorganisms.^95^

**Figure 10:**
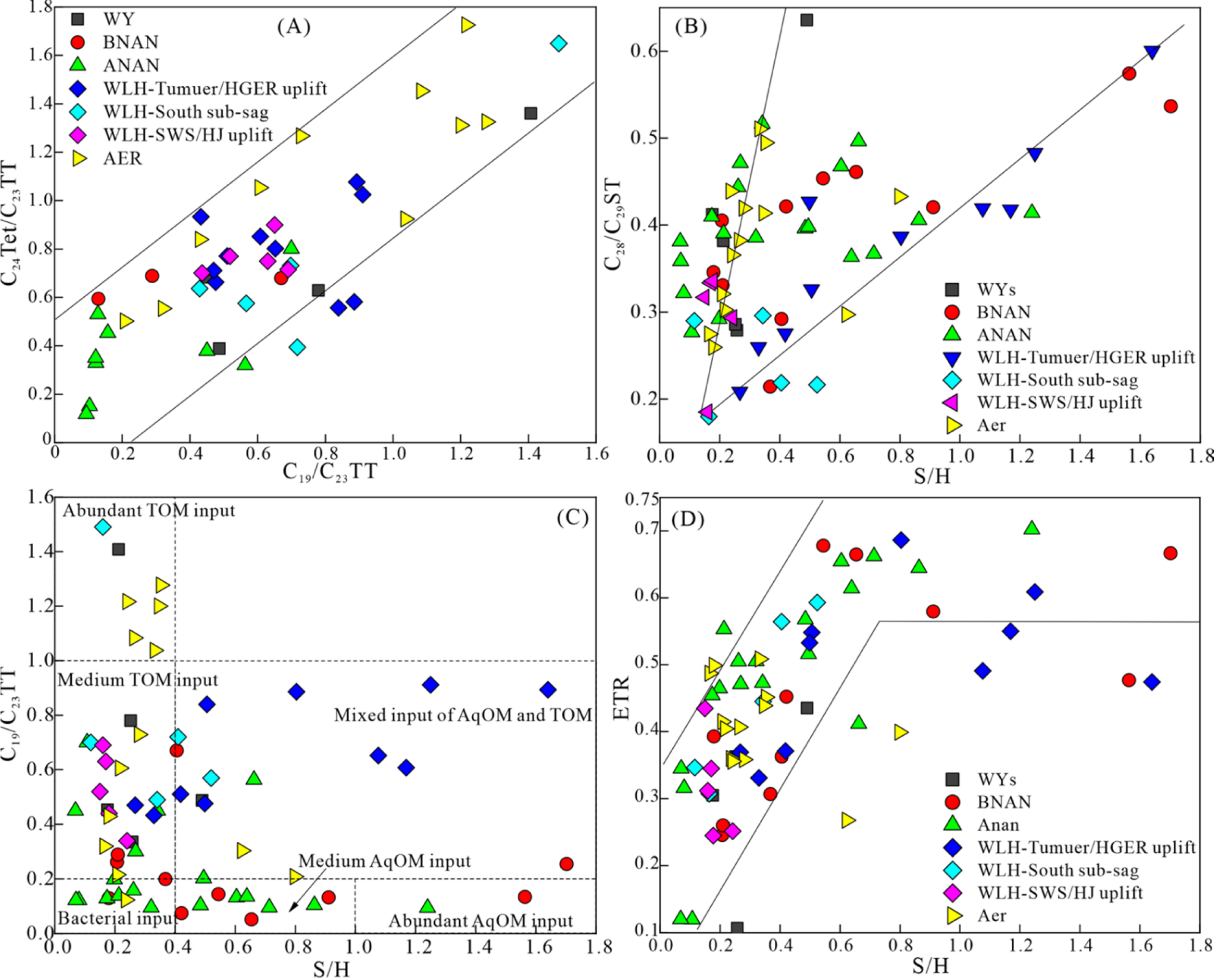
Scatter plots of (A) C_24_Tet/C_23_TT vs C_19_/C_23_TT, (B) S/H vs C_28_/C_29_ST, (C) S/H vs C_19_/C_23_TT, and (D) S/H vs ETR, reflecting organic matter sources in the source rocks of Member 4 of the Aershan Fm and Member 1 of the Tengger Fm in the BNAN, WLHs, Anan, Aer, and WYs sags in the Erlian Basin (TOM = terrigenous organic matter; AqOM = aquatic organic matter).

A positive correlation was found between the C_19_/C_23_TT and C_24_Tet/C_23_TT ratios of the samples (Figure 10A), suggesting the availability of C_19_/C_23_TT and C_24_Tet/C_23_TT as indicators of terrestrial organic matter input. Positive correlations were found between the S/H and C_28_/C_29_ST ratio in the BNAN sag and the WLH sag(Figure 10B), suggesting that high S/H ratio in the saline water sags of Erlian Basin was caused by contribution from prasionphyte green algae rather than C_29_ sterane-producing organisms. As the prasionphyte green algae were deposited under oxygen-depleted conditions that are unfavorable for most other photoautotrophic plankton species,^86,96^ the S/H ratio may reflect the primary productivity of the source rock depositional environment. The similar C_28_/C_29_ ratios but clearly lower S/H ratio in samples of the WYs, Aer, and Anan sags in comparison to that in the WLHs and BNAN sags are likely the contribution of bacteria or terrigenous organic matter.

Most source rock samples in the Anan and BNAN sags and two samples in the Aer sag have C_19_/C_23_TT ratios <0.2 and S/H ratios ranging from 0.4 to 1.7 (average 0.77) (Figure 10C), indicating low contributions from higher plants but an important contribution from phytoplankton and metazoa in the organic matter. In contrast, most source rock samples in the WYs and Aer sags have S/H ratios < 0.4 and C_19_/C_23_TT ratios ranging from 0.2 to 1.41 (average 0.62) (Figure 10C), indicating a low contribution from aquatic organisms but an important contribution from higher plants in terms of organic matter. Source rock samples in the WLHs sag simultaneously had high S/H and C_19_/C_23_TT ratios (Figure 10C), which suggests a mixed input of aquatic and terrestrial organisms in the organic matter. The high S/H ratio in the source rocks of the Tumuer and Honggeer uplifts, which are proximal to the boundary normal fault, suggests high primary productivity of the depositional water during the formation of source rocks in the footwall blocks of the boundary normal faults in the WLHs sag.

### 5.3 Organic Facies

**Figure 11:**
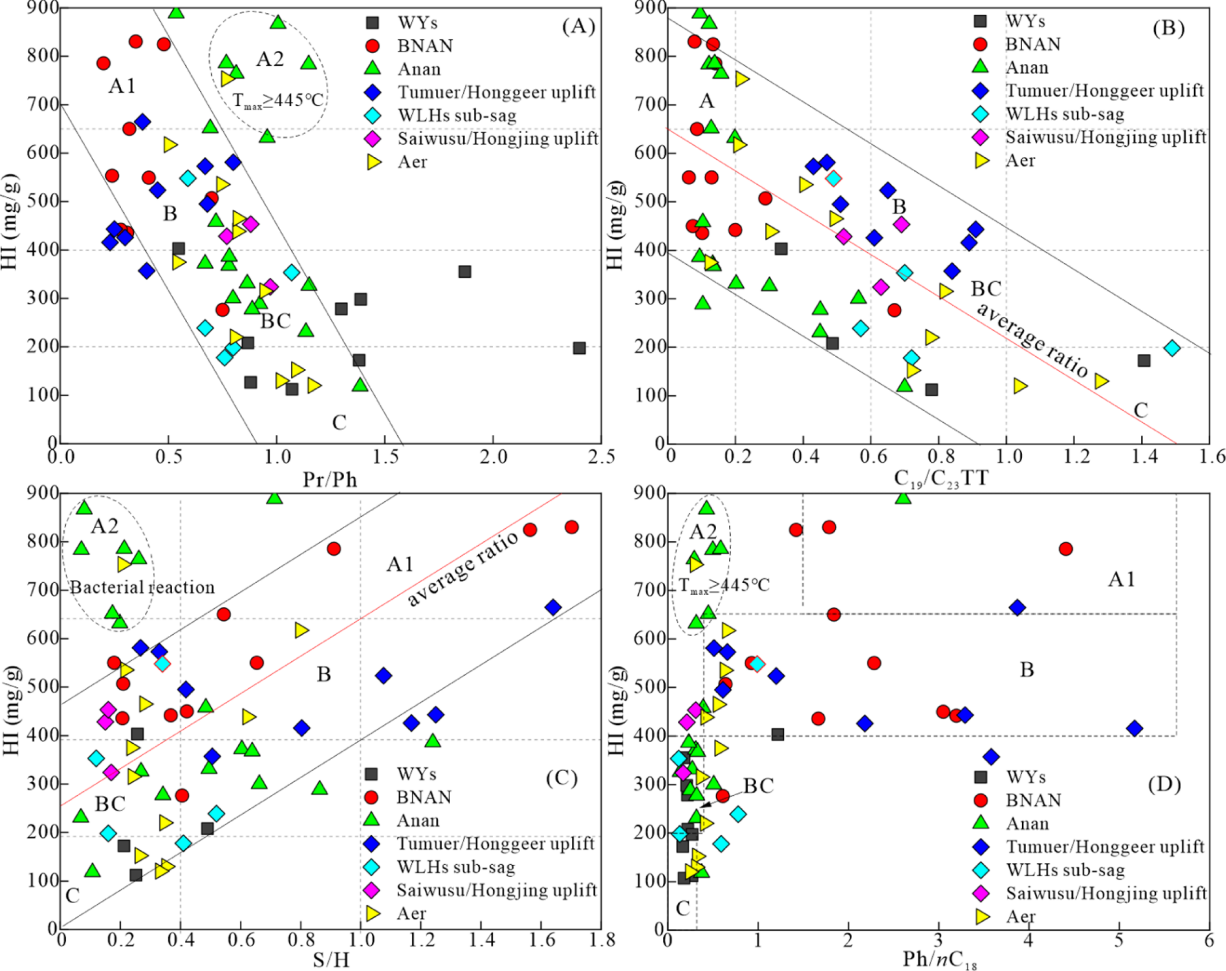
Correlations of (A) Pr/Ph vs HI, (B) C_19_/C_23_TT vs HI, (C) S/H vs HI, and (D) Ph/*n*C_18_ vs HI, illustrating the factors controlling the formation of high-quality source rocks in Member 4 of the Aershan Fm and Member 1 of the Tengger Fm in the BNAN, WLHs, Anan, Aer, and WYs sags in the Erlian Basin. Abbreviations for biomarker parameters are explained in Table 2.

In the BNAN and WLHs sags, there were negative correlations between HI and Pr/Ph (Figure 11A), HI and C_19_/C_23_TT ratios (Figure 11B), and positive correlations between HI and the S/H ratios (Figure 11C). HI > 650 mg HC/g TOC always occurred in samples with Ph/*n*C_18_ > 1.5 (Figure 11D). These characteristics indicate that a high HI is controlled by reductive conditions and the amount of aquatic organic matter input during the depositional process of source rocks in the BNAN and WLHs sags. In the Aer and Anan sags, there were poor relationships between HI and the parameters, including the Pr/Ph and S/H ratios (Figure 11A,C), as seven samples with HI > 650 mg HC/g TOC had Pr/Ph ratios ranging from 0.7 to 1.1 and S/H ratios <0.4. Coincidentally, these seven samples have *T*_max_ values >445 °C (Table 1) and have been detected C_30_H as the major peak in the mass chromatogram of sterane (*m*/*z* 217) (Figure 6B). These characteristics indicate that a high HI is likely controlled by an increase in bacterial input. All samples in the five sags displayed a positive correlation between the S/H ratio and ETR (Figure 10D), suggesting a close relationship between biological inputs and water salinity conditions. Specifically, an increase in lake water salinity may promote the proliferation of prasionphyte green algae and further increase water reducibility, thus contributing to the formation of high-quality source rocks.

Based on the HI and the correlations between HI and the selected parameters, including the Pr/Ph, Ph/*n*C_18_, S/H, and C_19_/C_23_TT ratios, four types of organic facies^20,97^ can be recognized (Table 5). Organic facies A has HI > 650 mg HC/g TOC and an S/C ratio≥0.36 and is mainly composed of thick dolomitic mudstone (average > 100 m) and mudstone. Source rocks in organic facies A were deposited in anoxic environments, with abundant input from aquatic organisms or bacteria and limited input from terrestrial higher plants. Owing to the difference in thermal maturity of the organic matter, organic facies A could be further divided into organic facies A1 and A2. Organic facies A1 had *T*_max_ <445 °C, with the biomarkers characterized by Pr/Ph ratios of <0.5, Ph/*n*C_18_ ratios of >1.5, β-carotane/*n*C_max_ ratios of >0.2, average S/H ratios of >1.0, and C_19_/C_23_TT ratios of <0.2. Organic facies A2 had *T*_max_ ≥445 °C, with the biomarkers characterized by 0.5 < Pr/Ph < 1, Ph/*n*C_18_ < 0.5, β-carotane/nC_max_ < 0.1, and C_19_/C_23_TT < 0.2. Organic facies B had 400 < HI < 650 mg HC/g TOC, 0.1 < S/C ≤ 0.36 and was composed of mudstone intercalated with thin layers of dolomitic mudstone (average < 50 m). Source rocks in organic facies B were deposited in dysoxic environments, with a medium amount of aquatic organic matter input and a little-medium amount of terrestrial organic matter input. Biomarkers in organic facies B are characterized by 0.5 < Pr/Ph < 1, 0.4 < Ph/*n*C_18_, 0.1 < β-carotane/*n*C_max_ < 0.2, 0.2 < average ratio of C_19_/C_23_TT < 0.6, and 0.4 < average ratio of S/H < 1. Organic facies BC has 200 < HI < 400 mg HC/g TOC, 0.1 ≤ S/C < 0.36 and is mainly composed of mudstone that was deposited in dysoxic environments, with limited amounts of aquatic organic matter input and a medium amount of terrestrial organic matter input. Biomarkers in organic facies BC are characterized by 0.8 < Pr/Ph < 0, 0.3 < Ph/*n*C_18_ < 0.4, β-carotane/*n*C_max_ < 0.1, 0.6 < average ratio of C_19_/C_23_TT < 1.0, and an average ratio of S/H < 0.4. Organic facies C has HI < 200 mg HC/g TOC and S/C < 0.1 and is mainly composed of mudstone deposited in oxic and fresh environments, with abundant terrestrial organic matter input. Biomarkers in organic facies C are characterized by 1.0 < Pr/Ph, Ph/*n*C_18_ < 0.3, average ratio of Gam/C_30_H < 0.1, 1 < average ratio of C_19_/C_23_TT, and an average ratio of S/H < 0.4. Organic facies A1 was mainly distributed in the footwall blocks of boundary normal faults, such as the BaI-stepped belt, BNAN sub-sag, and Tumuer and Honggeer uplifts, whereas organic facies A2 was mainly distributed in the Anan sub-sag and Anan anticline. Organic facies B is distributed in the BaII stepped belt, Anan anticline, Anan sub-sag, WLHs sub-sag, and Aer sub-sag. Organic facies BC was widely distributed in all five sags in the Erlian Basin. In contrast, organic facies C is mainly distributed in the Anan slope, the Aer eastern slope, and the WYs sag.

**Table 5:** Organic Facies Division and Its Biomarker Characteristics for the Source Rocks in Member 4 of the Aershan Fm and Member 1 of the Tengger Fm in the BNAN, WLHs, Anan, Aer, and WYs Sags in the Erlian Basin^a^

organic facies	A1	A2	B	BC	C
kerogen type	I	II_1_	II_2_	III	
HI (mg/g)	>650	400–650	200–400	<200	
water chemistry	anoxic, saline	suboxic, brackish anoxic, saline	dysoxic, brackish	dysoxic	oxic, fresh
average thickness of dolomitic mudstones (m)	>100	0–50	0	0	
*T*_max_ (°C)	<445	≥445	<455	<455	<455
biofacies	AqOM	AqOM, bacteria	medium AqOM, little-medium TOM	medium TOM, little-medium AqOM	TOM
Pr/Ph	<0.5	0.5–1.0	0.5–1.0	0.8–1.0	>1.0
Ph/*n*C_18_	>1.5	<1.0	>0.4	0.3–0.4	<0.3
β-Carotane/nC_max_	>0.2	<0.1	0.1–0.2	<0.1	not detected
Average Gam/C_30_H	>0.4	>0.4	0.2–0.4	0.1–0.2	<0.2
S/C	≥0.36	≥0.36	0.1–0.36	0.1–0.36	<0.1
MTTC-ratio	0.6–0.9	not detected	0.7–1	only α-MTTC	not detected
average C_19_/C_23_TT	<0.2	<0.2	0.2–0.6	0.6–1.0	>1.0
average S/H	>1.0	<0.4	0.4–1.0	<0.4	<0.4
typical areas	BaI steeped belt, BNANsub-sag, Tumuer/Honggeer uplift	Anan sub-sag. Anan anticlines	Anan anticlines, Aer sub-sag, WLHs sub-sag, BaII stepped belt	BNAN, WLHs, Anan, Aer, WYs sags	WYs, Aer eastern slope, Anan slope

a Abbreviations for biomarker parameters are explained in Table 2.

### 5.4 Volcanic and Hydrothermal Activity and Its Influence on Source Rock Quality

#### 5.4.1 Identification of Volcanic and Hydrothermal Activity

Previous research has shown that dolomites in the Tengger Fm of the Lower Cretaceous are a set of “white chimney” hydrothermal sediments in the Baiyinchagan sag of the Chuanjing depression in the Erlian Basin.^98,99^ Yang et al.^98^ found that the dolomites developed proximally to the fault zones, with the thickness decreasing from the faults toward the center of the basin. Chen et al.^100^ and Xiang et al.^101^ identified that the hydrothermal dolomites in the Yin’e Basin and Bayan Gebi Basin have enriched Fe and Mn element contents, medium to strong negative δEu anomalies, and right-declined REE distributions. Hydrothermal and volcanic activity often occurs simultaneously.^13^ In the Anan and BNAN sags, baseline research of dolomites has shown that the micro-silty dolomites in the dolomitic mudstone are formed at temperatures between 52 and 75 °C, and the ion of Mg^2+^ for the formation of dolomites either originates from alteration of tuffaceous materials or from the deep fluid rich in tuffaceous materials.^29,35,66,102^ All of these findings suggest that the formation of dolomites in the Erlian Basin is associated with hydrothermal and/or volcanic activity.

**Figure 12:**
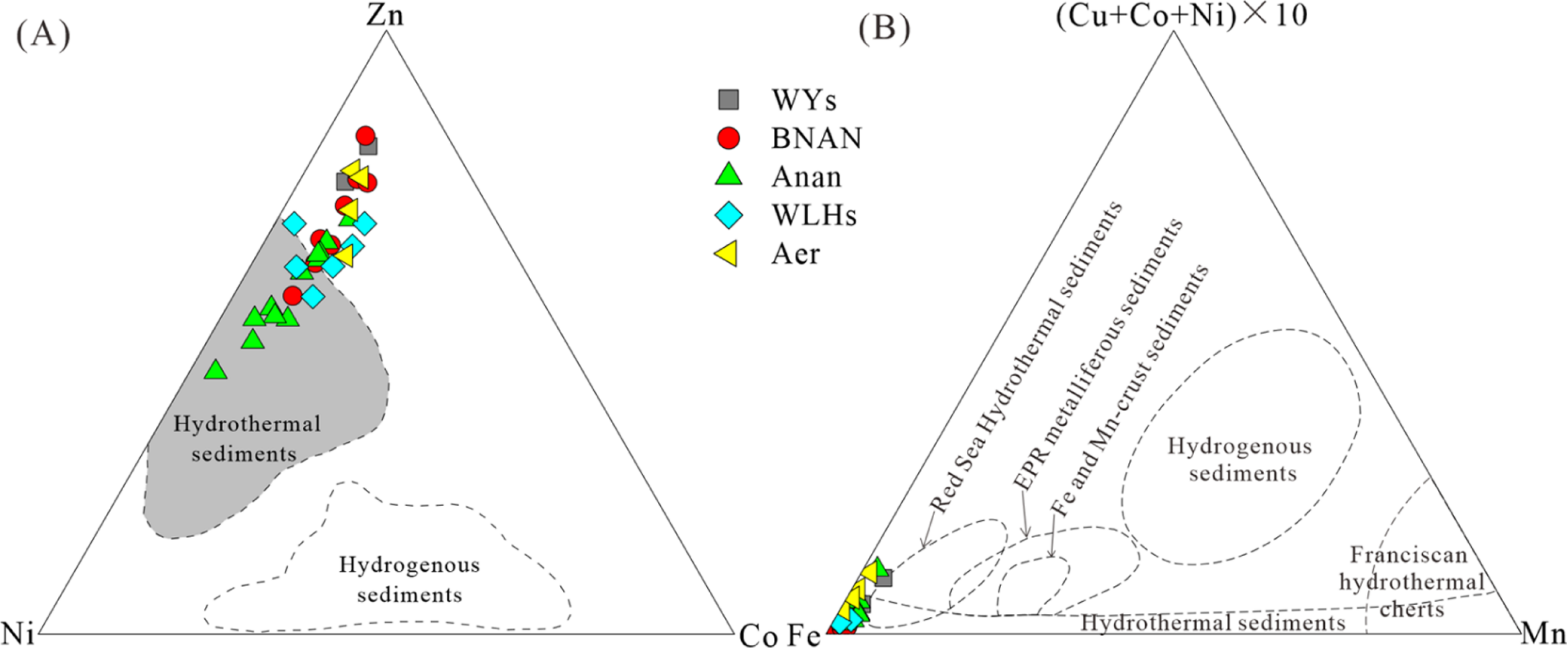
Triangular plot of Zn–Co–Ni (reprinted with permission from Choi and Hariya^118^ Copyright 1992 Economic geology and the bulletin of the Society of Economic Geologists) and (Cu + Co + Ni) × 10–Fe–Mn (reprinted with permission from Crerar et al.^119^ Copyright 1982 Economic geology and the bulletin of the Society of Economic Geologists) of the source rocks in Member 4 of the Aershan Fm and Member 1 of the Tengger Fm in the BNAN, WLHs, Anan, Aer, and WYs sags in the Erlian Basin.

**Figure 13:**
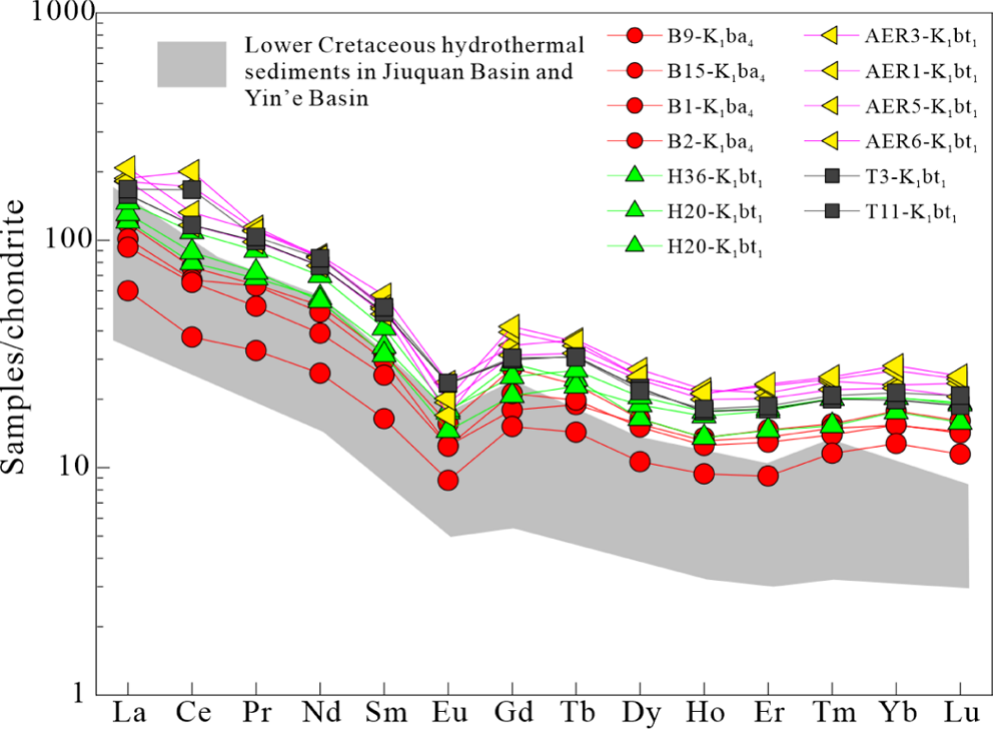
Chondrite-normalized REE distribution pattern of the source rocks in Member 4 of the Aershan Fm and Member 1 of the Tengger Fm in the BNAN, Anan, Aer, and WYs sags in the Erlian Basin. Distribution area of the hydrothermal sediments (gray) is reprinted with permission from Zhang et al.^8^ Copyright 2020 Palaeogeography, Palaeoclimatology, Palaeoecology.

In the BNAN, WLHs, and Anan sags, dolomitic mudstone in organic facies A–B (Figure 4F) is characterized by an uneven distribution, with the thickness of dolomitic mudstone decreasing in the order of the footwall blocks of boundary normal faults > the sub-sag zones > the slope zones opposite to the boundary faults (Figure 3). These characteristics suggest that the distribution of dolomitic mudstone is controlled by boundary faults similar to those of the Baiyinchagan sag. The ternary diagrams of Co–Zn–Ni and Fe–Mn–(Cu + Co + Ni) × 10 (Figure 12) both show that source rocks in the BNAN, WLHs, and Anan sags are plot in the hydrothermal sediment zone and the hydrogenous sediment zone, whereas source rocks in the Aer and WYs sags are mostly plot in the hydrogenous sediment zone. The two diagrams objectively illustrate that the source rocks in the BNAN, WLHs, and Anan sags are lacustrine sediments interbedded or mixed with hydrothermal sediments, unlike the normal lacustrine sediments in the Aer and WYs sags. Normalization with respect to chondrites showed a similar gentle right-declined REE concentration and a similar significantly negative Eu anomaly in all samples (Figure 13). These features are consistent with the REE geochemical features of lacustrine hydrothermal deposits in the Yin’e Basin,^8^ Bayan Gebi Basin,^101^ Jiuquan Basin,^103^ and Lake Tanganyika in East Africa,^104^ which might indicate that the hydrothermal fluids were mixed and cooled by the lake water.^103^ The lower concentration of ∑REE in the samples of the BNAN sag (Figure 13) indicates higher alkalinity of the depositional water, as the ∑REE content was reported to decrease with higher fluid pH,^105^ and may further suggest the presence of stronger hydrothermal activity in the BNAN sag. Additionally, the higher geothermal gradient in the Tumuer and Honggeer uplifts than in the Saiwusu and Hongjing uplifts^106^ also supports the presence of volcanic and/or hydrothermal activity in the WLHs sag.

#### 5.4.2 Influence of Volcanic and Hydrothermal Activity on the Source Rock Quality

**Figure 14:**
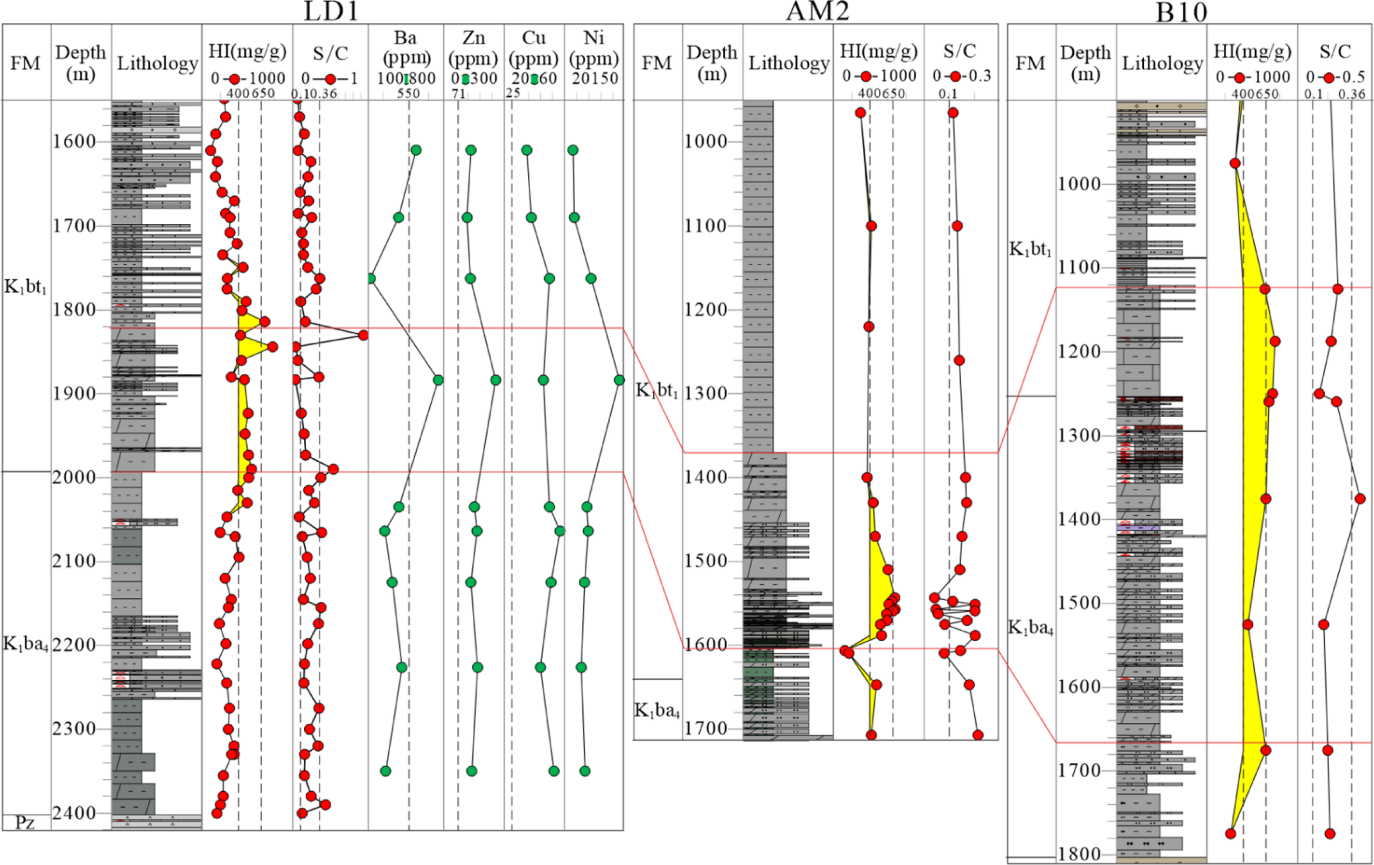
Connecting well profiles of the source rocks in Member 4 of the Aershan Fm and Member 1 of the Tengger Fm in the WLHs, BNAN, and Anan sags, showing the relative higher values of HI, S/C ratio, and content of Ba, Zn, Cu, and Ni in the dolomitic mudstone than that in the mudstone.

As shown in Figure 14, the dolomitic mudstone had an HI > 400 mg/g, while the mudstone had an HI < 400 mg/g in Member 1 of the Tengger Fm and Member 4 of the Aershan Fm in the LD1 well of the WLHs sag, the AM2 well of the Anan sag, and the B10 well of the BNAN sag. The S/C ratio in the mudstone of the three wells mostly ranged from 0.1 to 0.36. However, S/C ratios >0.36 were only found in the dolomitic mudstone of wells LD1 and B10, which indicates that the dolomitic mudstone was mainly deposited in anoxic and saline environments. The elemental composition of the dolomitic mudstone in the LD1 well displayed a relatively higher content of nutrients (Ba, Cu, Zn, and Ni)^67,107,108^ than that of the mudstone and much higher than the average values (Ba = 550 ppm, Cu = 25 ppm, Zn = 71 ppm, and Ni = 20 ppm) of the upper continental crust.^51^ These characteristics indicate that the primary productivity in the depositional water of the dolomitic mudstone was higher than that of mudstone. As the formation and distribution of dolomites are associated with volcanic and hydrothermal activity, anoxic and saline environments with high primary productivity are likely the result of volcanic and hydrothermal activity. Hydrothermal fluid and hydrolysis of volcanic ash may bring a large amount of reducing gas (SO_2_, H_2_S, etc.), soluble ionic compounds, and nutrients into lakes, which is beneficial for increasing anoxic and saline conditions, thus promoting the growth and reproduction of algae and bacteria.^8^

### 5.5 Depositional Models of Source Rocks in Different Sags

During the deposition of Member 1 of the Tengger Fm and Member 4 of the Aershan Fm in the Erlian Basin, the climate was warm and humid,^109^ resulting in abundant rainfall, short-rapid river flow, and weak evaporation. This type of paleoclimate is not conducive to the formation of saline lakes. However, the extensive tectonic magmatic event resulted in an active volcanic eruption, forming multiple sets of volcanic rocks in the Lower Cretaceous and Upper Jurassic. The intensity of volcanic activity varied greatly in different sags, which is indicated by the varying content of tuff and dolomitic mudstone in the Lower Cretaceous in different sags.^41^ The varying intensity of volcanic activity may have led to significant differences in the chemical properties of the depositional water in the diverse sags of the Erlian Basin.

**Figure 15:**
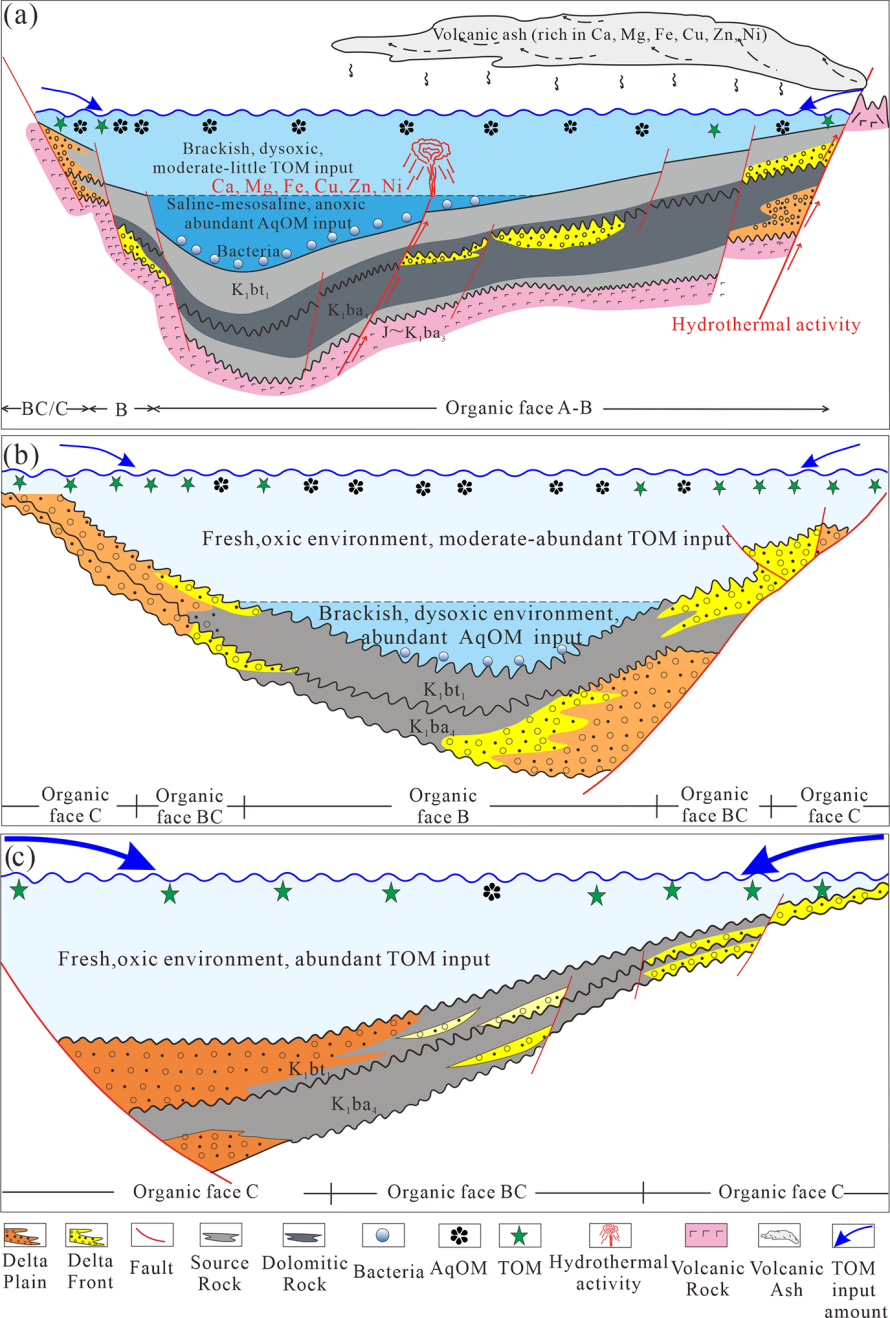
Depositional models and organic facies distribution for the source rocks in Member 4 of the Aershan Fm and Member 1 of the Tengger Fm in the BNAN, WLHs, Anan, Aer, and WYs sags in the Erlian Basin. (a) Saline to brackish environmental and ecological changes induced by intensive hydrothermal and volcanic activity in the BNAN, Anan, and WLHs sag. (b) Brackish to fresh environmental and ecological changes in the Aer sag. (c) Fresh environmental and ecological changes in the WYs sag.

In the BNAN, WLHs, and Anan sags of the Lower Cretaceous, there was intensive hydrothermal and volcanic activity, causing high contents of tuff, basalt, or andesite in Member 3 of the Aershan Fm and the Xing’anling Group (Figure 15a).^41,66,110,111^ During the deposition of Member 1 of the Tengger Fm and Member 4 of the Aershan Fm, the BaI fault and the eastern boundary fault in the BNAN sag, the Aershan fault in the Anan sag, and the eastern boundary fault of the WLHs sag were important channels of hydrothermal and volcanic activity.^37,66^ Hydrothermal fluid rich in Ca, Mg, Cu, Zn, and Ni in the deep sag surged up along the faults and mixed with the lake water. This process increased the water salinity and reducibility and was beneficial for the growth of plankton and bacteria.^112^ A clear evidence of the hydrothermal activity effect in the BNAN, WLHs, and Anan sags is the distribution of dolomitic mudstone on the descending side of the boundary faults, and the high HI, S/C, C_28_/C_29_ sterane, and S/H ratios in the dolomitic mudstone. At the slope opposite the faults, due to river injection and terrestrial organic matter input, the reducibility and paleo-productivity of the surface water were reduced. As a result, thick dolomitic mudstone in organic facies A and thin dolomitic mudstone in organic facies B were mainly deposited in the sub-sag zone and footwall blocks of the boundary faults. The organic facies BC/C were mainly distributed in the slope zones opposite the boundary fault (Figure 15a).

In the Aer Sag, the volcanic activity in the Lower Cretaceous was not intense, and no tuff or dolomites were deposited.^113^ During the deposition of Member 1 of the Tengger Fm and Member 4 of the Aershan Fm, the border faults had intensive structural activity, resulting in a narrow width (<10 km) but rapid subsidence of the sag. The low width/depth ratio further hampered efficient water mixing and favored stable water column stratification, which was corroborated by the medium gammacerane abundance and the two distinct correlation coefficients between the TOC and the S content (0.1 and 0.36, respectively, Figure 5D). In the deep lake, the water was suboxic and brackish with medium to high levels of primary productivity. This can be corroborated by the S/C ratio ranging from 0.1 to 0.36, S/H ratio >0.8 and occurrence of 4-methylsteranes in the source rocks of the Aer central sub-sag (Figure 6). Thus, the source rocks in the central lake were mainly composed of organic facies B. At the edge of the sag, source rocks were deposited in shallow and oxic environments with abundant terrestrial organic matter input, mainly organic facies BC/C (Figure 15b).

In the WYs sag, the intensity of volcanic activity in the Lower Cretaceous was not intense, and no tuff or dolomite was deposited (Figure 15c).^114^ During the deposition of Member 1 of the Tengger Fm and Member 4 of the Aershan Fm, the sag had a wider width (15 km) than that of the Aer sag. The high width/depth ratio was not efficient in hampering water mixing. Coupled with the abundant terrigenous clastic input, the source rocks were mainly deposited in fresh and oxic environments. Primary productivity was also reduced by the dilution effect of the large influx of terrigenous debris. Hence, the source rocks in the WYs sag consisted mainly of organic facies BC or C, which can be substantiated by the low gammacerane abundance, low S/C ratio, and HI < 400 mg HC/TOC.

## 6 Conclusions

Through comprehensive and comparative study of organic geochemical data and elemental geochemical data for source rocks in the Member 1 of Tengger Fm and Member 4 of Aershan Fm of the BNAN, WLHs, Anan, Aer, and WYs sags, the following conclusions can be drawn:

(1) The source rocks in the BNAN, WLHs, and Anan sags are composed of mudstone and dolomite mudstone, which are mainly with organic facies A-BC and are characterized by high ratios of HI, S/C, and Gam/C_30_H and low ratios of Pr/Ph. The source rocks in Aer sag are composed of mudstones, which are mainly with organic facies B-BC and are characterized by medium ratios of HI, S/C, Gam/C_30_H, Pr/Ph, and C_19_/C_23_TT. The source rocks in WY sag are composed of mudstone with organic facies BC-C and are characterized by low ratios of HI, S/C, and Gam/C_30_H and high ratios of Pr/Ph and C_19_/C_23_TT.
(2) Dolomitic mudstone is mainly distributed in the footwall blocks of boundary normal faults or in the sub-sag zone and is deposited in anoxic to dysoxic and saline to brackish environments, with biological sources originating from algae or bacteria and limited amounts of terrestrial organic matter. Mudstone with organic facies BC–C is widely distributed in the slope areas and is deposited in dysoxic to oxic and brackish to fresh environments, with biological sources originating from terrestrial organic matter and limited aquatic organic matter.
(3) The distribution and chemical composition of dolomitic mudstone was related to intensive volcanic or hydrothermal activity.
(4) The increase in sulfur content and ion concentrations of Ca, Mg, Cu, Zn, and Ni caused by hydrothermal or volcanic activity is likely the main reason for the formation of saline lakes with high primary productivity in the Erlian Basin.

## Supporting Information Available

Bulk organic geochemical data (PDF)
