# Supplementary material for: Paleoenvironment and Organic Characterization of the Lower Cretaceous Lacustrine Source Rocks in the Erlian Basin: The Influence of Hydrothermal and Volcanic Activity on the Source Rock Quality
Source: ACS Omega. 2023 Jan 3;8(2):1885–911. doi: 10.1021/acsomega.2c03487 (PMC9850471; doi:10.1021/acsomega.2c03487)
Supplement: Supplementary file 1 — ao2c03487_si_001.pdf [file ao2c03487_si_001.pdf]

Supporting information

Paleoenvironment and organic characterization of the Lower Cretaceous lacustrine source rocks in the Erlian Basin: The influence of hydrothermal and volcanic activity on source rock quality

Piao Wu[1,2], Dujie Hou[3], Lanzhu Cao[4], Ronghua Zheng[4], Xiuli Wei[4], Xiaoxiao Ma[3], Zhe Zhao[3], Jianwen Chen[1,2,*]

[1] Qingdao Institute of Marine Geology, Qingdao, 266237, China

[2] Laboratory for Marine Mineral Resources, Qingdao National Laboratory for Marine Science and Technology, Qingdao 266237, China

[3] School of Energy Resources, China University of Geosciences, Beijing 100083, China

[4] Research Institute of Petroleum Exploration and Development, PetroChina Huabei Oilfield Company, Renqiu 062550, China

[*] Corresponding author: Jianwen Chen (E-mail: jianwenchen2022@163.com)

Table S1. Rock-Eval pyrolysis data, total organic carbon and sulfur content in the source rocks from Member 4 of the Aershan Fm and Member 1 of the Tengger Fm in the BNAN, WLHs, Anan, Aer and WYs sags, Erlian Basin

NO	Sag/Zone	Well	Depth (m)	Strata	Lithology	Tmax(℃)	S_2(mg/g)	HI(mg/g)	TOC(%)	S(%)	S/C
1	BNAN sub-sag	B10	750	K_1bt_1	mudstone	425	12.30	368	3.34	0.62	0.19
2	BNAN sub-sag	B10	850	K_1bt_1	mudstone	426	11.60	441	2.63		
3	BNAN sub-sag	B10	975	K_1bt_1	mudstone	427	5.90	309	1.91		
4	BNAN sub-sag	B10	1125	K_1bt_1	dolomitic mudstone	431	18.20	639	2.82	0.75	0.27
5	BNAN sub-sag	B10	1187.5	K_1bt_1	dolomitic mudstone	433	39.32	750	5.24	1.16	0.22
6	BNAN sub-sag	B10	1188.6	K_1bt_1	dolomitic mudstone	435	38.04	809	4.70	0.44	0.09
7	BNAN sub-sag	B10	1250	K_1ba_4	dolomitic mudstone	429	36.38	723	5.03	0.72	0.14
8	BNAN sub-sag	B10	1259.4	K_1ba_4	dolomitic mudstone	432	23.06	682	3.38	0.87	0.26
9	BNAN sub-sag	B10	1375	K_1ba_4	dolomitic mudstone	429	12.86	649	1.98	0.82	0.41
10	BNAN sub-sag	B10	1525	K_1ba_4	dolomitic mudstone	433	8.36	449	1.86	0.32	0.17
11	BNAN sub-sag	B10	1675	K_1ba_4	dolomitic mudstone	434	17.26	649	2.66	0.53	0.20
12	BNAN sub-sag	B10	1775	K_1ba_4	mudstone	436	1.46	261	0.56	0.12	0.21
13	BNAN sub-sag	B66	1450	K_1ba_4	dolomitic mudstone	438	12.33	471	2.62	1.45	0.55
14	BNAN sub-sag	B66	1510	K_1ba_4	dolomitic mudstone	436	16.27	510	3.19	1.21	0.38
15	BNAN sub-sag	B66	1575	K_1ba_4	dolomitic mudstone	444	14.70	507	2.90	1.79	0.62
16	BNAN sub-sag	B66	1610	K_1ba_4	dolomitic mudstone	445	16.03	575	2.79	2.02	0.72
17	BNAN sub-sag	B66	1640	K_1ba_4	dolomitic mudstone	446	23.24	655	3.55	0.82	0.23
18	BNAN sub-sag	B66	1778	K_1ba_4	mudstone	444	5.23	331	1.58	0.78	0.49
19	BNAN sub-sag	B66	1869	K_1ba_4	mudstone	443	3.26	281	1.16	0.43	0.37
20	BNAN sub-sag	B66	1895	K_1ba_4	mudstone	444	3.09	276	1.12	0.80	0.71
21	BNAN sub-sag	B66	1905	K_1ba_4	mudstone	444	3.45	311	1.11	0.32	0.29
22	BNAN sub-sag	B66	1915	K_1ba_4	mudstone	443	2.23	214	1.04	0.17	0.16
23	BNAN sub-sag	B66	2195	K_1ba_4	mudstone	440	7.20	389	1.85	0.63	0.34
24	BNAN sub-sag	B66	2210	K_1ba_4	mudstone	441	5.49	323	1.70	0.97	0.57
25	BaI stepped belt	B48	1125	K_1ba_4	dolomitic mudstone	434	8.08	493	1.64	0.15	0.09
26	BaI stepped belt	B48	1250	K_1ba_4	dolomitic mudstone	437	14.32	573	2.50	0.40	0.16
27	BaI stepped belt	B48	1310	K_1ba_4	dolomitic mudstone	437	4.48	309	1.45	0.19	0.13
28	BaI stepped belt	B50	1346.3	K_1ba_4	dolomitic mudstone	430	9.00	581	1.55	0.20	0.13
29	BaI stepped belt	B50	1438	K_1ba_4	mudstone	435	6.96	446	1.56	0.64	0.41
30	BaI stepped belt	B50	1630	K_1ba_4	mudstone	435	7.08	478	1.48	0.47	0.32
31	BaI stepped belt	B5	1135	K_1ba_4	dolomitic mudstone	430	16.12	683	2.36	1.92	0.81
32	BaI stepped belt	B5	1182.5	K_1ba_4	dolomitic mudstone	433	15.17	762	1.99	0.80	0.40
33	BaI stepped belt	B5	1208.2	K_1ba_4	dolomitic mudstone	439	23.31	711	3.28	1.84	0.56
34	BaI stepped belt	B5	1240	K_1ba_4	dolomitic mudstone	430	4.28	563	0.76	1.67	2.20
35	BaI stepped belt	B5	1265.3	K_1ba_4	mudstone	437	2.26	279	0.81	1.32	1.63
36	BaI stepped belt	B5	1295	K_1ba_4	dolomitic mudstone	439	20.38	708	2.88	0.76	0.26
37	BaI stepped belt	B5	1325	K_1ba_4	dolomitic mudstone	439	20.30	707	2.87	3.43	1.20
38	BaI stepped belt	B5	1388	K_1ba_4	mudstone	435	13.78	607	2.27	1.08	0.48
39	BaI stepped belt	B5	1445	K_1ba_4	mudstone	440	11.43	608	1.88	1.87	0.99

40	BaI stepped belt	B5	1519	K_1ba_4	dolomitic mudstone	438	8.56	507	1.69	1.29	0.76
41	BaI stepped belt	B5	1480	K_1ba_4	mudstone	435	18.92	650	2.91	1.56	0.54
42	BaII stepped belt	B19	1441.9	K_1ba_4	dolomitic mudstone	436	20.26	536	3.78		
43	BaII stepped belt	B19	1503.7	K_1ba_4	mudstone	438	10.32	558	1.85	0.18	0.10
44	BaII stepped belt	B19	1692.5	K_1ba_4	mudstone	435	7.06	480	1.47	0.46	0.31
45	BaII stepped belt	B19	1772.5	K_1ba_4	mudstone	433	4.24	389	1.09	0.37	0.34
46	BaII stepped belt	B2	1315	K_1ba_4	dolomitic mudstone	431	9.52	577	1.65	0.23	0.14
47	BaII stepped belt	B2	1348.5	K_1ba_4	dolomitic mudstone	434	11.37	552	2.06	0.16	0.08
48	BaII stepped belt	B2	1443	K_1ba_4	mudstone	435	3.69	456	0.81	0.19	0.23
49	BaII stepped belt	B35	1763	K_1ba_4	mudstone	441	6.64	332	2.00	0.25	0.13
50	BaII stepped belt	B35	1872.5	K_1ba_4	mudstone	440	7.82	348	2.25	0.53	0.24
51	BaII stepped belt	B35	1949.1	K_1ba_4	mudstone	435	2.45	253	0.97	0.41	0.42
52	Honggeer uplift	L24X	980	K_1bt_1	mudstone	429	10.06	538	1.87	0.34	0.18
53	Honggeer uplift	L24X	1050	K_1bt_1	mudstone	430	20.75	659	3.15	0.32	0.10
54	Honggeer uplift	L24X	1075	K_1bt_1	mudstone	432	1.90	214	0.89	0.35	0.40
55	Honggeer uplift	L24X	1105.5	K_1bt_1	dolomitic mudstone	432	6.56	443	1.48	0.32	0.21
56	Honggeer uplift	L24X	1175.0	K_1bt_1	dolomitic mudstone	434	13.18	588	2.24	0.30	0.13
57	Honggeer uplift	L24X	1245	K_1bt_1	dolomitic mudstone	433	7.10	473	1.50	0.34	0.22
58	Honggeer uplift	L24X	1275	K_1bt_1	dolomitic mudstone	434	18.61	644	2.89	0.40	0.14
59	Honggeer uplift	L24X	1317	K_1bt_1	dolomitic mudstone	435	17.72	633	2.80	0.38	0.13
60	Honggeer uplift	L24X	1335	K_1bt_1	mudstone	436	14.69	595	2.47	0.25	0.10
61	Honggeer uplift	L24X	1375	K_1bt_1	dolomitic mudstone	436	5.88	426	1.38	0.34	0.24
62	Honggeer uplift	L24X	1420	K_1bt_1	dolomitic mudstone	435	15.15	609	2.49	0.27	0.11
63	Honggeer uplift	L24X	1480	K_1ba_4	dolomitic mudstone	436	9.25	523	1.77	0.64	0.36
64	Honggeer uplift	L24X	1505	K_1ba_4	dolomitic mudstone	438	5.60	415	1.35	0.44	0.33
65	Honggeer uplift	L24X	1599	K_1ba_4	dolomitic mudstone	439	10.53	543	1.94	0.62	0.32
66	Honggeer uplift	L24X	1690	K_1ba_4	dolomitic mudstone	442	20.75	648	3.20	1.21	0.38
67	Honggeer uplift	L24X	1850	K_1ba_4	dolomitic mudstone	446	15.86	606	2.62	1.11	0.42
68	Honggeer uplift	L24X	1738	K_1ba_4	dolomitic mudstone	444	12.36	570	2.17	0.90	0.42
69	Honggeer uplift	L24X	1779	K_1ba_4	dolomitic mudstone	443	8.52	495	1.72	0.66	0.38
70	Honggeer uplift	L24X	1920	K_1ba_4	mudstone	447	12.96	581	2.23	0.64	0.29
71	Hongjing uplift	L4	1274	K_1bt_1	mudstone	438	12.42	380	3.27	1.20	0.37
72	Hongjing uplift	L4	1358	K_1bt_1	mudstone	438	4.62	238	1.94	0.41	0.21
73	Hongjing uplift	L4	1450	K_1bt_1	mudstone	437	5.80	308	1.88	0.45	0.24
74	Hongjing uplift	L4	1490	K_1bt_1	mudstone	441	4.30	262	1.64	0.44	0.27
75	Hongjing uplift	L4	1534	K_1bt_1	mudstone	440	3.31	203	1.63	0.34	0.21
76	Hongjing uplift	L4	1570	K_1bt_1	mudstone	441	3.74	231	1.62	0.39	0.24
77	Hongjing uplift	L4	1608	K_1bt_1	mudstone	439	10.75	354	3.04	0.79	0.26
78	Hongjing uplift	L4	1622	K_1bt_1	mudstone	441	8.39	365	2.30	0.28	0.12
79	Hongjing uplift	L4	1686	K_1bt_1	mudstone	439	3.44	232	1.48	0.35	0.24
80	Hongjing uplift	L4	1725	K_1bt_1	mudstone	440	14.17	419	3.38	0.45	0.13
81	Hongjing uplift	L4	1800	K_1ba_4	dolomitic mudstone	449	7.63	422	1.81	0.96	0.53
82	Hongjing uplift	L4	1830	K_1ba_4	mudstone	439	6.44	312	2.06	0.79	0.39
83	Hongjing uplift	L4	1849	K_1ba_4	mudstone	449	3.61	304	1.19	0.23	0.19

84	Saiwusu uplift	L12X	1794.5	K_1ba_4	mudstone	442	7.40	448	1.65	0.48	0.29
85	Saiwusu uplift	L12X	1825	K_1ba_4	mudstone	445	14.45	486	2.97	0.54	0.18
86	Saiwusu uplift	L12X	1865	K_1ba_4	mudstone	446	10.94	488	2.24	0.87	0.39
87	Saiwusu uplift	L12X	1905	K_1ba_4	mudstone	447	4.78	325	1.47	0.49	0.33
88	Saiwusu uplift	L12X	1935	K_1ba_4	mudstone	442	6.03	328	1.84	0.49	0.27
89	Saiwusu uplift	L12X	1963	K_1ba_4	mudstone	444	4.70	324	1.45	0.48	0.33
90	Saiwusu uplift	L12X	2025.5	K_1ba_4	mudstone	447	4.77	304	1.57	0.27	0.17
91	Saiwusu uplift	L12X	2059	K_1ba_4	mudstone	446	2.97	220	1.35	0.31	0.23
92	Saiwusu uplift	L12X	2171.5	K_1ba_4	mudstone	446	4.17	280	1.49	0.35	0.23
93	Saiwusu uplift	L12X	2207	K_1ba_4	mudstone	446	6.83	321	2.13	0.42	0.19
94	Saiwusu uplift	L12X	2277	K_1ba_4	mudstone	448	5.35	190	2.82	1.01	0.36
95	Saiwusu uplift	L12X	2297	K_1ba_4	mudstone	445	2.82	144	1.96	0.99	0.50
96	Saiwusu uplift	L12X	2317	K_1ba_4	mudstone	453	9.30	186	5.00	1.22	0.24
97	Saiwusu uplift	L12X	2325	K_1ba_4	mudstone	454	14.59	278	5.24	0.92	0.17
98	Saiwusu uplift	L1	1261	K_1bt_1	mudstone	439	1.04	120	0.87	0.05	0.06
99	Saiwusu uplift	L1	1331	K_1bt_1	mudstone	434	0.40	77	0.52	0.02	0.04
100	Saiwusu uplift	L1	1463	K_1bt_1	mudstone	437	0.80	129	0.62	0.03	0.05
101	Saiwusu uplift	L1	1775	K_1bt_1	mudstone	447	3.30	374	0.88	0.24	0.27
102	Saiwusu uplift	L1	1825	K_1ba_4	mudstone	448	5.60	357	1.57	0.24	0.15
103	Saiwusu uplift	L1	1875	K_1ba_4	mudstone	447	8.18	454	1.80	0.28	0.15
104	Saiwusu uplift	L1	1935	K_1ba_4	mudstone	449	6.21	457	1.36	0.30	0.22
105	Saiwusu uplift	L1	2025	K_1ba_4	mudstone	450	2.12	286	0.74	0.14	0.19
106	Saiwusu uplift	L1	2095	K_1ba_4	mudstone	450	4.47	385	1.16	0.22	0.19
107	Saiwusu uplift	L1	2195	K_1ba_4	mudstone	455	14.31	511	2.80	0.38	0.14
108	WLHs sub-sag	LD1	1520	K_1bt_1	mudstone	444	3.26	228	1.43	0.13	0.09
109	WLHs sub-sag	LD1	1548	K_1bt_1	mudstone	442	3.66	244	1.50	0.10	0.06
110	WLHs sub-sag	LD1	1570	K_1bt_1	mudstone	446	3.93	258	1.52	0.14	0.09
111	WLHs sub-sag	LD1	1590	K_1bt_1	mudstone	440	1.24	149	0.83	0.13	0.15
112	WLHs sub-sag	LD1	1609.8	K_1bt_1	mudstone	443	0.65	94	0.69	0.05	0.07
113	WLHs sub-sag	LD1	1623	K_1bt_1	mudstone	442	1.41	168	0.84	0.20	0.24
114	WLHs sub-sag	LD1	1641	K_1bt_1	mudstone	439	1.21	147	0.82	0.17	0.20
115	WLHs sub-sag	LD1	1660	K_1bt_1	mudstone	446	3.06	220	1.39	0.14	0.10
116	WLHs sub-sag	LD1	1670	K_1bt_1	mudstone	444	5.04	357	1.41	0.30	0.21
117	WLHs sub-sag	LD1	1685	K_1bt_1	mudstone	449	3.70	259	1.43	0.10	0.07
118	WLHs sub-sag	LD1	1708	K_1bt_1	mudstone	446	4.07	306	1.33	0.16	0.12
119	WLHs sub-sag	LD1	1721	K_1bt_1	mudstone	443	5.89	385	1.53	0.22	0.14
120	WLHs sub-sag	LD1	1734	K_1bt_1	mudstone	445	2.75	227	1.21	0.17	0.14
121	WLHs sub-sag	LD1	1749	K_1bt_1	mudstone	444	8.17	452	1.81	0.36	0.20
122	WLHs sub-sag	LD1	1762.3	K_1bt_1	mudstone	448	6.41	279	2.30	0.82	0.36
123	WLHs sub-sag	LD1	1775	K_1bt_1	mudstone	443	2.86	275	1.04	0.32	0.31
124	WLHs sub-sag	LD1	1790	K_1bt_1	mudstone	451	11.21	485	2.31	0.24	0.11
125	WLHs sub-sag	LD1	1823	K_1bt_1	dolomitic mudstone	447	14.72	691	2.13	0.36	0.17
126	WLHs sub-sag	LD1	1830	K_1bt_1	dolomitic mudstone	448	8.90	422	2.11	1.98	0.94
127	WLHs sub-sag	LD1	1844	K_1bt_1	dolomitic mudstone	449	21.65	779	2.78	0.10	0.04

128	WLHs sub-sag	LD1	1860	K_1bt_1	dolomitic mudstone	453	6.67	433	1.54	0.10	0.07
129	WLHs sub-sag	LD1	1883	K_1bt_1	dolomitic mudstone	448	8.55	467	1.83	0.06	0.03
130	WLHs sub-sag	LD1	1923	K_1bt_1	mudstone	453	11.37	507	2.24	0.26	0.11
131	WLHs sub-sag	LD1	1948	K_1bt_1	dolomitic mudstone	447	10.22	473	2.16	0.33	0.15
132	WLHs sub-sag	LD1	1973	K_1ba_4	dolomitic mudstone	453	10.05	513	1.96	0.33	0.17
133	WLHs sub-sag	LD1	1990	K_1ba_4	dolomitic mudstone	454	13.37	546	2.45	1.32	0.54
134	WLHs sub-sag	LD1	2000	K_1ba_4	mudstone	448	9.63	515	1.87	0.69	0.37
135	WLHs sub-sag	LD1	2015	K_1ba_4	mudstone	446	8.27	394	2.10	0.44	0.21
136	WLHs sub-sag	LD1	2030	K_1ba_4	mudstone	450	14.61	497	2.94	0.85	0.29
137	WLHs sub-sag	LD1	2047	K_1ba_4	mudstone	448	4.30	274	1.57	0.13	0.09
138	WLHs sub-sag	LD1	2065.3	K_1ba_4	mudstone	447	3.18	199	1.60	0.61	0.38
139	WLHs sub-sag	LD1	2070	K_1ba_4	mudstone	453	7.48	365	2.05	0.26	0.13
140	WLHs sub-sag	LD1	2095	K_1ba_4	mudstone	453	8.06	407	1.98	0.38	0.19
141	WLHs sub-sag	LD1	2120	K_1ba_4	mudstone	452	2.37	253	0.94	0.22	0.23
142	WLHs sub-sag	LD1	2145	K_1ba_4	mudstone	455	4.57	322	1.42	0.20	0.14
143	WLHs sub-sag	LD1	2155	K_1ba_4	mudstone	453	3.48	290	1.20	0.45	0.37
144	WLHs sub-sag	LD1	2174	K_1ba_4	mudstone	451	1.13	190	0.60	0.20	0.34
145	WLHs sub-sag	LD1	2198	K_1ba_4	mudstone	451	3.12	262	1.19	0.24	0.20
146	WLHs sub-sag	LD1	2222	K_1ba_4	mudstone	453	1.25	161	0.78	0.12	0.16
147	WLHs sub-sag	LD1	2245	K_1ba_4	mudstone	450	3.63	271	1.34	0.20	0.15
148	WLHs sub-sag	LD1	2275	K_1ba_4	mudstone	453	3.37	301	1.12	0.39	0.35
149	WLHs sub-sag	LD1	2300	K_1ba_4	mudstone	453	3.90	289	1.35	0.30	0.22
150	WLHs sub-sag	LD1	2320	K_1ba_4	mudstone	455	9.65	351	2.75	0.92	0.34
151	WLHs sub-sag	LD1	2330	K_1ba_4	dolomitic mudstone	457	14.22	496	2.87	1.31	0.46
152	WLHs sub-sag	LD1	2355	K_1ba_4	mudstone	456	4.19	234	1.79	0.28	0.15
153	WLHs sub-sag	LD1	2380	K_1ba_4	dolomitic mudstone	451	2.60	232	1.12	0.27	0.24
154	WLHs sub-sag	LD1	2390	K_1ba_4	dolomitic mudstone	451	2.50	202	1.24	0.54	0.43
155	WLHs sub-sag	LD1	2400	K_1ba_4	dolomitic mudstone	456	2.14	163	1.31	0.16	0.12
156	Tumuer uplift	L15X	1110	K_1bt_1	mudstone	436	5.97	244	2.45	0.60	0.24
157	Tumuer uplift	L15X	1212	K_1bt_1	mudstone	437	4.17	218	1.91	0.47	0.24
158	Tumuer uplift	L15X	1274	K_1bt_1	mudstone	437	5.10	252	2.02	0.29	0.14
159	Tumuer uplift	L15X	6333	K_1bt_1	mudstone	438	8.85	357	2.48	0.65	0.26
160	Tumuer uplift	L15X	1425	K_1bt_1	mudstone	430	9.89	416	2.38	0.45	0.19
161	Tumuer uplift	L15X	1400	K_1bt_1	mudstone	436	9.26	325	2.85	0.77	0.27
162	Tumuer uplift	L15X	1495	K_1bt_1	mudstone	432	11.57	348	3.33	0.71	0.21
163	Tumuer uplift	L15X	1535	K_1bt_1	mudstone	437	8.24	387	2.13	0.50	0.23
164	Tumuer uplift	L15X	1593.1	K_1bt_1	mudstone	442	17.21	548	3.14	0.88	0.28
165	Tumuer uplift	L15X	1680	K_1ba_4	dolomitic mudstone	440	12.48	543	2.30	0.25	0.11
166	Tumuer uplift	L15X	1710	K_1ba_4	dolomitic mudstone	440	14.10	665	2.12	0.38	0.18
167	Tumuer uplift	L15X	1730	K_1ba_4	dolomitic mudstone	436	13.54	651	2.08	0.29	0.14
168	Tumuer uplift	L15X	1795	K_1ba_4	dolomitic mudstone	442	12.43	573	2.17	0.60	0.28
169	Tumuer uplift	L15X	1865	K_1ba_4	dolomitic mudstone	444	15.01	573	2.62	0.83	0.32
170	Tumuer uplift	L15X	1954	K_1ba_4	mudstone	441	16.64	514	3.24	0.64	0.20
171	Tumuer uplift	L2	917	K_1bt_1	mudstone	433	4.83	261	1.85	0.85	0.46

172	Tumuer uplift	L2	966	K_1bt_1	mudstone	433	6.26	278	2.25	0.58	0.26
173	Tumuer uplift	L2	982	K_1bt_1	mudstone	435	6.74	288	2.34	0.66	0.28
174	Tumuer uplift	L2	1006	K_1bt_1	mudstone	435	7.39	344	2.15	0.51	0.24
175	Tumuer uplift	L2	1025	K_1bt_1	mudstone	432	9.67	438	2.21	0.53	0.24
176	Tumuer uplift	L2	1055	K_1bt_1	mudstone	435	8.87	435	2.04	0.65	0.32
177	Tumuer uplift	L2	1070	K_1bt_1	mudstone	438	9.29	449	2.07	1.19	0.57
178	Tumuer uplift	L2	1090	K_1bt_1	mudstone	437	6.34	360	1.76	0.79	0.45
179	Tumuer uplift	L2	1108	K_1bt_1	mudstone	437	4.99	337	1.48	0.46	0.31
180	Tumuer uplift	L2	1122	K_1bt_1	mudstone	438	4.12	288	1.43	0.85	0.59
181	Tumuer uplift	L2	1135	K_1bt_1	mudstone	436	4.86	306	1.59	0.75	0.47
182	Tumuer uplift	L2	1157	K_1bt_1	mudstone	436	11.13	508	2.19	1.42	0.65
183	Tumuer uplift	L2	1172	K_1bt_1	dolomitic mudstone	437	10.10	497	2.03	1.87	0.92
184	Tumuer uplift	L2	1186	K_1bt_1	dolomitic mudstone	438	12.42	557	2.23	1.22	0.55
185	Tumuer uplift	L2	1210	K_1bt_1	mudstone	442	9.05	469	1.93	0.80	0.41
186	Tumuer uplift	L2	1235	K_1bt_1	mudstone	438	11.30	483	2.34	0.95	0.40
187	Tumuer uplift	L2	1266	K_1bt_1	mudstone	441	11.83	556	2.13	1.00	0.47
188	Tumuer uplift	L2	1295	K_1bt_1	mudstone	441	7.92	440	1.80	0.94	0.52
189	Tumuer uplift	L2	1315	K_1bt_1	mudstone	440	5.08	332	1.53	0.72	0.47
190	Tumuer uplift	L2	1350	K_1ba_4	mudstone	438	8.17	423	1.93	1.08	0.56
191	Tumuer uplift	L2	1373	K_1ba_4	mudstone	440	9.17	449	2.04	0.97	0.47
192	Tumuer uplift	L2	1391	K_1ba_4	mudstone	443	10.32	532	1.94	1.01	0.52
193	Tumuer uplift	L2	1410	K_1ba_4	mudstone	444	6.70	392	1.71	0.82	0.48
194	Anan anticline	AM2	965	K_1bt_1	mudstone	434	5.86	298	1.97	0.23	0.12
195	Anan anticline	AM2	1100	K_1bt_1	mudstone	443	7.93	419	1.89	0.25	0.13
196	Anan anticline	AM2	1260	K_1bt_1	mudstone	440	9.74	475	2.05	0.29	0.14
197	Anan anticline	AM2	1400	K_1bt_1	dolomitic mudstone	443	5.83	371	1.57	0.26	0.16
198	Anan anticline	AM2	1430	K_1bt_1	dolomitic mudstone	447	7.30	437	1.67	0.28	0.17
199	Anan anticline	AM2	1470	K_1bt_1	dolomitic mudstone	444	8.47	460	1.84	0.28	0.15
200	Anan anticline	AM2	1510	K_1bt_1	dolomitic mudstone	451	13.18	602	2.19	0.31	0.14
201	Anan anticline	AM2	1543.6	K_1bt_1	mudstone	439	27.31	676	4.04	0.17	0.04
202	Anan anticline	AM2	1547.4	K_1bt_1	dolomitic mudstone	443	19.82	642	3.09	0.35	0.11
203	Anan anticline	AM2	1551.3	K_1bt_1	dolomitic mudstone	450	11.07	612	1.81	0.37	0.20
204	Anan anticline	AM2	1557	K_1bt_1	dolomitic mudstone	448	19.80	680	2.91	0.14	0.05
205	Anan anticline	AM2	1558.9	K_1bt_1	dolomitic siltstone	443	18.20	660	2.76	0.56	0.20
206	Anan anticline	AM2	1562.6	K_1bt_1	dolomitic siltstone	442	18.74	586	3.20	0.18	0.06
207	Anan anticline	AM2	1570.6	K_1bt_1	mudstone	441	11.92	590	2.02	0.35	0.17
208	Anan anticline	AM2	1575.2	K_1bt_1	dolomitic siltstone	451	13.25	518	2.56	0.21	0.08
209	Anan anticline	AM2	1588.1	K_1bt_1	dolomitic siltstone	450	13.00	530	2.45	0.50	0.20
210	Anan anticline	AM2	1606.2	K_1bt_1	mudstone	450	0.80	124	0.64	0.09	0.14
211	Anan anticline	AM2	1609.2	K_1bt_1	mudstone	449	1.17	169	0.69	0.06	0.08
212	Anan anticline	AM2	1647	K_1ba_4	dolomitic siltstone	444	8.30	474	1.75	0.31	0.18
213	Anan anticline	AM2	1707	K_1ba_4	mudstone	444	5.72	418	1.37	0.29	0.21
214	Anan anticline	A3	1163	K_1bt_1	mudstone	436	4.85	237	2.04	0.19	0.09
215	Anan anticline	A3	1190	K_1bt_1	mudstone	433	5.83	296	1.98	0.25	0.13

216	Anan anticline	A3	1225	K_1bt_1	mudstone	436	4.83	395	1.22	0.31	0.25
217	Anan anticline	A3	1304.7	K_1bt_1	mudstone	442	12.45	419	2.97	0.72	0.24
218	Anan anticline	A3	1335	K_1bt_1	mudstone	437	6.89	334	2.06	0.25	0.12
219	Anan anticline	A3	1368	K_1bt_1	mudstone	441	13.38	519	2.58	0.32	0.12
220	Anan anticline	A3	1450	K_1ba_4	mudstone	437	7.57	354	2.14	0.30	0.14
221	Anan anticline	A35	1597.2	K_1bt_1	mudstone	444	19.49	725	2.69	0.06	0.02
222	Anan anticline	A35	1605.3	K_1bt_1	mudstone	446	18.27	700	2.61	0.72	0.28
223	Anan anticline	A35	1616.9	K_1bt_1	mudstone	445	22.94	728	3.15	1.06	0.34
224	Anan anticline	A35	1612.7	K_1bt_1	mudstone	443	12.41	599	2.07	0.06	0.03
225	Anan anticline	A35	1620.3	K_1bt_1	mudstone	447	34.57	860	4.02	1.30	0.32
226	Anan anticline	A35	1625.8	K_1bt_1	mudstone	445	15.06	707	2.13	0.65	0.31
227	Hanan anticline	H1	1027.5	K_1bt_1	mudstone	433	10.29	390	2.64	0.36	0.14
228	Hanan anticline	H1	1080.5	K_1bt_1	mudstone	430	4.25	177	2.40	0.18	0.08
229	Hanan anticline	H1	1132.5	K_1bt_1	mudstone	436	4.10	185	2.22	0.15	0.07
230	Hanan anticline	H1	1192.5	K_1bt_1	mudstone	433	9.17	294	3.12	0.23	0.07
231	Hanan anticline	H1	1230	K_1bt_1	mudstone	440	4.33	321	1.35	0.16	0.12
232	Hanan anticline	H1	1307.5	K_1ba_4	mudstone	434	8.73	272	3.21	0.23	0.07
233	Hanan anticline	H1	1360	K_1ba_4	mudstone	435	8.17	303	2.70	0.39	0.14
234	Hanan anticline	H1	1421.8	K_1ba_4	mudstone	439	7.76	443	1.75	0.25	0.14
235	Hanan anticline	H1	1480	K_1ba_4	mudstone	437	8.48	353	2.40	0.28	0.12
236	Hanan anticline	H1	1510.5	K_1ba_4	mudstone	437	9.57	378	2.53	0.32	0.13
237	Hanan anticline	H8	1228.6	K_1ba_4	dolomitic siltstone	437	10.10	765	1.32	0.10	0.08
238	Hanan anticline	H8	1469.6	K_1ba_4	mudstone	440	8.85	268	3.30	0.16	0.05
239	Hanan anticline	H8	1660	K_1ba_4	mudstone	439	3.60	621	0.58	0.15	0.26
240	Anan slope	H68	1652	K_1bt_1	mudstone	435	3.94	116	3.40	0.10	0.03
241	Anan slope	H68	1767	K_1bt_1	mudstone	435	2.26	121	1.87	0.09	0.05
242	Anan slope	H68	1715	K_1bt_1	mudstone	433	2.17	146	1.48	0.12	0.08
243	Anan slope	H68	1690	K_1bt_1	mudstone	436	2.84	170	1.67	0.18	0.11
244	Anan slope	H68	1796	K_1bt_1	mudstone	431	2.74	171	1.60	0.16	0.10
245	Anan slope	H68	1824	K_1ba_4	mudstone	435	3.73	200	1.86	0.17	0.09
246	Anan slope	H68	1933	K_1ba_4	mudstone	439	2.71	196	1.38	0.20	0.14
247	Anan slope	H68	1947	K_1ba_4	mudstone	436	2.98	187	1.59	0.24	0.15
248	Anan slope	H68	1975	K_1ba_4	mudstone	434	3.31	224	1.48	0.29	0.20
249	Anan slope	H68	2000	K_1ba_4	mudstone	436	2.85	224	1.27	0.46	0.36
250	Anan sub-sag	H20	2165	K_1bt_1	mudstone	437	4.26	282	1.51	0.31	0.21
251	Anan sub-sag	H20	2199.7	K_1ba_4	dolomitic siltstone	436	4.53	343	1.32	0.24	0.18
252	Anan sub-sag	H20	2295.1	K_1ba_4	mudstone	445	6.40	239	2.68	0.20	0.07
253	Anan sub-sag	H20	2366	K_1ba_4	mudstone	437	6.31	345	1.83	0.28	0.15
254	Anan sub-sag	H20	2419	K_1ba_4	mudstone	437	4.87	323	1.51	0.31	0.21
255	Anan sub-sag	H20	2479	K_1ba_4	mudstone	438	4.41	288	1.53	0.37	0.24
256	Anan sub-sag	H20	2559	K_1ba_4	mudstone	441	5.77	352	1.64	0.34	0.21
257	Anan sub-sag	H20	2664	K_1ba_4	mudstone	439	3.33	264	1.26	0.24	0.19
258	Anan sub-sag	H20	2696	K_1ba_4	mudstone	437	3.10	274	1.13	0.16	0.14
259	Anan sub-sag	H20	2731	K_1ba_4	mudstone	439	4.48	350	1.28	0.22	0.17

260	Anan sub-sag	A43	2034.1	K_1bt_1	dolomitic siltstone	444	9.83	473	2.08	0.14	0.07
261	Anan sub-sag	A43	2062.2	K_1bt_1	dolomitic siltstone	448	7.30	541	1.35	0.41	0.30
262	Anan sub-sag	A47	2004.7	K_1bt_1	dolomitic siltstone	448	17.77	651	2.73	0.37	0.13
263	Anan sub-sag	A47	2014.2	K_1bt_1	dolomitic siltstone	449	35.72	867	4.12		
264	Anan sub-sag	A47	2034.2	K_1bt_1	dolomitic siltstone	444	30.16	783	3.85		
265	Anan sub-sag	A47	2077.4	K_1bt_1	dolomitic siltstone	441	27.17	688	3.95		
266	Anan sub-sag	A47	2125.4	K_1bt_1	dolomitic siltstone	443	16.22	651	2.49	0.70	0.28
267	Anan sub-sag	A47	2130.1	K_1bt_1	dolomitic siltstone	446	23.15	764	3.03	0.27	0.09
268	Anan sub-sag	A47	2133.1	K_1bt_1	dolomitic siltstone	446	15.00	758	1.98	0.69	0.35
269	Aer central sub-sag	Aer13	1715	K_1bt_1	mudstone	442	20.78	536	3.88	1.27	0.33
270	Aer central sub-sag	Aer13	1765	K_1bt_1	mudstone	441	21.76	676	3.22	0.32	0.10
271	Aer central sub-sag	Aer13	1785	K_1bt_1	mudstone	436	20.05	641	3.13	1.02	0.33
272	Aer central sub-sag	Aer13	1813	K_1bt_1	mudstone	441	9.33	400	2.33	0.95	0.41
273	Aer central sub-sag	Aer13	1830	K_1bt_1	mudstone	436	12.66	432	2.93	0.59	0.20
274	Aer central sub-sag	Aer13	1870	K_1bt_1	mudstone	441	3.81	266	1.43	0.14	0.10
275	Aer central sub-sag	Aer13	1895	K_1bt_1	mudstone	437	8.31	536	1.55	0.23	0.15
276	Aer central sub-sag	Aer13	1925	K_1bt_1	mudstone	437	7.80	429	1.82	0.16	0.09
277	Aer central sub-sag	Aer13	1970	K_1bt_1	mudstone	445	3.53	315	1.12	0.24	0.21
278	Aer central sub-sag	Aer13	2015	K_1bt_1	mudstone	438	12.98	519	2.50	0.69	0.28
279	Aer central sub-sag	Aer13	2050	K_1bt_1	mudstone	435	8.38	487	1.72	0.65	0.38
280	Aer central anticline	Aer1	1466.1	K_1bt_1	mudstone	429	29.51	886	3.33	0.91	0.27
281	Aer central anticline	Aer1	1467.1	K_1bt_1	mudstone	426	21.41	601	3.56	1.60	0.45
282	Aer central anticline	Aer1	1467.5	K_1bt_1	mudstone	428	30.67	743	4.13	0.08	0.02
283	Aer central anticline	Aer1	1467.8	K_1bt_1	mudstone	425	41.17	745	5.53	0.16	0.03
284	Aer central anticline	Aer1	1494	K_1bt_1	mudstone	430	8.53	523	1.63	0.42	0.26
285	Aer central anticline	Aer1	1510	K_1bt_1	mudstone	430	11.83	514	2.30	0.82	0.35
286	Aer central anticline	Aer1	1530	K_1bt_1	mudstone	434	12.47	540	2.31	0.41	0.18
287	Aer central anticline	Aer1	1545	K_1bt_1	mudstone	430	13.21	582	2.27	0.83	0.37
288	Aer central anticline	Aer1	1566	K_1bt_1	mudstone	430	8.62	513	1.68		
289	Aer central anticline	Aer1	1595	K_1bt_1	mudstone	433	14.34	624	2.30	1.06	0.46
290	Aer central anticline	Aer1	1620	K_1bt_1	mudstone	434	15.18	625	2.43	0.55	0.23
291	Aer central anticline	Aer1	1645	K_1bt_1	mudstone	435	10.76	558	1.93	0.42	0.22
292	Aer central anticline	Aer1	1675	K_1bt_1	mudstone	430	5.62	480	1.17	0.16	0.14
293	Aer central anticline	Aer1	1775	K_1bt_1	mudstone	434	4.50	405	1.11	0.05	0.05
294	Aer central anticline	Aer1	1804	K_1bt_1	mudstone	442	4.13	378	1.09	0.04	0.04
295	Aer central anticline	Aer1	1816	K_1bt_1	mudstone	437	3.84	346	1.11	0.03	0.03
296	Aer central anticline	Aer1	1835	K_1bt_1	mudstone	439	4.27	365	1.17	0.06	0.05
297	Aer central anticline	Aer1	1990	K_1ba_4	mudstone	432	3.96	363	1.09	0.03	0.03
298	Aer central anticline	Aer1	2165	K_1ba_4	mudstone	432	10.89	499	2.18	0.56	0.26
299	Aer central anticline	Aer1	2375	K_1ba_4	mudstone	433	5.89	487	1.21	0.09	0.07
300	Aer western slope	Aer62	1710	K_1bt_1	mudstone	442	17.16	468	3.67	0.70	0.19
301	Aer western slope	Aer62	1735	K_1bt_1	mudstone	434	5.84	169	3.45	0.08	0.02
302	Aer western slope	Aer62	1770	K_1bt_1	mudstone	440	9.55	363	2.63	0.44	0.17
303	Aer western slope	Aer62	1790	K_1bt_1	mudstone	447	10.35	389	2.66	0.89	0.33

304	Aer western slope	Aer62	1825	K_1bt_1	mudstone	430	4.32	210	2.06	0.11	0.05
305	Aer western slope	Aer62	1860	K_1bt_1	mudstone	440	5.70	303	1.88	0.29	0.15
306	Aer western slope	Aer62	1880	K_1bt_1	mudstone	445	5.39	291	1.85	0.48	0.26
307	Aer western slope	Aer62	1905	K_1bt_1	mudstone	440	4.02	222	1.81	0.04	0.02
308	Aer western slope	Aer62	1930	K_1bt_1	mudstone	440	4.78	258	1.85	0.14	0.08
309	Aer western slope	Aer62	1960	K_1bt_1	mudstone	439	3.84	191	2.01	0.06	0.03
310	Aer western slope	Aer62	1990	K_1bt_1	mudstone	440	2.78	230	1.21	0.07	0.06
311	Aer western slope	Aer62	2015	K_1bt_1	mudstone	442	1.49	186	0.80	0.03	0.04
312	Aer western slope	Aer62	2087	K_1ba_4	mudstone	440	3.72	332	1.12	0.17	0.15
313	Aer western slope	Aer62	2112	K_1ba_4	mudstone	440	2.36	205	1.15	0.18	0.16
314	Aer western slope	Aer62	2140	K_1ba_4	mudstone	441	3.60	243	1.48	0.17	0.11
315	Aer eastern slope	Aer7	1566	K_1bt_1	mudstone	430	2.76	208	1.33	0.09	0.07
316	Aer eastern slope	Aer7	1161	K_1bt_1	mudstone	433	2.34	128	1.83	0.24	0.13
317	Aer eastern slope	Aer7	1334	K_1bt_1	mudstone	438	2.09	213	0.98	0.20	0.20
318	Aer eastern slope	Aer7	1409	K_1bt_1	mudstone	437	2.71	352	0.77	0.14	0.18
319	WYs sub-sag	T11	2186	K_1bt_1	mudstone	445	3.78	165	2.29		
320	WYs sub-sag	T11	2167	K_1bt_1	mudstone	440	3.33	163	2.04		
321	WYs sub-sag	T11	2275	K_1bt_1	mudstone	441	3.78	160	2.36		
322	WYs sub-sag	T11	2242	K_1bt_1	mudstone	441	4.17	154	2.70		
323	WYs sub-sag	T11	2140	K_1bt_1	mudstone	441	3.99	148	2.70		
324	WYs sub-sag	T11	2215	K_1bt_1	mudstone	445	3.78	137	2.75		
325	WYs sub-sag	T11	2305	K_1bt_1	mudstone	445	4.75	129	3.69		
326	Eastern slope	T17	2240.5	K_1bt_1	mudstone	447	7.83	300	2.61		
327	Eastern slope	T17	2243.4	K_1bt_1	mudstone	455	4.68	233	2.01		
328	Eastern slope	T17	2073	K_1bt_1	mudstone	445	8.26	227	3.63		
329	Eastern slope	T17	2063.8	K_1bt_1	mudstone	445	3.43	172	1.99		
330	Eastern slope	T17	1905.6	K_1bt_1	mudstone	439	3.35	107	3.12		
331	Eastern slope	T27	1685	K_1ba_4	mudstone	438	8.38	257	3.26		
332	Eastern slope	T27	1815	K_1bt_1	mudstone	434	12.54	372	3.37	1.04	0.31
333	Eastern slope	T27	1920	K_1bt_1	mudstone	438	8.60	327	2.35	0.83	0.35
334	Eastern slope	T29	1915	K_1ba_4	mudstone	438	4.93	220	2.24	0.36	0.16
335	Eastern slope	T29	2052.9	K_1ba_4	mudstone	446	2.49	271	0.92	0.13	0.14
336	Eastern slope	T29	2035.5	K_1bt_1	mudstone	438	7.68	327	2.35	0.83	0.35
337	Eastern slope	T39	1965.3	K_1bt_1	mudstone	438	10.92	247	4.42	0.06	0.01
338	Eastern slope	T39	1775.2	K_1bt_1	mudstone	437	3.24	225	1.44	0.16	0.11
339	Eastern slope	T39	1963.3	K_1bt_1	mudstone	439	15.48	355	4.36	0.07	0.02
340	Eastern slope	T39	2112.5	K_1bt_1	mudstone	443	11.53	309	3.73	0.73	0.20
341	Eastern slope	T39	1778.2	K_1bt_1	mudstone	439	3.10	199	1.56	0.01	0.01
342	Eastern slope	T39	2374	K_1bt_1	mudstone	436	7.55	265	2.85	0.18	0.06
343	Eastern slope	T39	2177.5	K_1bt_1	mudstone	438	6.94	264	2.63	0.92	0.35
344	Eastern slope	T39	2312.5	K_1bt_1	mudstone	436	7.02	265	2.65	0.32	0.12
345	Eastern slope	T39	1772.7	K_1bt_1	mudstone	441	4.75	198	2.40	0.04	0.02
346	Eastern slope	T43	1825.3	K_1bt_1	mudstone	440	4.19	173	2.42	0.85	0.35
347	Eastern slope	T43	1924	K_1bt_1	mudstone	437	5.21	217	2.40	0.32	0.13

348	Eastern slope	T51	2390	K_1bt_1	mudstone	439	6.25	243	2.57	0.52	0.20
349	Eastern slope	T5	2568	K_1bt_1	mudstone	444	5.48	245	2.24		
350	Eastern slope	T5	2634	K_1ba_4	mudstone	447	4.67	223	2.09		
351	Eastern slope	TC1	1619	K_1ba_4	mudstone	435	9.10	196	4.63		
352	Eastern slope	T21	1575	K_1ba_4	mudstone	440	7.22	349	2.07		
353	Eastern slope	T21	1650	K_1bt_1	mudstone	443	7.51	222	3.39		
354	Eastern slope	T21	1900	K_1bt_1	mudstone	448	5.71	286	2.00		
355	Eastern slope	T21	2000	K_1bt_1	mudstone	451	5.38	273	1.97		
356	Eastern slope	T21	2171.9	K_1ba_4	mudstone	442	2.83	295	0.96	0.09	0.09
357	Eastern slope	T21	2264.2	K_1ba_4	mudstone	441	8.40	291	2.89	0.47	0.16
358	Eastern slope	T23	2145	K_1bt_1	mudstone	438	9.82	334	2.94		
359	Eastern slope	T23	2445	K_1ba_4	mudstone	438	6.86	302	2.27		
360	Eastern slope	T3	1261.1	K_1bt_1	mudstone	440	14.66	387	3.79		
361	Eastern slope	T3	1264.6	K_1bt_1	mudstone	431	11.76	230	5.11		
362	Eastern slope	T3	1267.3	K_1bt_1	mudstone	437	3.49	154	2.26		
363	Eastern slope	T3	1398.3	K_1bt_1	mudstone	437	4.64	239	1.94		
364	Eastern slope	T3	1399.5	K_1bt_1	mudstone	437	7.72	305	2.53		
365	Eastern slope	T3	1401.3	K_1bt_1	mudstone	440	5.40	286	1.89		
366	Eastern slope	T3	1403.1	K_1bt_1	mudstone	438	7.51	329	2.28		
367	Eastern slope	T3	1270	K_1ba_4	mudstone	436	7.55	265	2.85	0.18	0.06
